# Supplementary material for: Effect of Temperature and Light Intensity on the Polar Lipidome of Endophytic Brown Algae Streblonema corymbiferum and Streblonema sp. In Vitro
Source: Mar Drugs. 2022 Jun 29;20(7):428. doi: 10.3390/md20070428 (PMC9320489; doi:10.3390/md20070428)

Supplementary data content page

Title: Effect of Temperature and Light Intensity on the Polar Lipidome of Endophytic Brown Algae *Streblonema corymbiferum* and *Streblonema* sp. in Vitro

Authors: Oksana Chadova, Anna Skriptsova and Peter Velansky

Address: A.V. Zhirmunsky National Scientific Center of Marine Biology, Far Eastern Branch of Russian Academy of Sciences, Vladivostok 690041, Russia

Correspondence: chadova_9595@mail.ru

Table S1. Molecular species composition of MGDG, DGDG, SQDG, PG, PE, PC, PI, PHEG and DGTS of *Streblonema corymbiferum* and *Streblonema* sp. at various cultivation temperatures. Values given as % of total lipid class, (mean ± s.d. for triplicate). “+” – amount less than 0.1%, “-” – not detected. Between columns result of Tukey test (p<0.05, n=3) are shown (“>”, “<” – statistically significant difference, “=” – no significant changes), “T” – difference between 5 and 25 ˚C.

MGDG	*Streblonema corymbiferum*											*Streblonema* sp.									
	5 ˚C		10 ˚C		15 ˚C		20 ˚C		25 ˚C	T	#	5 ˚C		10 ˚C		15 ˚C		20 ˚C		25 ˚C	T
16:2/14:0	+	>	-		-	<	+	=	+	=		-		-	<	+	>	+	<	+	<
14:0/16:1	+	=	+	=	+	=	+	=	+	=		+	=	+	=	+	=	+	=	+	=
14:0/16:0	+	=	+	=	+	=	+	=	+	=		+	=	+	=	+	=	+	=	+	=
16:3/16:4	+	<	+	=	+	=	+	=	+	=		+	=	+	=	+	=	+	>	-	>
16:3/16:3	0.1±0.0	<	0.4±0.0	>	0.2±0.1	>	+	=	+	>		0.1±0.0	=	0.1±0.1	>	+	=	+	=	+	=
16:2/16:3	0.1±0.0	<	0.4±0.1	=	0.5±0.3	=	0.6±0.2	=	0.6±0.3	<		0.1±0.1	=	0.4±0.2	=	0.4±0.2	=	0.4±0.2	=	0.5±0.2	=
14:1/18:4	+	<	0.1±0.1	=	0.1±0.1	>	+	=	+	=		+	=	0.1±0.0	=	+	=	+	=	+	=
18:3/14:1	+	=	+	=	+	<	+	=	+	=		+	=	+	=	0.1±0.1	=	0.1±0.2	=	+	=
16:1/16:3	0.1±0.0	<	0.5±0.2	=	0.7±0.3	=	1.0±0.3	<	2.4±0.1	<		0.1±0.0	=	0.2±0.1	=	0.4±0.2	<	0.9±0.2	=	1.6±0.0	<
14:0/18:4	+	<	0.1±0.0	=	0.1±0.0	=	0.1±0.0	=	0.1±0.1	=		0.2±0.1	=	0.1±0.1	=	0.1±0.0	=	0.1±0.1	=	0.1±0.1	=
16:1/16:2	0.2±0.2	=	0.3±0.2	=	0.1±0.0	=	0.1±0.1	=	0.3±0.2	=		0.1±0.0	=	0.1±0.1	=	+	=	+	=	0.1±0.0	=
14:0/18:3	0.3±0.2	=	0.5±0.3	=	0.5±0.1	=	0.4±0.2	=	0.5±0.3	=		0.7±0.1	=	0.6±0.2	=	0.5±0.0	>	0.2±0.1	=	0.3±0.1	>
16:0/16:3	0.2±0.3	=	+	>	+	=	+	=	+	>		+	=	+	>	+	=	+	=	+	=
16:1/16:1	+	=	+	=	0.1±0.0	=	0.1±0.0	=	0.2±0.1	<		+	=	+	=	+	<	0.2±0.1	=	0.2±0.0	<
14:0/18:2	0.3±0.2	>	0.1±0.0	=	0.2±0.1	=	0.1±0.0	=	0.1±0.0	>		0.4±0.2	>	0.1±0.0	=	0.2±0.1	>	0.1±0.0	=	0.1±0.0	>
14:0/18:1	1.2±0.2	<	2.0±0.1	<	2.2±0.1	=	2.0±0.3	=	2.0±0.4	<		1.1±0.1	=	1.0±0.5	=	1.4±0.2	>	1.0±0.0	=	0.9±0.0	=
16:0/16:1	+	=	+	<	+	=	+	=	+	=		+	=	+	=	+	=	+	=	+	=
16:0/16:0	+	=	+	=	+	=	+	=	+	=		+	=	+	=	+	=	+	=	+	=
16:1/17:3	+	=	+	=	+	>	+	=	+	=		+	=	+	=	+	=	+	=	+	=
15:0/18:4	+	<	+	=	+	=	+	=	+	=		+	>	+	=	+	=	+	=	+	=
15:0/18:4	0.1±0.0	=	0.1±0.0	>	+	<	+	>	+	>		0.1±0.1	=	0.1±0.0	=	0.1±0.0	=	0.1±0.0	=	0.1±0.0	=
15:0/18:3	0.3±0.1	=	0.2±0.0	>	0.1±0.0	>	+	=	0.1±0.0	>		0.2±0.1	=	0.2±0.1	=	0.2±0.1	=	0.1±0.0	=	0.1±0.1	>
15:0/18:2	+	=	+	<	+	=	+	=	+	=		+	=	+	=	+	=	+	=	+	=
18:1/15:0	-	<	+	=	+	=	+	=	+	=		+	=	+	=	+	=	+	=	+	=
15:0/18:1	+	=	+	=	+	=	+	=	+	=		+	=	+	=	+	>	+	=	+	=
15:0/18:1	+	=	0.1±0.0	=	0.1±0.0	>	+	=	+	=		+	=	0.1±0.0	=	0.1±0.0	=	+	=	+	=
17:1/16:0	+	=	+	=	+	=	+	=	+	=		+	=	+	=	+	=	+	=	+	=
16:4/18:4	0.1±0.1	=	0.1±0.0	=	0.1±0.0	=	+	=	+	>		0.1±0.1	>	+	=	+	=	+	>	+	>
16:3/18:4	+	=	+	<	0.1±0.0	=	0.1±0.0	=	+	=		0.1±0.0	=	+	<	0.1±0.0	>	+	=	+	=
18:3/16:4	0.1±0.0	=	+	=	+	=	+	=	+	=		+	=	+	=	+	=	+	=	+	=
18:3/16:3	+	=	0.1±0.0	=	0.1±0.1	=	0.1±0.1	=	0.1±0.0	=		0.1±0.1	=	0.1±0.1	=	0.1±0.1	=	0.1±0.1	=	0.1±0.1	=
16:2/18:4	+	=	+	=	0.1±0.0	=	+	=	0.1±0.1	=		0.1±0.0	=	+	=	+	=	0.1±0.0	=	0.1±0.0	=
16:2/18:3	+	=	+	=	+	=	+	=	+	=		+	=	+	=	+	=	+	=	0.1±0.0	=
18:4/16:1	+	<	0.1±0.0	=	0.1±0.1	=	0.1±0.0	=	0.2±0.1	<		+	=	0.1±0.0	=	0.1±0.0	=	0.1±0.1	=	0.2±0.1	=
16:1/18:4	+	=	+	<	0.1±0.0	=	0.2±0.2	=	0.1±0.1	=		0.1±0.0	=	0.1±0.0	=	0.1±0.1	=	0.1±0.1	=	0.2±0.1	=
18:1/16:4	0.1±0.1	=	0.1±0.0	=	0.1±0.1	=	0.2±0.1	=	0.1±0.1	=		0.1±0.0	=	+	=	+	<	0.3±0.1	=	0.3±0.2	=
20:5/14:0	0.1±0.0	=	0.1±0.0	=	0.1±0.0	=	0.1±0.1	=	0.1±0.1	=		+	=	+	=	+	=	+	=	+	=
14:0/20:5	+	=	0.1±0.0	=	0.1±0.1	=	0.1±0.1	=	0.1±0.1	=		0.1±0.1	=	+	=	+	=	+	=	+	=
18:5/16:0	+	=	0.1±0.1	=	0.1±0.0	=	0.1±0.0	=	0.1±0.1	=		+	=	+	=	0.1±0.0	=	+	=	+	=
18:3/16:1	0.1±0.0	=	0.1±0.0	=	0.2±0.1	=	0.2±0.1	=	0.2±0.0	=		0.1±0.0	=	0.1±0.0	=	0.1±0.0	<	0.2±0.0	=	0.3±0.0	<
16:1/18:3	+	<	0.1±0.0	=	0.1±0.0	=	0.1±0.0	=	0.2±0.1	<		+	<	0.1±0.0	=	0.1±0.1	=	0.1±0.0	=	0.1±0.0	=
18:1/16:3	+	=	+	=	0.1±0.1	=	0.1±0.1	=	0.1±0.1	=		0.1±0.0	=	0.1±0.0	=	+	=	+	=	0.1±0.0	=
14:0/20:4	0.2±0.0	=	0.1±0.1	=	0.2±0.1	=	0.1±0.0	=	0.1±0.1	=		0.1±0.1	=	0.3±0.2	=	0.1±0.1	=	0.2±0.1	=	0.1±0.1	=
16:0/18:4	0.1±0.1	=	0.1±0.1	=	0.1±0.0	=	0.1±0.0	=	0.1±0.0	=		0.1±0.1	=	0.1±0.1	=	0.1±0.1	=	0.3±0.2	=	0.1±0.0	=
18:2/16:1	+	<	+	=	+	=	+	<	0.1±0.0	<		+	=	+	=	+	=	0.1±0.1	=	0.1±0.0	=
16:1/18:2	+	<	+	=	+	=	+	=	0.1±0.0	<		+	=	+	=	+	=	0.1±0.0	=	+	<
18:1/16:2	+	=	+	<	+	=	+	=	+	=		+	=	+	>	+	=	+	=	+	=
16:0/18:3	1.9±0.4	>	1.3±0.0	>	0.5±0.1	=	0.5±0.3	=	0.5±0.2	>		2.6±0.3	=	2.5±0.4	=	2.3±0.5	=	1.9±0.1	=	1.4±0.0	>
18:0/16:3	+	=	+	=	+	=	+	=	+	=		+	=	+	=	+	=	+	=	+	=
18:1/16:1	+	=	+	=	+	=	+	=	+	=		+	=	+	=	+	>	+	=	+	=

18:1/16:1	0.2±0.1	=	0.2±0.1	=	0.1±0.0	>	0.1±0.0	=	0.1±0.1	=	0.3±0.0	>	0.2±0.1	=	0.1±0.0	=	0.3±0.0	=	0.1±0.0	>
16:0/18:2	0.3±0.1	=	0.2±0.1	=	0.1±0.0	=	0.1±0.1	=	0.1±0.0	>	1.0±0.1	>	0.5±0.1	<	1.2±0.4	=	0.8±0.2	>	0.4±0.0	>
16:0/18:1	1.6±0.1	=	1.6±0.1	=	1.6±0.2	>	1.1±0.2	=	1.2±0.3	=	2.2±0.2	<	2.9±0.1	<	3.9±0.3	>	2.4±0.4	=	2.1±0.2	=
16:0/18:0	0.1±0.1	=	+	=	+	=	+	=	+	>	0.1±0.1	=	+	=	0.2±0.2	=	+	=	+	=
18:3/17:3	+	=	+	<	+	=	+	=	+	=	+	=	+	=	+	=	+	=	+	=
18:4/17:2	+	=	+	=	+	=	+	=	+	=	+	=	+	=	+	=	+	=	+	=
17:2/18:4	+	=	+	=	+	>	-	<	+	>	+	=	+	=	+	=	+	=	+	=
17:2/18:3	+	=	+	=	+	=	+	=	+	=	+	=	+	=	+	=	+	=	+	=
17:1/18:3	+	=	+	=	+	=	+	=	+	=	+	=	+	=	+	=	+	=	+	=
15:0/20:4	+	=	+	=	+	=	+	=	+	=	+	=	+	=	+	>	+	=	+	=
17:0/18:3	+	=	+	=	+	=	+	<	+	=	+	=	+	=	+	=	+	=	+	=
18:1/17:1	+	<	+	=	+	=	+	=	+	=	+	=	+	>	+	=	+	=	+	=
17:0/18:2	+	=	+	=	+	=	+	<	+	=	+	=	+	=	+	=	+	=	+	=
17:0/18:1	+	<	+	=	+	>	+	=	+	=	+	=	+	=	+	>	+	=	+	=
20:5/16:4	0.6±0.1	<	0.9±0.1	=	0.8±0.1	>	0.4±0.2	=	0.3±0.1	>	0.4±0.1	=	0.5±0.2	=	0.2±0.1	=	0.1±0.1	=	+	>
18:4/18:4	25.6±1.0	>	19.9±0.5	>	16.3±0.3	>	11.6±1.0	=	10.9±0.6	>	34.2±0.5	>	24.4±1.1	>	20.8±1.4	>	15.4±1.0	=	13.5±0.5	>
18:3/18:4	17.1±0.7	=	17.6±0.5	=	17.8±0.6	>	16.1±0.6	>	14.0±0.5	>	20.2±1.6	=	21.1±1.3	<	23.5±1.1	>	20.1±0.8	=	18.0±0.0	=
18:3/18:3	6.4±0.2	<	7.5±0.5	<	9.1±0.1	<	10.1±0.4	>	9.0±0.7	<	8.0±0.1	<	11.2±0.8	=	12.7±1.0	>	10.7±0.6	=	7.6±0.1	=
18:2/18:4	1.3±0.2	=	1.0±0.7	=	0.8±0.4	=	1.5±0.5	<	2.8±0.6	<	1.9±0.2	=	2.1±0.6	=	2.5±0.5	<	5.6±1.0	=	8.4±0.4	<
20:5/16:1	0.1±0.0	=	0.1±0.1	=	0.1±0.0	<	0.3±0.0	=	0.4±0.1	<	0.1±0.1	<	0.8±0.0	>	+	=	0.1±0.2	=	0.6±0.5	=
18:3/18:2	3.0±0.2	=	2.6±0.4	=	2.8±0.1	<	3.9±0.7	=	4.4±0.3	<	4.3±0.1	<	5.1±0.5	=	5.1±0.2	<	9.0±0.9	=	10.1±0.6	=
18:2/18:3	0.1±0.1	=	0.1±0.1	<	0.4±0.1	=	0.3±0.1	=	0.3±0.2	=	0.1±0.0	=	0.1±0.1	=	+	<	0.4±0.1	=	1.2±0.1	<
18:1/18:4	0.2±0.1	=	0.1±0.0	=	0.2±0.1	=	0.3±0.2	=	0.1±0.1	=	+	=	+	=	0.1±0.0	<	0.2±0.1	=	0.8±0.2	<
20:5/16:0	0.1±0.1	<	0.3±0.1	=	0.2±0.1	=	0.3±0.2	=	0.3±0.1	=	+	<	0.1±0.0	>	+	=	0.1±0.1	<	0.4±0.0	<
16:0/20:5	0.1±0.1	=	0.2±0.1	=	0.1±0.1	=	0.2±0.1	=	0.4±0.2	=	0.2±0.2	=	0.2±0.2	=	0.3±0.0	>	0.1±0.1	=	0.1±0.0	=
18:2/18:2	0.4±0.3	=	0.4±0.3	=	0.4±0.1	>	0.1±0.0	<	1.2±0.3	<	0.9±0.1	<	1.2±0.1	>	0.7±0.3	=	2.2±1.2	=	2.9±1.4	=
18:1/18:3	2.0±0.1	>	1.3±0.3	<	2.5±0.3	<	3.6±0.3	=	3.0±0.6	<	1.1±0.3	=	1.4±0.1	>	1.1±0.0	=	1.9±1.0	=	3.8±0.5	=
16:0/20:4	+	=	+	=	+	<	+	=	+	=	0.1±0.0	=	+	=	+	=	+	=	+	=
16:0/20:4	0.1±0.0	=	+	=	+	>	+	<	0.1±0.0	=	0.1±0.0	=	0.1±0.0	=	0.1±0.0	=	0.1±0.0	=	0.1±0.1	=
18:1/18:2	0.5±0.1	=	0.3±0.2	=	0.3±0.3	=	0.3±0.1	<	0.5±0.1	=	0.4±0.2	=	0.5±0.0	=	0.5±0.1	=	0.6±0.1	=	0.8±0.0	=
20:3/16:0	+	=	+	<	+	=	0.1±0.0	=	+	=	+	=	+	=	+	=	+	=	+	=
18:0/18:3	+	=	0.1±0.0	=	+	=	+	=	+	=	0.1±0.1	=	0.1±0.0	=	0.1±0.0	>	0.1±0.0	>	+	=
18:1/18:1	+	<	+	<	0.2±0.0	>	+	=	0.1±0.1	=	+	=	+	=	+	=	+	=	+	=
18:1/18:1	0.1±0.0	=	0.1±0.0	=	0.1±0.1	=	0.1±0.0	=	0.1±0.1	=	0.1±0.0	<	0.1±0.0	=	0.1±0.0	>	+	=	+	=
18:0/18:2	0.1±0.1	=	+	=	+	=	+	=	0.1±0.1	=	+	=	+	=	+	=	+	=	+	=
18:0/18:1	0.1±0.0	=	0.1±0.0	=	0.1±0.0	=	0.1±0.0	>	+	>	0.1±0.0	=	0.1±0.0	=	0.1±0.0	=	0.1±0.0	=	0.1±0.0	=
18:0/18:0	+	>	-	<	+	>	+	>	-	>	+	=	+	>	-	<	+	=	+	=
18:4/19:2	+	=	+	=	+	=	+	=	+	=	+	=	+	=	+	=	+	=	+	=
19:2/18:4	+	=	+	=	+	=	+	=	+	=	+	=	+	=	+	=	+	=	+	=
18:3/19:2	+	=	+	=	+	=	+	=	+	=	+	<	+	=	+	=	+	=	+	<
19:2/18:3	+	=	+	=	+	=	+	=	+	=	+	=	+	>	-	<	+	>	+	=
18:4/19:1	+	=	+	=	+	=	+	=	+	=	+	=	+	>	-	=	+	=	+	=
18:3/19:1	+	=	+	=	+	=	+	=	+	=	+	=	+	=	+	=	+	<	+	=
20:5/18:4	18.6±0.7	=	18.0±0.1	>	17.0±0.4	>	14.3±1.2	>	12.1±0.5	>	9.7±1.1	>	8.4±0.1	>	7.3±0.6	>	6.1±0.3	=	4.3±0.6	>
20:5/18:3	6.7±0.1	<	8.9±0.5	<	11.0±0.5	<	12.3±0.8	<	13.5±0.8	<	2.3±0.2	<	5.7±0.2	=	5.5±0.1	=	5.0±0.4	=	3.8±0.6	=
20:4/18:4	1.6±0.1	=	1.5±0.3	=	1.6±0.7	=	1.8±0.2	>	1.2±0.4	=	0.7±0.2	=	0.8±0.0	=	0.8±0.3	<	1.7±0.2	=	1.6±0.4	=
20:4/18:3	0.3±0.1	=	0.5±0.1	<	0.6±0.1	=	0.5±0.2	=	0.8±0.3	<	0.4±0.0	=	0.5±0.1	=	0.5±0.1	=	0.4±0.1	=	0.3±0.2	=
20:5/18:2	1.5±0.2	<	2.4±0.2	>	1.2±0.5	<	4.2±0.1	<	5.5±0.4	<	1.5±0.1	<	2.0±0.2	=	1.8±0.2	<	3.4±0.3	=	4.0±0.0	=
20:3/18:3	0.2±0.0	=	0.3±0.2	=	0.2±0.0	=	0.2±0.1	=	0.2±0.1	=	0.2±0.1	=	0.3±0.1	=	0.2±0.1	=	0.4±0.3	=	0.6±0.0	=
20:4/18:2	+	=	0.2±0.2	=	0.3±0.1	=	0.2±0.1	=	0.2±0.1	=	0.1±0.0	<	0.1±0.0	=	0.1±0.0	<	0.3±0.0	=	0.4±0.2	<
20:5/18:1	1.0±0.2	<	2.0±0.1	<	2.2±0.1	<	3.8±0.2	<	4.4±0.3	<	0.1±0.0	=	0.3±0.2	=	0.3±0.1	=	0.3±0.1	=	0.3±0.0	=
20:3/18:2	+	=	+	=	+	=	+	=	0.1±0.0	=	+	=	+	=	0.1±0.0	=	0.2±0.2	=	0.1±0.1	=
20:4/18:1	0.1±0.1	=	0.2±0.1	=	0.1±0.2	<	0.4±0.1	=	0.2±0.1	=	0.1±0.0	=	0.1±0.0	=	0.1±0.0	=	0.2±0.1	=	0.6±0.3	<
20:4/18:1	0.1±0.0	=	0.2±0.1	=	0.2±0.1	<	0.5±0.1	=	0.5±0.2	<	+	=	0.1±0.0	=	+	<	0.1±0.0	=	0.2±0.0	<
20:5/18:0	0.1±0.1	=	+	=	+	=	0.1±0.1	=	+	=	+	=	+	=	+	=	+	=	+	=
20:3/18:1	0.1±0.1	=	0.1±0.0	=	+	=	0.1±0.0	=	0.1±0.0	=	+	=	+	=	0.1±0.0	=	+	=	0.1±0.0	=
20:5/20:5	1.0±0.3	>	0.6±0.1	=	0.5±0.2	>	0.2±0.1	=	0.2±0.1	>	0.6±0.2	=	0.5±0.3	=	0.6±0.2	=	0.5±0.3	=	0.5±0.1	=
20:5/20:4	0.1±0.1	=	0.1±0.1	=	+	=	0.1±0.1	=	+	>	0.1±0.1	=	+	=	0.1±0.0	=	0.1±0.0	=	0.1±0.0	=
20:4/20:5	1.4±0.2	=	1.7±0.3	=	1.9±0.1	>	1.5±0.2	>	0.6±0.2	>	0.8±0.2	<	1.1±0.1	=	1.3±0.3	<	2.0±0.1	=	1.9±0.1	=
20:3/20:5	0.1±0.0	=	0.1±0.0	<	0.3±0.0	=	0.2±0.0	=	0.2±0.0	=	0.1±0.1	=	0.1±0.0	=	0.1±0.1	=	0.1±0.0	>	0.1±0.0	=
20:4/20:4	0.6±0.1	=	1.0±0.4	=	1.3±0.1	=	1.6±0.3	=	1.3±0.4	<	0.2±0.1	=	0.4±0.2	=	0.6±0.2	<	1.8±0.1	=	2.6±0.3	<
20:4/20:3	+	=	+	=	+	<	+	=	+	=	+	=	+	=	+	=	+	=	+	=
20:3/20:4	+	=	+	=	+	=	+	=	+	=	+	=	+	=	+	=	+	=	+	=

DGDG	*Streblonema corymbiferum*											*Streblonema* sp.									
	5 °C		10 °C		15 °C		20 °C		25 °C	T	#	5 °C		10 °C		15 °C		20 °C		25 °C	T
14:0/16:1	+	<	0.1±0.0		0.1±0.0	=	0.1±0.1	=	0.1±0.0	=		+	=	0.1±0.0	=	0.1±0.1	=	0.1±0.0	=	0.1±0.1	<
14:0/16:0	+	=	+	=	+	=	+	<	+	<		+	=	+	=	+	=	+	=	0.1±0.0	<
16:1/16:4	0.1±0.0	>	+	=	0.1±0.0	=	+	=	+	=		+	=	+	=	+	=	+	>	-	>
16:3/16:1	+	=	+	=	+	=	+	=	+	=		0.1±0.0	>	+	=	+	<	0.1±0.0	=	+	=
14:0/18:4	0.2±0.2	=	0.2±0.0	>	0.1±0.0	=	+	=	0.1±0.0	>		0.5±0.5	=	0.3±0.0	>	0.1±0.1	=	0.1±0.1	=	0.1±0.0	=
16:1/16:2	0.1±0.0	=	0.1±0.0	=	0.1±0.0	=	+	=	+	=		0.1±0.1	=	0.1±0.1	=	0.1±0.0	>	+	=	0.1±0.1	=
14:0/18:3	3.3±0.6	=	2.4±0.5	=	3.1±0.6	>	2.0±0.2	=	2.4±0.4	>		3.5±0.3	>	1.5±1.1	=	2.1±0.2	>	0.8±0.4	=	0.5±0.1	>
16:1/16:1	0.1±0.0	=	0.1±0.0	<	0.2±0.0	=	0.1±0.1	<	0.4±0.1	<		0.1±0.0	=	0.1±0.1	=	0.2±0.1	=	0.3±0.2	=	0.3±0.3	=
14:0/18:2	0.4±0.2	=	0.4±0.1	=	0.3±0.2	=	0.3±0.1	=	0.3±0.1	=		0.6±0.3	=	0.2±0.2	=	0.2±0.2	=	0.7±0.4	=	0.8±0.2	=
14:0/18:1	0.5±0.1	<	1.6±0.4	<	2.4±0.5	=	2.9±0.4	=	2.9±0.3	<		0.2±0.1	=	0.3±0.0	=	0.4±0.2	=	0.6±0.5	=	0.6±0.2	<
16:0/16:1	0.8±0.1	<	1.4±0.4	=	1.2±0.5	=	0.8±0.1	<	1.6±0.4	<		+	=	0.1±0.1	=	0.2±0.3	=	+	=	0.4±0.6	=
16:0/16:0	0.1±0.0	>	+	>	+	>	-	<	+	>		+	=	+	=	+	=	+	=	+	=
15:0/18:4	+	=	+	=	+	=	+	=	+	=		0.1±0.0	=	+	=	+	=	+	=	+	>
15:0/18:3	0.1±0.0	=	+	<	0.1±0.0	=	0.1±0.1	=	+	=		0.1±0.1	=	0.1±0.0	=	0.1±0.1	=	0.1±0.1	=	+	=
15:0/18:2	+	=	+	=	+	=	+	=	+	=		0.1±0.0	>	+	=	+	=	+	<	0.1±0.0	=
15:0/18:1	+	<	0.1±0.0	=	0.1±0.0	=	0.1±0.0	=	0.1±0.1	=		+	=	+	=	+	=	0.1±0.0	=	0.1±0.0	<
16:3/18:3	0.1±0.1	=	+	=	+	=	+	=	0.1±0.1	=		+	=	0.1±0.0	=	0.1±0.0	>	+	=	+	>
18:4/16:2	0.1±0.1	=	+	<	+	=	+	=	+	=		+	=	+	=	+	=	0.1±0.0	=	0.1±0.1	=
16:2/18:3	+	=	+	=	+	=	+	=	+	=		+	=	+	=	+	=	0.1±0.0	=	0.1±0.0	=
18:4/16:1	0.1±0.0	>	+	=	+	=	+	=	0.1±0.1	=		+	=	0.1±0.0	>	+	=	0.1±0.1	=	0.1±0.0	=
20:5/14:0	0.1±0.1	=	+	<	0.2±0.1	=	0.2±0.0	=	0.3±0.2	=		+	=	+	=	0.1±0.0	=	0.1±0.1	=	0.1±0.1	=
16:1/18:3	0.4±0.2	=	0.2±0.1	=	0.2±0.1	=	0.2±0.1	=	0.3±0.1	=		0.2±0.1	=	0.2±0.1	=	0.1±0.1	=	0.2±0.1	=	0.3±0.2	=
16:0/18:4	0.7±0.1	>	0.3±0.1	=	0.2±0.1	>	0.1±0.1	=	0.1±0.0	>		0.3±0.1	=	0.2±0.1	=	0.2±0.1	=	0.2±0.1	=	0.1±0.1	=
16:1/18:2	+	=	+	=	+	=	+	<	+	>		0.1±0.0	>	+	=	0.1±0.1	=	+	>	+	>
14:0/20:3	0.1±0.0	>	+	=	0.1±0.0	=	0.1±0.1	=	+	=		0.1±0.1	=	0.1±0.1	=	0.1±0.0	=	0.1±0.1	=	+	=
16:0/18:3	2.9±0.5	>	1.4±0.3	<	2.4±0.3	>	0.8±0.2	=	1.4±0.6	>		5.4±0.6	<	6.9±0.9	>	5.7±0.2	=	5.5±0.9	=	4.3±0.4	>
16:0/18:2	0.3±0.2	=	0.2±0.0	=	0.2±0.0	=	0.2±0.1	=	0.2±0.0	=		0.6±0.3	=	0.4±0.2	<	1.2±0.2	>	0.7±0.1	=	0.9±0.3	=
16:0/18:1	0.6±0.1	<	1.0±0.2	=	1.1±0.1	=	0.8±0.4	=	1.1±0.4	=		0.4±0.3	<	1.0±0.3	<	2.2±0.5	=	2.6±0.1	=	1.9±0.7	<
20:5/16:4	0.1±0.1	=	0.1±0.0	=	+	=	+	=	+	>		+	=	0.1±0.0	=	+	=	+	>	+	>
18:4/18:4	3.1±0.9	=	2.4±0.2	>	1.9±0.2	>	1.1±0.3	>	0.4±0.1	>		2.8±0.1	=	2.1±0.7	=	1.4±0.6	=	1.2±0.2	=	1.2±0.4	>
18:3/18:4	1.4±0.3	<	2.8±1.0	>	0.8±0.4	=	1.5±0.4	>	0.8±0.3	=		1.7±0.8	<	3.6±0.6	=	4.0±0.4	>	2.0±0.7	<	4.2±0.7	<
20:5/16:2	1.7±0.3	=	1.8±0.5	=	2.4±0.2	=	2.6±0.5	>	1.1±0.4	=		1.4±0.8	=	1.0±0.6	=	0.5±0.3	<	4.4±1.3	>	1.9±0.7	=
16:3/20:4	+	=	+	=	+	=	0.2±0.3	=	+	=		+	=	+	=	+	=	+	=	0.1±0.2	=
18:3/18:3	7.0±0.3	=	6.6±0.3	>	6.1±0.1	<	6.9±0.6	<	8.2±0.8	=		8.3±1.3	<	10.4±0.3	=	12.1±1.5	=	13.6±1.2	>	9.9±0.4	=
20:5/16:1	0.5±0.1	=	0.4±0.1	=	0.4±0.2	<	1.6±0.3	<	3.6±0.8	<		0.3±0.1	>	0.1±0.0	=	0.4±0.3	<	1.8±0.6	=	3.9±1.8	<
18:3/18:2	1.5±0.2	>	0.8±0.3	=	0.8±0.3	=	0.5±0.1	<	1.0±0.2	>		2.0±0.2	<	2.8±0.3	>	1.7±0.4	<	5.8±0.1	<	8.5±0.9	<
20:4/16:1	0.1±0.0	=	+	=	0.1±0.0	=	0.1±0.1	=	0.1±0.1	=		0.1±0.0	=	+	=	0.1±0.0	=	0.1±0.0	=	0.2±0.3	=
20:5/16:0	0.5±0.1	>	0.3±0.2	=	0.4±0.1	=	0.7±0.3	<	1.7±0.3	<		0.1±0.1	=	0.1±0.0	=	0.1±0.1	=	0.2±0.1	<	0.5±0.0	<
18:2/18:2	0.1±0.0	=	0.2±0.3	=	+	=	+	<	0.2±0.1	<		0.2±0.1	=	0.2±0.1	=	0.1±0.1	<	0.6±0.2	=	0.8±0.7	=
18:3/18:1	0.4±0.1	<	1.8±0.2	>	0.9±0.7	=	1.3±0.8	=	1.4±0.9	=		0.4±0.0	=	0.5±0.3	=	0.4±0.1	<	1.1±0.5	=	1.1±0.4	<
20:4/16:0	0.3±0.3	=	+	=	+	=	0.4±0.6	=	0.1±0.1	=		0.1±0.1	=	0.2±0.1	>	+	=	0.1±0.1	=	0.4±0.7	=
16:0/20:4	+	=	+	=	+	=	+	=	+	<		+	=	+	=	+	=	+	=	+	=
18:2/18:1	0.1±0.0	=	+	=	+	=	0.1±0.0	=	0.1±0.1	=		+	<	0.1±0.0	=	0.1±0.0	=	0.1±0.1	=	0.1±0.0	=
20:3/16:0	0.1±0.0	>	+	=	+	=	+	=	+	=		+	=	0.1±0.1	=	+	<	0.1±0.1	=	0.1±0.1	<
16:0/20:3	+	=	+	=	+	=	0.1±0.1	=	+	=		0.1±0.0	=	0.1±0.0	=	0.3±0.2	=	0.1±0.0	=	0.1±0.1	=
18:0/18:3	+	=	0.1±0.0	=	0.1±0.0	>	+	=	+	=		0.1±0.1	<	0.4±0.2	=	0.4±0.3	=	0.2±0.1	=	0.1±0.0	=
18:1/18:1	0.1±0.1	=	+	=	+	=	+	=	0.1±0.0	=		+	=	+	=	+	=	+	=	0.1±0.0	=
18:0/18:2	+	=	+	=	+	=	+	<	+	=		+	=	+	=	+	=	0.1±0.0	=	+	=
18:0/18:1	+	=	0.1±0.0	=	0.1±0.0	=	0.1±0.0	=	+	=		0.1±0.0	=	0.1±0.1	=	0.1±0.1	=	0.2±0.1	=	0.1±0.0	=
18:2/19:3	+	=	+	=	0.1±0.1	=	+	=	0.1±0.0	=		0.1±0.1	=	0.1±0.1	=	0.2±0.2	=	0.1±0.1	=	0.3±0.1	<
18:3/19:1	+	=	+	=	+	=	+	=	+	=		0.1±0.1	=	+	=	0.1±0.0	=	0.1±0.1	=	0.1±0.1	=
20:5/18:4	55.2±4.6	>	47.3±1.0	>	40.9±1.7	>	32.1±1.8	>	21.7±0.8	>		55.2±4.3	>	45.6±1.9	>	37.0±1.7	>	26.2±1.1	>	20.0±2.2	>
20:5/18:3	9.6±1.2	<	16.7±0.5	<	21.7±1.1	<	29.4±0.8	<	31.3±0.3	<		9.2±0.9	<	14.6±0.4	<	21.6±1.2	>	15.4±0.4	=	15.9±1.7	<
20:4/18:4	5.2±1.2	=	4.5±0.9	=	4.6±0.5	>	2.6±0.5	=	2.5±0.9	>		3.7±1.5	=	3.5±0.1	>	2.4±0.6	<	5.1±0.3	=	3.8±1.9	=
20:4/18:3	1.1±0.1	=	1.1±0.3	<	2.0±0.3	=	1.7±0.4	<	5.2±0.9	<		0.6±0.4	=	1.1±0.6	=	1.3±0.2	<	5.9±0.6	=	6.6±2.3	<
20:5/18:2	0.5±0.1	<	1.1±0.4	=	0.8±0.2	<	3.2±0.2	=	2.0±1.2	<		0.6±0.3	=	1.0±0.6	=	1.6±0.3	=	1.1±0.3	<	5.0±1.6	<
20:3/18:3	+	=	0.1±0.0	=	+	=	+	=	+	=		+	=	+	=	+	=	+	<	0.1±0.0	<
20:4/18:2	0.1±0.0	=	0.1±0.0	=	0.7±0.7	=	0.2±0.1	=	0.2±0.2	=		0.1±0.0	=	0.1±0.0	=	0.1±0.1	<	0.6±0.1	<	2.0±0.5	<
20:5/18:1	0.3±0.2	<	1.8±0.3	=	2.5±0.7	<	4.6±0.6	<	6.2±0.4	<		+	<	0.2±0.1	=	0.2±0.1	<	1.2±0.2	=	1.7±0.5	<
18:3/20:2	+	=	+	=	+	=	+	=	+	=		0.1±0.1	=	0.1±0.1	=	+	=	0.1±0.1	=	+	=
20:4/18:1	0.1±0.1	=	+	=	+	=	0.1±0.0	=	0.2±0.1	=		+	=	+	=	+	=	0.1±0.0	=	0.2±0.1	<
20:3/18:1	+	=	+	=	+	=	+	=	+	=		+	=	+	=	0.1±0.0	=	+	=	+	=
18:1/20:2	+	=	+	=	+	=	+	=	+	<		+	=	+	=	+	=	+	=	+	=

SQDG	*Streblonema corymbiferum*											*Streblonema* sp.									
	5 ˚C		10 ˚C		15 ˚C		20 ˚C		25 ˚C	T	#	5 ˚C		10 ˚C		15 ˚C		20 ˚C		25 ˚C	T
14:0/14:0	0.1±0.0	<	0.3±0.1		0.3±0.1	=	0.5±0.3	=	0.9±0.2	<		0.3±0.1	=	0.4±0.3	=	0.1±0.1	<	0.4±0.1	=	0.5±0.5	=
14:0/16:1	0.3±0.1	=	0.5±0.2	<	0.7±0.1	=	1.1±0.4	<	2.4±0.2	<		0.4±0.1	=	0.5±0.2	=	0.3±0.2	<	0.8±0.3	=	1.4±0.5	<
14:0/16:0	1.0±0.0	<	1.5±0.2	<	2.2±0.1	<	4.8±1.2	<	8.0±0.6	<		1.2±0.6	<	2.4±0.1	<	3.7±0.9	<	7.2±1.0	<	10.8±0.5	<
16:1/15:0	+	=	0.1±0.1	=	0.1±0.0	=	0.1±0.1	=	0.2±0.2	=		0.1±0.1	=	0.1±0.0	=	0.1±0.1	=	0.1±0.1	=	0.1±0.1	=
16:0/15:0	0.1±0.0	>	+	=	+	<	0.1±0.0	=	0.1±0.1	=		0.1±0.0	=	0.1±0.0	=	0.1±0.0	=	0.2±0.1	=	0.3±0.2	=
14:0/18:3	5.0±1.2	=	3.6±0.2	>	2.6±0.3	>	1.8±0.2	>	1.2±0.1	>		2.4±0.2	>	1.9±0.2	>	1.2±0.5	=	1.0±0.2	=	0.6±0.5	>
14:0/18:2	3.3±0.3	=	3.0±0.4	=	3.1±0.3	=	3.7±0.8	=	3.8±0.4	=		3.0±0.8	=	3.6±0.9	>	2.2±0.4	<	3.9±0.7	=	3.8±0.3	=
14:0/18:1, 16:0/16:1, 16:1/16:0	7.1±0.4	<	12.4±1.1	<	14.4±0.8	<	20.2±1.5	<	26.8±0.7	<		5.8±0.9	=	7.7±1.4	=	6.6±1.0	<	11.4±0.6	=	11.9±4.2	<
16:0/16:0	0.2±0.0	=	0.2±0.1	<	0.5±0.1	=	0.7±0.1	<	1.2±0.3	<		1.4±0.3	<	2.4±0.7	<	4.4±0.4	<	6.4±0.7	<	9.4±0.9	<
15:0/18:3	0.3±0.3	=	0.3±0.1	=	0.1±0.1	=	+	=	0.1±0.1	>		0.2±0.2	=	0.1±0.1	=	0.2±0.1	=	0.1±0.0	=	0.1±0.0	=
15:0/18:2	0.2±0.0	>	0.1±0.0	=	0.1±0.1	=	0.2±0.1	=	0.1±0.1	=		0.1±0.1	=	0.1±0.1	<	0.3±0.1	=	0.3±0.1	=	0.4±0.1	<
15:0/18:1	0.4±0.2	=	0.4±0.0	=	0.4±0.2	=	0.2±0.0	=	0.5±0.2	=		0.1±0.0	<	0.4±0.1	=	0.1±0.1	=	0.2±0.1	=	0.5±0.0	<
17:1/16:0	0.4±0.3	=	0.6±0.2	=	0.6±0.1	=	0.6±0.3	=	0.6±0.1	=		0.7±0.4	=	0.5±0.1	=	0.2±0.0	=	0.2±0.1	=	0.4±0.3	=
14:0/20:5	-	#	+	=	+	=	0.1±0.1	=	+	=		0.1±0.1	=	0.1±0.0	=	0.1±0.0	=	+	=	0.1±0.1	=
16:1/18:3	0.2±0.2	=	+	=	+	=	+	>	-	>		0.1±0.1	=	0.1±0.0	>	+	=	+	=	+	=
20:4/14:0	0.1±0.0	=	+	=	+	=	+	=	0.1±0.1	=		0.1±0.1	=	+	=	0.1±0.0	=	0.1±0.0	=	0.1±0.1	=
14:0/20:4	0.1±0.0	=	0.1±0.1	=	+	=	+	=	+	=		0.1±0.0	=	+	=	0.1±0.0	=	+	=	0.1±0.1	=
18:3/16:0	22.9±1.5	>	16.8±1.1	>	14.5±1.1	>	10.0±1.0	>	6.3±1.0	>		23.5±2.4	>	17.5±1.4	>	15.1±0.5	>	12.5±0.9	>	8.5±0.5	>
18:2/16:0	8.9±0.4	>	6.0±0.0	>	4.7±0.8	=	5.2±0.2	=	5.1±0.4	>		9.8±0.1	<	11.3±0.8	=	10.3±0.6	<	16.0±1.3	=	18.2±3.5	<
18:1/16:0	40.3±1.3	<	48.7±1.3	=	50.0±1.2	>	44.4±3.2	>	38.0±2.0	=		41.7±0.9	=	44.1±3.0	<	49.8±3.1	>	35.7±1.4	>	29.6±1.4	>
18:1/16:0	4.6±0.2	>	2.2±1.0	=	2.0±1.2	=	2.4±0.1	<	2.8±0.3	>		4.2±0.9	>	3.0±0.4	>	2.0±0.6	>	0.9±0.6	=	1.4±0.0	>
18:0/16:0	0.1±0.0	>	+	=	+	=	0.1±0.0	>	+	>		+	<	0.1±0.0	=	0.1±0.0	=	0.1±0.1	<	0.2±0.1	<
16:0/19:1	0.1±0.0	=	0.2±0.1	=	0.2±0.2	=	0.3±0.1	=	0.2±0.1	=		0.2±0.0	=	0.1±0.0	=	+	=	+	=	0.1±0.0	=
17:0/18:1	0.1±0.0	=	+	<	0.1±0.0	=	0.1±0.1	=	0.1±0.1	=		0.1±0.0	=	0.1±0.0	>	-	<	+	=	+	=
18:4/18:4	0.1±0.0	=	0.1±0.1	=	+	=	+	<	0.1±0.0	=		0.1±0.0	=	0.1±0.0	=	0.1±0.0	=	+	=	0.2±0.2	=
18:3/18:4	0.6±0.3	=	0.3±0.0	=	0.3±0.1	=	0.2±0.1	=	0.1±0.0	>		1.4±0.3	>	0.9±0.1	>	0.4±0.1	=	0.3±0.1	=	0.1±0.1	>
18:3/18:3	1.0±0.1	=	0.7±0.2	=	0.6±0.1	=	0.4±0.4	=	0.2±0.1	>		1.2±0.1	=	0.8±0.4	=	0.4±0.1	=	0.4±0.1	>	0.1±0.1	>
18:2/18:4	0.1±0.1	=	0.1±0.1	=	0.2±0.0	=	0.1±0.1	=	+	>		0.2±0.1	=	0.3±0.0	=	0.1±0.0	=	0.1±0.1	=	0.1±0.1	=
18:3/18:2	0.3±0.0	>	0.1±0.1	=	0.2±0.1	=	0.2±0.1	=	0.1±0.1	=		0.1±0.0	=	0.2±0.2	=	0.1±0.1	=	0.3±0.5	=	0.3±0.2	=
18:2/18:3	0.6±0.3	=	0.3±0.1	>	0.1±0.1	=	0.3±0.1	=	0.1±0.1	>		0.4±0.2	=	0.5±0.1	=	0.6±0.2	=	0.4±0.3	=	0.3±0.1	=
18:1/18:4	0.1±0.0	=	+	=	+	=	+	=	0.1±0.0	=		+	=	+	>	-	=	+	=	0.1±0.0	<
20:5/16:0	0.1±0.0	=	0.1±0.0	=	0.1±0.0	=	0.2±0.1	=	+	=		0.1±0.0	=	0.1±0.1	=	+	=	+	=	+	>
18:3/18:1	0.6±0.1	>	0.2±0.1	<	0.4±0.1	<	0.7±0.0	>	0.3±0.1	>		0.2±0.1	=	0.1±0.1	=	0.2±0.1	=	+	=	0.3±0.3	=
18:0/18:4	0.1±0.0	>	+	=	+	=	0.1±0.1	=	0.1±0.0	=		+	=	0.1±0.0	=	+	=	+	=	+	>
18:2/18:1	0.1±0.0	=	0.1±0.0	=	+	<	0.1±0.0	>	0.1±0.1	=		+	=	0.1±0.0	=	0.1±0.0	=	0.1±0.0	=	+	=
20:3/16:0	0.1±0.0	=	0.1±0.0	=	0.1±0.0	=	+	=	0.1±0.0	=		0.1±0.1	=	0.1±0.1	=	0.1±0.0	=	0.1±0.1	=	0.1±0.2	=
16:0/20:3	0.1±0.0	=	0.1±0.0	>	+	=	0.1±0.1	=	+	=		+	=	0.1±0.0	=	0.1±0.0	=	+	=	+	=
18:1/18:1	+	=	0.3±0.2	=	0.4±0.2	>	0.1±0.1	=	0.2±0.1	=		0.2±0.2	=	0.1±0.1	=	0.5±0.0	>	0.1±0.1	=	0.2±0.1	=
20:2/16:0	0.1±0.0	=	0.1±0.1	=	0.1±0.0	<	0.2±0.1	>	0.1±0.0	=		0.1±0.0	=	+	<	0.1±0.1	=	+	=	+	=
18:0/18:2	0.1±0.1	=	0.1±0.1	=	0.1±0.0	=	0.1±0.0	=	0.1±0.0	=		0.1±0.0	=	0.1±0.0	=	0.1±0.1	=	0.1±0.1	=	0.1±0.0	<
18:0/18:1	0.1±0.0	=	0.1±0.1	=	0.1±0.1	=	0.2±0.1	=	0.2±0.2	=		+	>	+	<	0.1±0.0	=	0.1±0.1	=	+	=
16:0/20:0	+	=	+	=	+	=	+	=	+	=		+	=	+	=	+	=	0.1±0.0	=	+	=
20:5/18:4	0.2±0.1	=	0.2±0.1	=	0.2±0.1	>	0.1±0.0	=	0.1±0.0	>		0.4±0.3	=	0.2±0.1	=	0.2±0.2	=	0.1±0.0	=	+	=
20:5/18:3	0.1±0.1	=	+	=	0.1±0.1	=	0.2±0.1	=	+	=		+	=	0.1±0.0	=	0.1±0.0	=	+	=	+	=
20:4/18:4	0.1±0.0	=	0.1±0.1	=	0.1±0.0	=	+	=	+	>		0.1±0.0	=	0.1±0.0	=	+	=	+	=	+	>
20:5/20:5	-	#	-	#	-	#	-	#	-	#		+	=	+	>	-	#	-	<	0.1±0.0	=
20:5/20:4	0.3±0.0	>	+	=	+	=	+	>	-	>		0.1±0.0	>	-	<	+	>	-	#	-	>
20:4/20:4	0.1±0.1	=	+	=	-	<	+	>	-	>		0.1±0.0	=	+	>	-	#	-	<	+	=
16:0/24:0	+	=	+	=	+	=	+	=	0.1±0.1	=		0.1±0.0	=	+	=	0.2±0.1	=	+	=	+	=

PG	*Streblonema corymbiferum*										#	*Streblonema* sp.									
	5 ˚C		10 ˚C		15 ˚C		20 ˚C		25 ˚C	T		5 ˚C		10 ˚C		15 ˚C		20 ˚C		25 ˚C	T
16:1/15:0	+	=	0.1±0.0		0.1±0.1	=	0.1±0.1	=	0.1±0.0	=		0.1±0.0	=	+	<	0.4±0.1	>	0.2±0.1	=	0.1±0.1	=
16:0/15:0	+	=	+	=	0.1±0.0	=	0.1±0.0	=	0.1±0.1	<		+	=	0.2±0.1	<	0.4±0.0	=	0.3±0.3	=	0.5±0.2	<
18:3/14:0	0.2±0.1	=	0.3±0.2	=	0.3±0.1	<	0.5±0.1	=	0.3±0.2	=		0.2±0.0	=	0.2±0.0	=	0.1±0.0	=	0.2±0.1	=	0.2±0.1	=
14:0/18:2	0.1±0.0	=	0.1±0.0	=	0.1±0.1	=	0.2±0.1	=	0.1±0.1	=		0.1±0.0	>	0.1±0.0	<	0.2±0.0	>	0.1±0.0	=	0.1±0.0	=
14:0/18:1	0.2±0.1	=	0.2±0.1	=	0.2±0.2	=	0.2±0.1	=	0.2±0.0	=		0.2±0.1	=	0.2±0.2	=	0.1±0.0	=	0.1±0.1	=	0.1±0.1	=
16:0/16:1	0.1±0.1	=	+	=	+	=	0.1±0.1	=	0.1±0.1	=		0.1±0.1	=	0.1±0.0	<	0.3±0.1	=	0.1±0.1	=	0.2±0.2	=
17:0/16:1	+	=	0.1±0.1	=	0.1±0.0	<	0.1±0.0	=	0.1±0.1	=		+	<	0.1±0.1	=	0.1±0.0	=	0.1±0.0	=	0.1±0.0	<
18:3/16:1	15.4±1.2	=	15.9±1.3	<	19.5±1.8	>	13.3±1.3	=	14.6±4.0	=		11.4±0.8	=	13.0±1.4	=	14.5±0.8	>	10.8±2.5	<	16.5±2.8	<
18:3/16:0	13.8±1.4	<	16.9±0.7	<	22.1±1.0	=	24.0±2.1	<	30.8±1.8	<		12.2±1.7	<	16.0±2.3	=	18.1±2.7	<	27.1±0.4	=	28.8±3.2	<
18:1/16:1	0.1±0.0	=	0.1±0.0	=	0.1±0.0	=	0.2±0.1	=	0.3±0.1	<		0.2±0.2	=	0.2±0.1	<	0.9±0.4	>	0.4±0.1	=	0.4±0.0	=
16:0/18:2, 18:2/16:0	8.6±1.6	>	5.2±0.4	=	5.4±0.4	<	6.5±0.8	=	7.1±1.0	>		8.6±1.3	=	7.5±1.0	=	6.0±1.1	<	10.9±1.4	<	14.3±2.4	<
16:0/18:1	9.5±0.6	<	11.6±1.1	=	10.3±0.8	>	8.7±1.0	=	7.6±0.7	>		12.3±1.0	<	18.0±1.2	<	26.6±1.3	>	13.8±0.4	=	14.0±0.8	<
17:0/18:3	+	=	+	<	0.1±0.0	=	0.1±0.1	=	0.1±0.0	=		+	=	+	<	0.2±0.0	>	+	=	0.1±0.0	=
17:0/18:1	0.2±0.1	=	0.4±0.2	>	0.1±0.1	=	0.3±0.2	=	0.3±0.3	=		0.1±0.1	=	0.2±0.1	=	0.2±0.1	=	0.2±0.1	=	0.1±0.1	=
18:4/18:4	0.1±0.1	=	0.1±0.0	>	+	=	0.1±0.0	=	0.1±0.0	=		0.5±0.2	>	0.1±0.1	=	0.1±0.0	=	0.1±0.0	=	0.1±0.1	>
18:3/18:4	10.0±1.4	>	5.5±0.2	>	2.6±0.4	>	1.8±0.4	>	0.8±0.4	>		19.7±1.8	>	10.6±1.2	>	4.3±0.2	>	2.3±0.4	>	0.9±0.5	>
18:3/18:3	23.8±1.0	=	22.8±0.8	>	19.1±1.0	=	19.1±1.7	>	10.6±1.2	>		21.4±1.2	>	18.8±1.7	>	14.4±0.4	=	13.6±1.5	>	7.1±0.7	>
16:1/20:5	0.1±0.0	=	0.1±0.1	=	0.1±0.1	=	+	=	+	=		0.1±0.0	=	0.1±0.0	=	0.2±0.1	=	0.1±0.1	=	0.2±0.2	=
18:3/18:2	7.7±1.1	=	8.1±0.3	>	6.5±0.2	=	7.5±1.7	=	6.2±1.6	=		6.5±0.3	=	6.3±0.9	>	3.7±0.2	<	7.4±1.5	=	6.3±0.5	=
18:2/18:3	0.2±0.3	=	0.1±0.1	=	+	<	0.5±0.1	>	0.1±0.1	=		+	<	0.3±0.1	>	0.1±0.0	<	0.6±0.1	=	1.0±0.8	=
20:5/16:0	0.1±0.1	=	+	=	+	=	0.1±0.0	=	0.1±0.0	=		+	=	+	<	0.1±0.1	>	+	=	0.1±0.0	=
16:0/20:5	0.4±0.1	=	0.3±0.2	=	0.4±0.1	>	0.1±0.0	=	0.1±0.1	>		0.3±0.1	=	0.4±0.2	=	0.2±0.0	=	0.3±0.3	=	+	>
18:2/18:2	0.9±0.4	=	0.8±0.3	>	0.3±0.1	<	0.8±0.1	=	0.6±0.3	=		1.5±0.2	=	1.5±0.3	>	0.6±0.1	<	3.0±1.2	=	2.7±0.7	<
18:3/18:1	3.3±0.5	<	5.1±0.9	=	5.5±0.5	<	7.6±0.7	>	5.6±0.2	<		0.4±0.1	<	1.3±0.2	>	1.0±0.0	=	1.6±0.5	=	0.9±0.4	=
18:1/18:3	+	=	-	=	+	=	0.3±0.2	=	0.2±0.2	=		+	=	0.1±0.0	=	0.1±0.0	=	+	<	0.2±0.0	<
20:4/16:0	0.1±0.0	=	0.1±0.1	=	0.1±0.1	=	0.1±0.0	=	0.1±0.0	=		0.1±0.0	=	0.1±0.1	=	0.1±0.1	=	+	=	0.1±0.1	=
16:0/20:4	0.1±0.1	=	0.1±0.0	=	0.1±0.1	=	+	=	0.1±0.1	=		0.1±0.1	=	0.1±0.1	<	0.3±0.0	>	0.1±0.0	=	0.1±0.0	=
18:2/18:1	1.7±0.2	=	2.1±0.4	=	2.0±0.3	<	2.9±0.3	<	8.2±2.0	<		0.5±0.0	=	0.9±0.4	=	0.7±0.1	<	1.2±0.2	=	0.6±0.5	=
18:1/18:2	0.2±0.1	=	0.3±0.2	=	0.2±0.2	=	0.2±0.2	=	0.2±0.2	=		0.2±0.2	=	0.3±0.1	=	0.3±0.0	=	0.3±0.2	=	0.2±0.1	=
20:3/16:0	0.1±0.1	=	+	=	+	=	0.1±0.1	=	0.1±0.1	=		+	=	0.1±0.0	<	0.1±0.0	=	0.1±0.1	=	-	>
18:3/18:0	0.1±0.1	=	+	=	0.1±0.0	=	+	=	0.1±0.1	=		+	=	0.1±0.0	#	-	#	-	<	+	=
18:0/18:3	0.1±0.1	=	0.1±0.0	=	0.1±0.0	=	0.1±0.0	>	-	>		+	>	-	#	-	#	+	>	-	>
18:1/18:1	1.3±0.2	<	1.9±0.4	<	2.9±0.2	=	3.0±0.6	<	4.0±0.1	<		1.1±0.1	=	1.8±0.8	<	3.9±0.7	=	4.0±0.8	=	4.0±1.2	<
18:0/18:2	0.2±0.0	=	0.2±0.1	=	0.2±0.1	>	0.1±0.0	=	+	>		0.2±0.1	=	0.2±0.2	=	0.4±0.1	=	0.2±0.2	=	0.1±0.0	=
18:0/18:1	0.7±0.1	=	0.9±0.3	=	1.0±0.2	=	0.7±0.4	=	0.4±0.1	=		0.2±0.1	=	0.2±0.1	<	0.5±0.2	>	0.3±0.1	=	0.3±0.2	=
18:4/20:5	+	=	+	=	+	<	0.1±0.0	>	+	=		0.1±0.0	=	+	=	0.1±0.0	=	0.1±0.0	>	-	>
20:5/18:3	0.6±0.3	>	0.2±0.1	=	0.3±0.2	=	0.3±0.2	=	0.2±0.1	>		1.0±0.1	>	0.5±0.3	=	0.3±0.1	=	0.2±0.2	=	0.1±0.1	>
20:5/18:2	0.1±0.0	=	0.1±0.1	=	+	=	0.1±0.0	=	0.1±0.0	=		0.2±0.0	=	0.2±0.1	=	0.2±0.1	=	0.1±0.1	=	0.1±0.0	>
20:5/18:2	+	=	+	=	+	=	0.1±0.1	=	0.1±0.1	=		0.1±0.1	=	0.2±0.1	#	-	#	+	=	0.1±0.0	=
18:2/20:5	0.1±0.0	=	+	=	0.1±0.0	=	+	<	0.1±0.0	=		+	=	0.1±0.0	=	0.1±0.0	>	-	<	0.1±0.0	=
18:1/20:5	+	=	+	=	+	=	0.1±0.1	=	0.1±0.1	=		+	=	+	<	0.1±0.0	=	+	=	0.1±0.0	=
20:5/20:5	+	=	+	=	+	>	-	<	0.1±0.0	=		0.1±0.0	=	+	<	0.3±0.0	>	0.1±0.0	=	0.2±0.0	=
20:4/20:5	+	=	+	=	0.1±0.0	=	0.1±0.1	=	0.1±0.0	=		0.1±0.1	=	0.2±0.2	=	0.2±0.0	>	+	=	0.1±0.0	=

PE	*Streblonema corymbiferum*											*Streblonema* sp.									
	5 ˚C		10 ˚C		15 ˚C		20 ˚C		25 ˚C	T	#	5 ˚C		10 ˚C		15 ˚C		20 ˚C		25 ˚C	T
16:1/16:1	0.1±0.1	=	+		0.1±0.0	<	0.2±0.1	=	0.1±0.1	=		0.1±0.0	<	0.2±0.1	=	0.3±0.2	=	0.3±0.1	>	0.1±0.1	=
16:0/16:1	0.1±0.1	=	+	=	0.1±0.1	=	+	<	0.1±0.1	=		0.1±0.0	=	0.1±0.1	=	0.1±0.0	=	0.1±0.1	=	0.2±0.2	=
14:0/20:5	1.3±0.6	=	1.7±0.6	=	2.0±0.4	=	2.5±0.4	<	3.5±0.1	<		0.6±0.4	=	0.4±0.2	=	0.5±0.2	=	0.5±0.1	=	0.4±0.2	=
14:0/20:4	0.8±0.3	=	1.5±0.8	<	3.9±0.3	=	4.5±0.6	<	6.6±0.7	<		0.4±0.2	=	0.2±0.1	<	0.4±0.1	=	0.9±0.4	=	1.5±0.5	<
18:1/16:1	0.2±0.2	=	0.1±0.1	=	0.1±0.1	=	0.1±0.0	=	0.1±0.1	=		0.5±0.2	=	0.8±0.2	=	0.7±0.1	=	0.9±0.5	=	0.9±0.0	=
16:0/18:1	+	=	0.1±0.0	=	0.1±0.0	=	0.2±0.2	=	0.2±0.1	=		0.1±0.0	<	0.4±0.1	=	0.4±0.2	=	0.4±0.2	=	0.7±0.1	<
18:0/16:0	+	=	+	=	+	=	+	=	+	=		+	=	0.1±0.1	=	0.1±0.1	=	+	=	0.2±0.3	=
20:5/15:0	+	=	0.1±0.0	=	0.1±0.0	=	0.1±0.1	=	0.1±0.1	=		+	=	0.1±0.0	=	0.1±0.0	=	0.1±0.0	=	0.2±0.3	=
20:4/15:0	+	=	+	=	+	=	+	=	0.1±0.0	=		+	=	0.1±0.1	=	0.1±0.1	=	0.1±0.0	=	0.2±0.2	=
18:3/18:4	+	=	+	=	+	=	0.1±0.1	=	0.1±0.1	=		+	=	+	=	+	=	0.1±0.0	=	0.1±0.1	=
18:4/18:3	+	=	+	=	+	=	0.1±0.0	=	0.1±0.1	=		+	=	+	=	0.1±0.1	=	0.1±0.0	=	0.1±0.1	=
16:0/20:5	4.1±0.6	=	3.8±1.0	=	4.7±0.5	=	3.8±0.8	=	4.5±0.6	=		2.5±0.5	=	2.0±0.5	<	4.3±0.1	>	1.6±0.9	=	0.6±0.5	>
18:2/18:2	0.3±0.4	=	0.1±0.1	=	0.1±0.0	=	+	=	+	>		0.1±0.0	<	0.1±0.1	=	0.1±0.1	=	0.1±0.1	=	0.1±0.1	<
16:0/20:4	2.9±0.5	<	4.2±0.4	=	4.9±0.5	<	6.2±1.0	<	8.4±1.2	<		2.3±0.6	<	3.8±0.9	<	6.5±1.1	<	10.0±1.2	<	12.8±0.8	<
18:2/18:1	0.2±0.1	=	0.4±0.1	=	0.1±0.1	=	0.1±0.0	>	+	>		0.2±0.2	=	0.2±0.2	=	0.4±0.3	=	0.2±0.1	=	0.6±0.7	=
18:1/18:1	0.2±0.2	<	0.8±0.4	=	0.8±0.2	=	0.8±0.2	=	0.8±0.1	<		0.3±0.1	<	1.0±0.2	=	1.0±0.4	=	0.6±0.2	=	0.8±0.2	<
20:5/18:4	0.2±0.1	=	0.2±0.1	=	0.3±0.1	=	0.7±0.3	=	0.7±0.3	=		0.2±0.1	=	0.1±0.0	=	0.1±0.1	=	0.2±0.1	=	0.5±0.3	=
20:5/18:3	0.7±0.4	=	0.4±0.4	=	0.1±0.1	=	0.3±0.2	=	0.6±0.3	=		0.6±0.3	=	0.4±0.4	=	0.1±0.1	=	0.1±0.1	=	0.1±0.0	>
18:3/20:5	0.8±0.5	=	0.6±0.5	=	0.3±0.1	=	0.4±0.1	<	0.8±0.3	=		0.2±0.1	=	0.2±0.0	>	0.1±0.0	=	0.2±0.2	=	0.2±0.1	=
18:3/20:4	1.7±0.9	=	1.0±0.2	>	0.6±0.1	<	1.1±0.3	=	1.3±0.6	=		0.5±0.3	=	0.6±0.1	>	0.3±0.2	=	0.4±0.2	=	0.8±0.4	=
18:2/20:5	0.2±0.2	=	0.1±0.0	=	0.1±0.1	=	0.1±0.1	=	0.1±0.1	=		0.1±0.0	=	0.2±0.1	=	0.1±0.0	=	0.1±0.1	=	0.1±0.1	=
18:2/20:4	0.5±0.3	=	0.4±0.3	=	0.2±0.3	=	0.3±0.1	<	0.7±0.3	=		0.3±0.3	=	0.2±0.1	=	0.2±0.1	=	0.2±0.1	=	0.3±0.2	=
18:1/20:5	0.8±0.1	>	0.5±0.1	>	0.3±0.1	<	0.5±0.1	=	0.5±0.1	>		0.4±0.2	=	0.5±0.2	=	0.2±0.1	=	0.5±0.5	=	0.8±0.3	=
18:1/20:5	0.2±0.2	=	0.3±0.0	<	0.4±0.0	=	0.4±0.1	=	0.3±0.1	=		0.3±0.2	=	0.3±0.2	=	0.2±0.1	=	0.2±0.2	=	0.5±0.4	=
18:1/20:4	0.4±0.2	=	0.5±0.3	=	0.5±0.1	=	0.5±0.2	<	1.8±0.2	<		0.2±0.0	=	0.4±0.2	>	0.2±0.1	<	0.9±0.2	=	1.2±0.8	=
18:0/20:5	1.1±0.5	=	1.0±0.8	=	1.3±1.0	=	0.9±0.2	=	0.8±0.3	=		0.5±0.2	=	0.9±0.4	=	1.6±0.5	>	0.8±0.1	=	0.5±0.2	=
18:0/20:4	1.2±0.1	<	2.3±0.5	=	2.7±0.4	=	2.7±0.8	=	2.5±0.3	<		1.4±0.1	<	3.0±0.9	<	4.4±0.5	<	6.0±0.7	=	4.1±1.6	<
20:5/20:5	12.9±1.5	>	9.5±1.0	>	7.6±0.9	>	3.5±1.0	=	3.1±0.1	>		20.4±2.2	>	12.1±0.8	>	7.7±0.7	>	1.4±0.2	=	1.8±1.0	>
20:4/20:5	36.7±2.1	>	29.7±1.0	>	26.0±1.6	>	20.0±1.6	=	17.9±1.7	>		42.5±1.8	>	37.0±1.3	>	28.6±0.3	>	24.1±1.7	>	17.2±0.6	>
20:5/20:3	0.3±0.2	=	0.3±0.2	=	0.2±0.1	=	0.1±0.0	=	0.1±0.1	>		0.8±0.3	>	0.2±0.1	=	0.1±0.0	=	0.1±0.1	=	0.2±0.1	>
20:3/20:5	0.2±0.0	<	0.4±0.0	>	0.2±0.0	=	0.2±0.1	=	0.1±0.1	=		0.4±0.1	=	0.2±0.1	=	0.2±0.0	=	0.1±0.1	<	0.6±0.1	=
20:4/20:4	26.4±1.6	<	31.2±1.7	=	30.3±1.7	<	35.4±2.4	=	32.6±1.3	<		22.3±3.0	<	30.9±1.0	<	35.0±1.1	<	40.7±2.8	=	43.3±2.5	<
20:3/20:4	0.2±0.2	=	0.1±0.0	=	0.2±0.0	=	0.1±0.0	=	0.1±0.1	>		0.4±0.2	>	0.2±0.1	=	0.2±0.3	=	0.2±0.3	=	0.3±0.4	=
20:4/20:2	0.1±0.0	=	+	=	+	=	0.1±0.0	=	+	=		0.1±0.0	=	0.1±0.1	=	0.1±0.1	=	0.1±0.0	=	0.1±0.0	=
20:1/20:5	0.7±0.2	=	0.7±0.1	=	0.6±0.0	=	0.6±0.3	>	0.3±0.0	>		0.1±0.0	=	0.1±0.0	>	+	=	0.1±0.0	=	+	>
20:1/20:4,<br>20:0/20:5	2.4±0.3	<	3.3±0.3	<	4.0±0.3	=	3.9±0.4	>	2.7±0.1	=		0.3±0.1	<	0.6±0.1	<	1.4±0.3	=	1.0±0.3	=	0.8±0.5	=
20:0/20:4	1.5±0.6	<	2.8±0.1	<	3.8±0.6	<	5.2±0.2	>	3.7±0.1	<		0.3±0.1	<	1.2±0.4	<	2.1±0.5	<	3.9±0.1	=	3.6±0.9	<
20:4/22:1	0.1±0.0	>	+	=	0.1±0.0	=	0.1±0.0	=	0.1±0.0	=		+	=	+	<	0.2±0.1	=	0.3±0.4	=	0.2±0.2	=
22:0/20:5	0.3±0.1	<	0.8±0.3	<	1.5±0.2	=	1.4±0.5	=	0.9±0.6	=		0.1±0.0	=	0.2±0.1	=	0.3±0.2	=	0.3±0.3	=	0.5±0.0	<
22:0/20:4	0.2±0.1	<	1.0±0.2	<	1.7±0.1	=	2.5±0.7	<	3.3±0.1	<		0.1±0.0	<	0.4±0.1	<	0.8±0.0	<	1.6±0.4	=	2.2±0.4	<
24:0/20:5	+	=	+	=	0.1±0.1	=	0.1±0.1	=	0.1±0.1	=		0.3±0.2	=	0.5±0.1	=	0.5±0.4	=	0.2±0.1	=	0.2±0.2	=
24:0/20:4	+	=	+	=	0.1±0.1	=	0.1±0.1	=	0.2±0.1	=		0.1±0.1	<	0.4±0.2	=	0.4±0.4	=	0.5±0.6	=	1.0±0.3	<

PC	*Streblonema corymbiferum*											*Streblonema* sp.									
	5 ˚C		10 ˚C		15 ˚C		20 ˚C		25 ˚C	T	#	5 ˚C		10 ˚C		15 ˚C		20 ˚C		25 ˚C	T
14:0/14:1	+	=	+		-	<	+	=	+	=		-	#ДЕЛ/0!	-	#	-	<	+	>	-	#
14:0/14:0	+	=	+	<	0.1±0.0	=	0.1±0.1	=	0.1±0.1	=		+	<	+	=	+	=	0.1±0.1	=	0.1±0.0	<
14:0/16:1	+	=	+	=	+	=	+	=	+	=		+	=	+	=	+	=	+	=	+	=
14:0/16:1	+	=	+	=	+	=	+	=	+	=		+	=	+	=	+	=	+	=	+	=
14:0/16:0	+	=	+	=	+	=	+	=	0.1±0.1	=		+	=	+	<	+	=	+	<	0.1±0.1	<
18:4/14:1	+	=	+	=	+	=	+	=	+	=		+	>	-	#	-	#	-	#	-	>
14:0/18:4	0.2±0.1	=	0.2±0.1	=	0.1±0.1	=	0.1±0.1	=	0.1±0.1	=		0.2±0.1	=	0.1±0.0	>	+	=	+	=	0.1±0.0	=
14:0/18:3	2.7±0.1	=	3.0±0.2	=	2.8±0.1	=	3.0±0.2	<	3.5±0.3	<		2.0±0.1	>	1.1±0.2	=	1.4±0.1	=	1.2±0.2	>	0.4±0.1	>
14:0/18:3	0.2±0.2	=	0.4±0.2	=	0.2±0.1	=	0.2±0.2	=	0.4±0.2	=		0.3±0.2	<	0.8±0.2	>	0.1±0.0	<	0.4±0.2	<	1.5±0.5	<
14:0/18:2	2.1±0.5	=	2.4±0.1	<	3.0±0.2	=	2.9±0.4	<	4.1±0.5	<		1.5±0.1	=	1.8±0.2	<	2.3±0.0	<	2.7±0.3	<	3.2±0.2	<
14:0/18:2	0.5±0.3	=	0.3±0.1	=	0.1±0.0	<	0.5±0.3	=	0.7±0.2	=		0.3±0.1	=	0.3±0.3	=	0.1±0.1	<	0.3±0.1	<	0.5±0.0	<
14:0/18:1	1.2±0.2	<	2.4±0.3	<	3.0±0.3	<	6.0±0.3	<	9.5±0.8	<		0.2±0.2	<	0.6±0.2	=	0.5±0.1	<	1.3±0.1	<	2.6±0.0	<
16:0/16:0	+	=	+	=	+	=	+	=	+	=		+	<	+	>	+	=	+	<	0.1±0.0	<
17:3/16:1	+	=	+	=	+	=	+	=	+	=		+	=	+	<	+	=	+	=	+	=
15:0/18:2	+	=	+	=	+	=	+	=	+	=		+	=	+	=	+	=	+	=	+	=
15:0/18:1	+	=	+	=	0.1±0.0	=	0.1±0.1	=	0.1±0.0	=		+	=	+	=	+	=	+	<	0.1±0.0	<
14:0/20:5	17.2±0.6	<	18.7±0.2	=	19.3±0.5	>	16.1±1.4	=	15.1±1.7	=		17.2±1.1	>	11.9±1.0	>	10.1±0.4	>	7.5±0.6	=	6.7±0.8	>
14:0/20:4	0.4±0.4	=	0.5±0.1	>	0.2±0.1	<	1.7±0.3	>	0.6±0.2	=		0.5±0.1	=	0.5±0.1	>	0.2±0.1	<	0.5±0.3	=	0.3±0.1	=
14:0/20:4	6.0±0.7	<	7.8±0.1	<	9.5±0.6	=	10.7±1.5	=	9.2±1.1	<		3.7±0.3	=	4.6±0.8	=	4.7±0.3	<	5.9±0.2	=	7.1±1.0	<
14:0/20:3	0.2±0.1	=	0.2±0.0	=	0.2±0.0	=	0.2±0.1	=	0.4±0.2	=		0.1±0.1	=	+	<	0.3±0.1	>	0.1±0.0	=	0.1±0.1	=
16:0/18:3	2.5±0.1	=	2.7±0.1	>	2.2±0.1	<	2.6±0.3	=	2.2±0.2	=		2.4±0.2	<	3.0±0.1	>	2.3±0.3	<	3.3±0.3	=	3.3±1.0	=
18:0/16:3	0.3±0.3	=	0.3±0.1	=	0.3±0.1	<	0.5±0.0	=	0.5±0.3	=		0.1±0.1	=	0.2±0.1	=	0.1±0.1	=	0.2±0.2	=	0.4±0.3	=
16:0/18:2	2.1±0.1	=	1.9±0.2	=	1.8±0.2	=	1.8±0.1	<	2.6±0.2	<		2.6±0.4	<	3.9±0.6	=	4.3±0.5	<	6.0±0.3	<	6.7±0.4	<
16:0/18:1	1.0±0.2	<	1.8±0.2	=	1.8±0.2	<	3.2±0.2	<	5.5±0.2	<		0.3±0.1	<	0.6±0.1	<	0.9±0.0	<	2.9±0.2	<	4.6±0.7	<
18:4/17:2	0.1±0.1	=	0.3±0.1	=	0.2±0.1	=	0.1±0.1	=	0.1±0.0	=		0.3±0.2	=	0.1±0.1	=	0.1±0.0	>	0.1±0.0	=	0.1±0.0	>
17:2/18:3	+	=	+	=	+	<	0.1±0.0	=	0.1±0.0	<		+	=	+	=	+	=	+	=	0.1±0.0	=
15:0/20:5	0.2±0.1	=	0.3±0.1	=	0.2±0.1	=	0.3±0.1	=	0.3±0.2	=		0.9±0.2	>	0.3±0.1	=	0.2±0.1	=	0.2±0.2	=	0.1±0.1	>
17:1/18:3	+	=	+	=	+	=	0.1±0.1	=	+	=		+	=	0.1±0.0	=	0.1±0.0	=	0.1±0.0	=	0.2±0.1	<
15:0/20:4	0.2±0.2	=	0.1±0.0	=	0.2±0.1	=	0.1±0.1	=	0.1±0.0	>		+	=	0.1±0.0	=	0.1±0.0	=	0.1±0.1	=	0.2±0.1	<
15:0/20:4	0.1±0.0	=	0.1±0.0	<	0.2±0.0	=	0.2±0.1	=	0.2±0.2	=		+	=	0.1±0.1	=	0.1±0.0	=	0.1±0.0	=	0.2±0.1	=
17:1/18:2	+	<	+	=	+	=	+	=	+	=		+	=	+	=	+	=	+	=	+	=
17:1/18:2	+	=	+	=	+	=	0.1±0.1	=	+	=		+	=	+	=	+	=	+	=	0.1±0.1	=
17:0/18:2	+	=	+	=	+	=	+	<	0.1±0.0	<		+	=	0.1±0.1	=	+	=	0.1±0.1	=	0.1±0.1	=
16:0/19:1	+	=	+	=	+	=	+	=	+	=		+	=	+	=	+	=	+	=	+	=
18:3/18:3	0.1±0.0	=	0.1±0.0	=	0.1±0.0	=	0.1±0.0	=	0.1±0.0	=		0.2±0.2	=	0.1±0.0	=	0.1±0.0	=	0.1±0.0	=	0.1±0.1	=
20:5/16:1	0.1±0.0	=	0.1±0.1	=	+	=	+	=	0.1±0.0	=		0.1±0.0	=	0.1±0.0	=	0.1±0.0	=	0.1±0.0	=	0.1±0.1	=
16:1/20:5	+	=	+	=	+	=	+	<	0.1±0.0	=		+	=	+	=	+	=	0.1±0.1	=	0.1±0.2	=
18:3/18:2	0.1±0.0	=	0.1±0.0	=	+	<	0.1±0.0	=	0.1±0.1	=		0.1±0.1	=	0.1±0.0	=	0.1±0.1	=	+	=	0.1±0.0	=
20:4/16:1	0.1±0.0	=	0.1±0.0	=	+	=	+	=	+	=		0.1±0.1	>	0.1±0.0	=	0.1±0.1	=	0.2±0.1	=	0.1±0.1	=
16:0/20:5	15.0±0.4	>	11.1±0.2	>	8.7±0.2	>	5.8±1.3	=	6.3±0.8	>		18.7±0.1	=	17.6±2.0	=	16.2±1.0	>	11.7±1.5	>	8.5±0.6	>
16:0/20:4	0.1±0.0	=	0.1±0.1	=	0.2±0.1	=	0.1±0.1	<	0.8±0.2	<		0.3±0.2	=	0.2±0.2	=	0.4±0.1	=	0.5±0.1	=	0.5±0.4	=
16:0/20:4	4.9±0.4	=	4.2±0.3	=	4.3±0.5	=	4.3±0.3	>	2.8±0.5	>		4.3±0.4	<	6.4±0.1	<	7.3±0.4	<	9.2±0.1	=	9.2±0.1	<
18:2/18:1	0.2±0.0	=	0.2±0.1	=	0.2±0.1	=	0.2±0.0	=	0.2±0.1	=		0.2±0.0	=	0.2±0.1	=	0.3±0.2	=	0.2±0.2	=	0.3±0.2	=
18:1/18:2	0.2±0.1	=	0.2±0.0	=	0.2±0.0	=	0.2±0.1	>	0.1±0.0	=		0.1±0.1	<	0.4±0.1	>	0.2±0.1	=	0.4±0.3	=	0.4±0.2	<
16:0/20:3	0.2±0.1	<	0.6±0.1	=	0.6±0.1	=	0.4±0.1	>	0.1±0.1	=		0.5±0.1	=	0.5±0.2	=	0.4±0.1	<	1.0±0.3	=	1.3±0.2	<
16:0/20:3	0.2±0.0	=	0.3±0.2	=	0.3±0.2	=	0.2±0.1	=	0.1±0.1	=		0.1±0.1	=	0.2±0.2	=	0.2±0.3	=	0.5±0.2	=	0.4±0.2	=
18:1/18:1	0.1±0.0	=	0.1±0.0	<	0.2±0.0	>	0.1±0.0	<	0.2±0.0	<		0.1±0.1	=	0.1±0.1	=	+	<	0.1±0.0	=	0.1±0.0	=
18:1/18:1	0.1±0.0	<	0.1±0.0	=	0.1±0.0	=	0.2±0.1	=	0.2±0.1	=		0.1±0.0	<	0.2±0.0	>	0.1±0.0	=	0.2±0.1	=	0.1±0.0	=
18:0/18:2	+	=	0.1±0.0	<	0.1±0.0	=	0.1±0.1	=	0.1±0.0	=		0.1±0.0	=	0.1±0.0	=	0.1±0.1	=	0.3±0.2	=	0.2±0.0	<
18:0/18:1	+	<	+	=	+	=	0.1±0.0	=	0.1±0.1	=		+	=	+	=	+	=	+	=	0.1±0.1	=
20:5/18:4	+	=	0.1±0.1	=	0.1±0.0	=	0.1±0.1	=	+	=		0.2±0.0	>	0.1±0.0	=	0.1±0.1	=	+	=	0.1±0.1	>
20:5/18:4	0.1±0.1	=	0.1±0.1	=	+	=	+	=	+	>		0.2±0.1	=	0.2±0.2	=	+	=	+	=	+	>
20:5/18:3	2.4±0.3	=	2.1±0.2	=	1.7±0.2	>	1.4±0.2	=	1.4±0.2	>		3.4±0.2	=	3.3±0.6	>	2.3±0.5	=	1.8±0.3	=	1.5±0.2	>
20:5/18:2	2.1±0.2	<	2.7±0.3	=	2.7±0.2	>	1.7±0.2	=	1.7±0.3	>		2.8±0.3	<	3.5±0.3	<	4.8±0.3	>	3.5±0.3	>	2.1±0.2	>
20:4/18:3	1.6±0.4	>	0.7±0.1	>	0.4±0.1	<	0.9±0.2	=	1.1±0.3	>		1.5±0.6	=	1.3±0.7	>	0.3±0.0	<	1.3±0.4	<	2.2±0.2	=
20:3/18:3	+	=	+	=	+	=	+	=	+	=		+	=	+	=	0.1±0.1	=	+	<	+	=
20:4/18:2	1.6±0.3	=	1.4±0.2	>	0.9±0.1	<	1.4±0.2	=	1.7±0.7	=		0.9±0.1	<	1.8±0.1	=	2.0±0.4	=	2.7±0.6	=	3.5±0.2	<
20:5/18:1	1.0±0.1	<	1.3±0.2	<	2.0±0.1	=	2.0±0.4	<	2.8±0.3	<		1.1±0.3	=	1.4±0.3	=	1.8±0.3	<	2.6±0.5	>	1.9±0.1	<
18:2/20:3	0.1±0.0	=	0.1±0.1	=	+	=	0.1±0.0	=	0.1±0.1	=		0.2±0.1	=	0.1±0.1	<	0.2±0.0	=	0.1±0.0	=	0.2±0.1	=
20:4/18:1	0.9±0.1	<	1.5±0.1	<	1.9±0.2	<	4.0±0.4	=	4.6±0.3	<		0.2±0.0	<	0.4±0.1	<	0.9±0.1	<	1.9±0.2	<	2.9±0.1	<
18:0/20:5	0.4±0.2	=	0.6±0.1	>	0.4±0.1	=	0.6±0.2	=	0.4±0.2	=		0.4±0.1	<	0.6±0.0	<	1.0±0.0	=	0.8±0.2	=	0.6±0.1	=
18:0/20:4	0.1±0.0	=	0.1±0.1	=	0.1±0.1	=	0.1±0.0	<	0.1±0.0	<		+	=	+	<	0.3±0.1	>	0.2±0.0	=	0.3±0.2	=
18:1/20:1	+	=	+	=	+	=	+	=	+	=		+	=	+	=	+	=	+	=	+	<
18:2/20:0	+	=	+	=	+	=	+	=	+	=		+	=	+	=	+	=	+	=	+	=

16:0/22:1	+	=	+	=	+	=	+	=	+	=	+	=	+	=	+	=	+	=	+	=
18:1/20:0	+	=	+	=	+	=	+	=	+	=	+	=	+	=	+	>	-	<	+	=
20:4/19:2	+	=	+	=	+	=	+	=	+	=	+	<	+	=	0.1±0.0	=	0.1±0.0	<	0.1±0.0	<
20:4/19:1	+	=	+	=	+	=	+	=	+	=	+	<	+	=	0.1±0.0	=	+	=	+	=
20:5/20:5	11.4±0.9	>	8.8±0.6	=	8.1±0.7	>	4.2±1.0	=	4.4±1.4	>	16.0±1.6	>	11.3±0.3	>	8.4±0.5	>	4.5±0.2	>	2.6±0.5	>
20:5/20:4	0.1±0.1	=	+	=	+	<	0.1±0.0	>	+	>	0.8±0.3	>	+	=	0.2±0.3	=	0.3±0.2	=	0.1±0.1	>
20:4/20:5	14.0±0.4	=	13.4±1.5	=	13.9±0.9	>	10.9±0.6	>	7.9±0.5	>	10.8±0.5	<	13.9±1.4	=	15.7±1.7	>	11.7±0.9	>	9.8±0.1	>
20:5/20:3	0.2±0.1	=	0.3±0.2	=	0.1±0.0	=	0.4±0.2	=	0.1±0.1	=	0.1±0.1	=	0.1±0.1	=	0.2±0.1	=	0.2±0.1	=	0.2±0.1	<
20:3/20:5	0.2±0.1	=	0.3±0.0	=	0.2±0.1	<	0.9±0.1	>	0.3±0.2	=	0.5±0.3	=	0.3±0.2	=	0.5±0.2	=	0.7±0.4	=	0.4±0.2	=
20:4/20:4	3.9±0.4	<	4.5±0.0	<	5.2±0.1	=	6.0±1.1	=	4.2±1.1	=	2.5±0.4	<	3.6±0.3	<	6.0±0.4	<	7.8±1.1	=	9.6±1.7	<
20:4/20:3	0.7±0.1	<	0.9±0.1	=	1.0±0.1	=	1.4±0.4	=	1.0±0.1	=	0.4±0.1	<	0.7±0.0	=	0.7±0.1	<	1.2±0.2	=	1.1±0.2	<
20:4/20:2	+	=	+	=	+	=	+	=	+	=	+	=	0.1±0.0	=	0.1±0.0	=	+	=	+	=
20:4/20:2	0.1±0.1	=	0.1±0.0	=	0.1±0.0	=	0.1±0.0	=	0.1±0.1	=	+	=	0.1±0.0	=	+	=	0.1±0.0	=	0.1±0.0	<
20:5/20:1	+	=	+	=	+	=	+	=	+	=	+	=	+	=	+	=	+	=	+	=
20:4/20:1	+	=	+	=	+	=	+	=	+	=	+	=	+	>	-	<	+	=	+	=
20:1/20:4	+	=	+	=	+	=	+	=	+	=	+	=	+	>	-	=	+	=	+	=
20:4/20:1	+	=	+	=	0.1±0.1	=	+	<	0.1±0.0	<	+	=	+	=	+	=	+	=	+	=
20:0/20:4	+	=	+	=	+	=	+	=	+	=	+	=	+	=	+	=	+	<	+	<
20:5/22:6	0.1±0.0	=	+	=	+	=	+	=	+	>	+	=	0.1±0.0	=	0.1±0.1	=	+	=	+	=
20:4/22:4	+	=	+	=	+	=	+	=	+	=	+	=	+	=	+	=	+	=	+	=

PI	*Streblonema corymbiferum*											*Streblonema* sp.									
	5 ˚C		10 ˚C		15 ˚C		20 ˚C		25 ˚C	T	#	5 ˚C		10 ˚C		15 ˚C		20 ˚C		25 ˚C	T
14:0/18:1, 16:0/16:1	2.9±1.1	=	4.4±1.7		5.2±0.6	=	4.9±1.0	=	6.4±1.2	<		1.7±0.3	<	4.6±1.0	=	3.6±1.2	=	4.6±1.4	=	3.6±2.6	=
16:0/18:4	0.8±0.7	=	1.2±0.8	=	1.4±0.9	=	0.7±0.4	=	0.7±0.1	=		0.3±0.1	=	0.9±1.1	=	1.3±0.0	>	-	<	0.4±0.0	=
16:0/18:3	8.3±1.2	=	5.5±2.9	=	6.1±1.7	=	4.4±3.0	=	5.1±0.9	=		6.9±1.4	=	5.0±0.9	=	4.4±2.3	=	2.6±1.0	>	0.8±0.3	>
16:0/18:2	13.3±0.1	>	9.6±1.4	=	9.4±0.8	=	8.5±1.8	=	8.6±1.9	>		14.6±2.6	=	11.9±2.2	=	12.2±2.7	<	16.3±0.9	=	14.9±3.0	=
16:0/18:1	69.7±1.5	<	73.7±2.8	>	68.1±3.2	=	69.5±4.1	=	67.2±1.7	=		73.5±3.3	=	75.0±1.3	=	77.3±4.0	=	74.4±1.4	=	77.2±2.2	=
16:0/18:0	1.0±0.2	=	1.6±0.5	<	4.5±0.8	=	5.8±1.6	=	6.5±1.9	<		0.3±0.1	=	0.8±0.8	=	0.4±0.4	=	0.5±0.3	=	0.5±0.2	=
16:0/20:5	1.4±0.7	=	0.7±0.3	<	1.3±0.4	=	0.8±0.5	=	1.0±0.5	=		1.6±0.9	=	0.8±0.3	=	0.6±0.3	=	0.8±0.3	=	0.6±0.1	=
18:3/18:0	0.2±0.1	=	0.3±0.2	=	0.3±0.2	=	0.6±0.2	>	0.3±0.1	=		0.4±0.2	=	0.4±0.2	=	0.7±0.1	=	0.4±0.1	=	0.4±0.3	=
18:0/18:1	2.4±0.7	=	3.0±1.0	=	3.7±0.6	=	4.8±1.8	=	4.3±1.2	=		0.6±0.2	=	1.3±1.4	=	0.7±0.5	=	0.6±0.3	=	2.0±1.7	=

PHEG	*Streblonema corymbiferum*											*Streblonema* sp.									
	5 ˚C		10 ˚C		15 ˚C		20 ˚C		25 ˚C	T	#	5 ˚C		10 ˚C		15 ˚C		20 ˚C		25 ˚C	T
20:5/20:5	0.4±0.3	=	0.3±0.2		0.4±0.1	>	0.2±0.0	=	0.4±0.3	=		2.1±0.9	=	1.1±0.7	=	0.3±0.1	=	0.4±0.3	=	0.2±0.2	>
20:5/20:4	29.0±3.5	=	27.1±1.2	=	28.9±1.8	>	18.5±1.1	=	18.9±3.1	>		34.5±3.0	>	25.6±3.4	>	19.1±1.9	=	18.9±1.3	>	12.8±1.3	>
20:4/20:4	70.5±3.7	=	72.6±1.4	=	70.7±1.7	<	81.3±1.1	=	80.7±2.9	<		63.4±3.3	<	73.3±3.8	<	80.5±1.7	=	80.7±1.0	<	87.0±1.3	<

DGTS	*Streblonema corymbiferum*											*Streblonema* sp.									
	5 ˚C		10 ˚C		15 ˚C		20 ˚C		25 ˚C	T	#	5 ˚C		10 ˚C		15 ˚C		20 ˚C		25 ˚C	T
14:0/14:0	0.1±0.1	=	0.1±0.1		0.1±0.0	<	0.1±0.0	>	0.1±0.0	>		0.1±0.0	=	0.1±0.1	=	0.1±0.1	=	0.1±0.0	=	0.1±0.1	=
14:0/16:1	0.9±0.6	=	0.8±0.3	=	0.6±0.4	=	0.8±0.4	>	0.4±0.0	>		0.1±0.1	=	0.2±0.0	<	0.4±0.2	>	0.2±0.0	=	0.1±0.0	=
15:0/15:0	3.4±0.5	=	3.9±0.2	=	2.8±1.2	=	2.0±2.5	=	0.7±0.3	=		0.1±0.1	=	0.1±0.0	<	0.6±0.2	=	0.7±0.2	=	1.1±1.1	=
14:0/16:0	0.5±0.2	>	+	=	0.1±0.1	=	0.1±0.1	=	0.2±0.2	>		0.2±0.1	=	0.3±0.3	=	0.2±0.1	=	0.4±0.3	=	0.7±0.2	<
15:0/16:0	1.3±0.5	>	0.4±0.2	=	0.4±0.2	=	0.6±0.7	=	0.1±0.0	>		+	=	0.1±0.1	=	0.3±0.2	<	1.2±0.1	=	1.5±1.2	<
14:0/18:3	0.2±0.0	>	+	=	0.1±0.0	=	0.1±0.0	=	0.1±0.1	=		0.1±0.0	=	0.1±0.1	=	+	=	0.1±0.1	=	0.1±0.1	=
16:1/16:1	1.4±0.2	>	1.0±0.1	>	0.5±0.1	=	0.3±0.0	>	0.2±0.0	>		0.4±0.1	=	0.3±0.1	=	0.4±0.0	=	0.2±0.0	=	0.1±0.0	>
14:0/18:1	1.7±0.3	=	1.9±0.3	=	1.4±0.3	<	2.5±0.1	>	1.5±0.2	=		2.2±0.4	=	2.9±0.4	=	2.6±0.5	=	3.1±0.2	>	2.1±0.1	=
16:1/16:0	1.7±0.7	=	1.2±0.3	>	0.7±0.2	=	0.8±0.1	=	0.6±0.2	>		4.0±0.8	>	1.8±0.5	>	0.5±0.3	=	0.6±0.2	=	0.5±0.4	>
15:0/17:0	0.4±0.2	=	0.2±0.1	=	0.2±0.0	=	0.3±0.2	=	0.2±0.1	=		0.1±0.0	=	0.1±0.1	=	0.1±0.0	>	+	=	+	>
16:0/16:0	0.5±0.1	>	0.3±0.1	>	0.1±0.1	<	0.7±0.2	<	1.4±0.2	<		0.7±0.4	=	0.8±0.2	>	0.4±0.0	<	0.7±0.1	<	1.7±0.2	<
15:1/18:1	0.1±0.1	=	+	=	+	=	+	=	+	=		0.1±0.0	=	0.1±0.0	=	0.1±0.0	=	+	=	+	=
17:1/16:1	0.1±0.0	=	+	=	+	=	+	=	+	>		+	=	0.1±0.1	=	+	=	0.1±0.0	=	+	=
15:0/18:1	1.3±0.3	>	0.4±0.2	=	0.4±0.4	=	0.2±0.1	>	0.1±0.0	>		0.1±0.1	=	0.1±0.0	=	0.1±0.1	=	0.1±0.1	=	0.1±0.0	=
17:1/16:0	0.3±0.4	=	0.3±0.1	>	0.1±0.0	<	0.2±0.1	>	0.1±0.0	>		0.2±0.2	=	0.1±0.0	=	0.1±0.1	=	0.1±0.0	=	0.1±0.0	=
16:1/18:4	0.3±0.3	=	+	>	+	<	+	=	+	>		0.1±0.1	=	0.1±0.1	=	+	=	+	=	+	>
18:3/16:1	0.1±0.1	=	0.1±0.0	=	+	<	+	=	+	>		0.1±0.1	=	0.1±0.0	=	+	=	+	=	+	>
16:2/18:2	0.1±0.1	=	+	=	+	=	+	=	+	=		+	=	0.1±0.1	=	+	=	+	=	+	=
18:4/16:0	0.1±0.1	=	+	=	+	=	+	=	0.1±0.0	>		0.1±0.0	=	0.1±0.1	=	+	=	+	=	0.1±0.1	=
16:1/18:2	0.3±0.4	=	0.2±0.1	=	+	=	0.1±0.0	=	0.1±0.0	>		0.2±0.2	=	0.2±0.1	=	0.1±0.0	>	+	=	+	=
18:3/16:0	0.3±0.2	=	+	=	+	=	+	=	+	>		0.1±0.1	=	+	=	+	=	+	=	+	=
18:3/16:0	0.1±0.0	=	0.1±0.1	>	+	=	+	=	+	>		0.1±0.0	=	0.1±0.1	=	+	=	+	=	0.1±0.1	=
16:1/18:1	1.8±0.6	=	1.7±0.3	<	2.2±0.2	=	2.1±0.4	>	1.5±0.2	=		0.9±0.2	=	1.0±0.1	=	0.8±0.2	>	0.5±0.1	<	0.7±0.0	=
18:2/16:0	2.2±0.8	=	1.4±0.2	>	1.0±0.2	<	1.4±0.2	=	1.4±0.4	>		2.3±0.5	<	3.2±0.4	=	2.7±0.3	<	3.2±0.1	=	2.8±0.3	=
16:0/18:1	3.6±1.1	=	3.1±0.4	<	4.0±0.4	<	6.4±0.6	<	8.6±1.5	<		5.2±0.0	=	5.1±0.3	>	4.3±0.4	=	5.0±0.4	<	7.7±1.0	<
18:0/16:0	0.2±0.0	=	0.1±0.0	=	0.1±0.0	<	0.5±0.3	=	0.8±0.2	<		0.3±0.1	=	0.2±0.0	=	0.1±0.0	=	0.1±0.1	=	0.4±0.2	=
17:1/18:1	0.4±0.2	>	0.1±0.1	=	0.1±0.1	=	0.2±0.1	>	0.1±0.0	>		+	=	0.1±0.0	=	+	<	0.1±0.0	=	0.1±0.0	<
17:0/18:1	0.3±0.0	=	0.1±0.1	=	0.2±0.2	=	0.3±0.2	<	0.5±0.0	<		0.3±0.2	=	0.1±0.1	<	0.4±0.0	>	0.1±0.0	<	0.2±0.1	=
18:3/18:3	0.1±0.0	=	0.1±0.1	=	+	<	+	=	+	>		+	=	+	=	+	>	+	=	+	=
18:2/18:4	0.2±0.1	=	0.2±0.1	>	+	=	0.1±0.1	=	0.1±0.0	>		0.1±0.1	=	0.3±0.2	>	0.1±0.1	=	0.1±0.0	=	0.1±0.0	=
16:1/20:5	0.1±0.1	=	+	=	+	=	+	=	+	>		0.1±0.1	=	0.1±0.1	>	+	=	+	<	0.1±0.0	=
18:2/18:3	0.1±0.0	>	+	>	+	=	+	=	+	>		0.1±0.0	>	+	=	+	=	+	>	+	>
18:4/18:1	+	=	+	=	0.1±0.0	>	+	=	0.1±0.0	=		0.1±0.1	=	+	=	+	=	+	=	+	=
18:2/18:2	1.3±0.3	>	0.1±0.0	=	0.1±0.1	=	+	=	0.1±0.0	>		0.2±0.1	=	0.1±0.1	=	0.1±0.0	=	0.1±0.0	=	+	=
18:1/18:2	25.5±3.0	=	21.8±1.0	>	15.7±2.4	>	9.2±0.2	=	8.6±0.8	>		18.5±0.9	<	21.0±0.4	>	12.7±0.5	>	9.4±0.6	>	4.7±1.0	>
18:1/18:1	36.1±0.9	<	54.5±2.7	<	64.2±3.0	=	59.9±2.0	=	55.7±4.1	<		54.0±2.4	=	55.0±1.1	<	68.7±2.0	=	66.4±1.0	=	63.9±3.9	<
16:0/20:1, 18:1/18:0	9.3±1.3	>	3.8±1.1	>	2.0±0.4	<	3.6±0.1	<	11.0±4.0	=		7.1±1.1	>	4.8±1.0	>	2.6±0.4	<	5.5±0.2	<	8.8±0.8	<
18:0/18:0	0.1±0.1	=	0.1±0.1	=	+	=	+	<	0.2±0.1	=		+	<	0.1±0.0	=	0.1±0.1	=	+	=	0.1±0.0	=
18:1/19:1	0.6±0.2	>	0.3±0.1	<	1.1±0.2	<	5.1±0.3	>	3.6±0.7	<		0.2±0.0	=	0.4±0.3	=	0.7±0.1	=	0.8±0.2	=	0.9±0.4	<
19:1/18:1	0.4±0.2	=	0.3±0.1	<	0.9±0.2	=	1.1±0.1	=	0.6±0.4	=		0.2±0.2	=	0.2±0.1	<	0.4±0.1	<	0.5±0.1	=	0.8±0.2	<
18:3/20:3	0.1±0.0	=	+	=	+	=	+	=	+	>		+	=	+	>	+	=	+	=	+	=
18:1/20:5	0.2±0.2	=	0.1±0.0	=	0.1±0.1	=	0.1±0.1	=	0.1±0.1	=		+	<	0.1±0.0	>	+	=	0.1±0.0	<	0.1±0.0	<
16:0/22:6	0.2±0.1	=	0.1±0.0	=	+	=	0.1±0.1	=	0.2±0.3	=		+	=	+	=	+	=	+	<	0.1±0.0	=
18:1/20:1	2.3±0.2	>	0.7±0.2	=	0.4±0.1	=	0.6±0.1	=	0.7±0.2	>		1.4±0.5	>	0.6±0.2	>	0.2±0.1	=	0.3±0.1	<	0.4±0.0	>
18:1/20:0	0.3±0.2	=	0.1±0.1	=	+	=	0.1±0.1	=	0.1±0.1	=		0.1±0.0	=	0.1±0.1	>	+	=	+	=	0.1±0.1	=

Table S2. Molecular species composition of MGDG, DGDG, SQDG, PG, PE, PC, PI, PHEG and DGTS of *Streblonema corymbiferum* and *Streblonema* sp. cultivated at various light intensity (μmol photons m^{-2} s^{-1}). Values given as % of total lipid class, (mean ± s.d. for triplicate). “+” – amount less than 0.1%, “-” – not detected. Between columns result of Tukey test ($p<0.05$, $n=3$) are shown (“>”, “<” – statistically significant difference, “=” – no significant changes), “T” – difference between 0 and 200 μmol photons m^{-2} s^{-1}.

MGDG	*Streblonema corymbiferum*																	*Streblonema* sp.															
	0		5		12		20		50		100		150		200	T	#	0		5		12		20		50		100		150		200	T
16:2/14:0	-	<	+	=	+	=	+	=	+	=	+	>	-	<	+	<		+	>	+	<	+	=	+	=	+	=	+	>	-	<	+	=
14:0/16:1	+	=	+	=	+	=	+	=	+	=	+	>	+	<	+	<		+	=	+	=	+	=	+	=	+	=	+	=	+	=	+	=
14:0/16:0	+	=	+	=	+	=	+	=	+	=	+	>	+	<	+	=		+	=	+	=	+	=	+	=	+	=	+	=	+	=	+	=
16:3/16:4	+	=	+	<	+	=	+	=	+	=	+	=	+	=	+	=		+	=	+	=	+	=	+	=	+	=	+	>	+	<	+	=
16:3/16:3	+	=	0.1±0.0	=	0.1±0.0	>	+	=	+	<	0.1±0.0	=	0.1±0.0	=	0.1±0.0	<		+	=	+	=	+	=	+	=	+	=	+	=	+	=	+	<
16:2/16:3	0.3±0.2	<	0.6±0.1	>	0.4±0.1	>	0.2±0.1	<	0.6±0.1	=	0.5±0.2	=	0.4±0.1	<	0.9±0.2	<		0.1±0.1	=	0.1±0.1	=	0.2±0.2	=	0.3±0.1	=	0.3±0.1	=	0.3±0.1	<	0.5±0.1	=	0.4±0.2	<
14:1/18:4	+	=	+	=	+	=	+	=	+	=	+	=	+	=	0.1±0.1	=		+	>	+	=	+	=	+	=	+	=	+	=	+	=	+	=
18:3/14:1	0.1±0.0	=	+	=	+	=	+	=	+	=	+	=	+	=	+	>		+	=	+	=	+	>	+	=	+	=	+	=	+	=	0.1±0.1	=
16:1/16:3	0.9±0.1	=	1.0±0.2	=	1.0±0.2	=	0.7±0.2	<	1.2±0.1	=	1.3±0.2	>	0.9±0.2	<	1.4±0.2	<		0.3±0.1	=	0.2±0.1	<	0.8±0.2	=	0.8±0.2	>	0.3±0.2	=	0.3±0.1	=	0.5±0.2	=	0.4±0.2	=
14:0/18:4	0.1±0.0	=	0.1±0.0	=	0.1±0.0	=	0.1±0.0	=	0.1±0.0	=	0.1±0.1	=	0.1±0.0	=	0.1±0.1	=		0.1±0.0	=	+	=	0.2±0.2	=	0.3±0.2	=	0.1±0.0	=	0.1±0.1	=	0.1±0.0	=	0.1±0.0	=
16:1/16:2	0.1±0.0	=	0.1±0.1	=	0.2±0.1	=	0.2±0.1	=	0.1±0.1	=	0.3±0.2	=	0.1±0.1	=	0.2±0.0	<		+	=	0.1±0.1	=	0.1±0.1	=	0.3±0.2	=	0.1±0.0	<	0.3±0.1	=	0.2±0.2	=	+	=
14:0/18:3	0.2±0.2	=	0.4±0.1	=	0.3±0.1	=	0.3±0.2	=	0.3±0.2	=	0.6±0.1	=	0.7±0.1	=	0.8±0.4	<		0.1±0.1	=	0.2±0.1	=	0.4±0.1	>	0.2±0.1	=	0.3±0.1	=	0.3±0.1	=	0.2±0.1	<	0.5±0.0	<
16:0/16:3	+	=	+	=	+	=	+	<	+	=	+	=	+	=	+	=		+	>	+	=	+	>	+	=	+	=	+	=	+	=	+	>
16:1/16:1	0.1±0.1	=	0.2±0.1	<	0.5±0.2	>	0.3±0.1	>	0.1±0.0	=	0.1±0.1	=	0.1±0.1	=	0.1±0.0	=		0.1±0.0	<	0.1±0.0	=	0.1±0.1	=	0.2±0.2	=	0.1±0.0	=	+	=	+	=	+	>
14:0/18:2	0.1±0.1	=	0.1±0.1	=	0.1±0.1	=	0.1±0.0	=	0.2±0.1	=	0.1±0.1	=	0.1±0.0	<	0.2±0.1	=		0.1±0.0	=	0.1±0.0	=	0.2±0.1	=	0.2±0.1	=	0.1±0.0	=	0.2±0.1	=	0.1±0.1	=	0.2±0.1	<
14:0/18:1	0.6±0.1	<	1.1±0.1	>	0.8±0.1	=	1.0±0.2	=	1.1±0.2	=	1.4±0.2	=	1.4±0.0	=	1.6±0.3	<		0.6±0.1	=	0.9±0.2	=	1.0±0.2	=	1.0±0.2	>	0.6±0.2	<	0.8±0.1	=	0.8±0.2	<	1.4±0.2	<
16:0/16:1	+	=	+	=	+	=	+	=	+	<	+	>	+	=	+	>		+	=	+	=	+	=	+	=	+	=	+	=	+	=	+	=
16:0/16:0	+	=	+	=	+	<	+	=	+	>	+	=	+	=	+	=		+	=	+	=	+	>	+	<	+	=	+	=	+	=	+	=
16:1/17:3	+	=	+	=	+	=	+	=	+	=	+	=	+	=	+	>		+	=	+	<	+	>	+	=	+	=	+	=	+	=	+	=
15:0/18:4	+	=	+	=	+	=	+	=	+	=	+	=	+	=	+	=		+	=	+	=	+	=	+	<	+	>	+	<	+	>	+	=
15:0/18:4	+	=	+	=	+	=	0.1±0.1	=	+	=	+	=	+	=	+	>		+	=	+	=	0.1±0.1	=	0.1±0.0	>	+	<	+	=	+	=	0.1±0.0	=
15:0/18:3	+	=	+	=	+	=	0.1±0.1	=	+	=	0.1±0.0	=	0.1±0.0	<	0.1±0.0	<		0.1±0.1	=	0.1±0.0	<	0.2±0.0	<	0.3±0.0	>	0.1±0.0	=	0.1±0.0	=	0.1±0.0	=	0.2±0.1	=
15:0/18:2	+	=	+	=	+	=	+	=	+	=	+	=	+	=	+	=		+	>	+	=	+	=	+	=	+	=	+	=	+	=	+	=
18:1/15:0	-	<	+	>	-	<	+	=	+	=	+	=	+	=	+	=		-	<	+	>	-	=	+	=	+	<	+	=	+	=	+	<
15:0/18:1	+	<	+	>	+	=	+	=	+	=	+	=	+	=	0.1±0.0	<		0.1±0.0	=	+	>	+	<	+	=	+	=	+	=	+	=	+	=
15:0/18:1	+	>	+	<	+	=	+	=	+	=	+	=	+	=	+	=		+	=	0.1±0.1	=	0.1±0.0	=	0.1±0.1	=	+	=	+	=	+	=	0.1±0.0	=
17:1/16:0	+	>	+	<	+	>	-	<	+	=	+	=	+	=	+	>		+	=	+	=	+	>	-	<	+	=	+	=	+	=	+	>
16:4/18:4	+	=	+	=	+	=	+	=	+	=	+	=	+	=	0.1±0.0	=		0.1±0.0	>	+	<	0.1±0.0	=	+	>	+	=	+	=	+	=	+	=
16:3/18:4	0.1±0.1	=	0.1±0.1	=	0.1±0.0	=	0.1±0.1	=	0.1±0.1	=	0.1±0.0	=	0.1±0.1	=	0.1±0.0	=		0.2±0.1	=	0.1±0.1	=	0.1±0.1	=	0.1±0.0	=	+	=	+	<	0.1±0.0	=	0.1±0.0	>
18:3/16:4	+	=	+	=	+	>	+	<	+	>	+	=	+	=	+	=		+	=	+	=	+	>	+	<	+	>	+	=	+	=	+	>
18:3/16:3	0.1±0.1	=	0.1±0.1	=	0.2±0.1	=	0.1±0.1	=	0.1±0.1	=	0.1±0.0	>	0.1±0.0	=	0.2±0.1	=		0.1±0.0	<	0.2±0.1	=	0.2±0.1	=	0.1±0.1	=	0.1±0.0	=	0.1±0.1	=	0.1±0.0	=	0.1±0.1	=
16:2/18:4	0.1±0.0	=	0.1±0.0	=	+	=	0.1±0.1	=	0.1±0.0	=	+	<	0.1±0.0	>	+	>		0.1±0.0	=	0.1±0.0	>	+	=	+	=	0.1±0.0	>	+	=	+	=	+	>
16:2/18:3	+	=	+	=	+	=	+	=	+	=	+	=	+	>	+	=		+	=	+	=	+	=	+	=	+	=	+	<	+	=	+	=
18:4/16:1	0.2±0.1	=	0.2±0.1	=	0.1±0.1	=	0.1±0.0	=	0.1±0.1	=	0.1±0.0	>	0.1±0.0	<	0.1±0.1	=		0.1±0.0	=	0.1±0.1	=	0.1±0.0	=	0.2±0.1	=	0.1±0.1	=	0.1±0.1	=	0.1±0.0	=	0.1±0.0	=
16:1/18:4	0.3±0.1	=	0.2±0.1	=	0.2±0.0	=	0.2±0.1	<	0.3±0.1	>	0.2±0.1	=	0.2±0.1	=	0.2±0.0	=		0.1±0.0	=	0.1±0.1	=	0.1±0.0	=	0.1±0.0	>	0.1±0.0	=	0.1±0.1	=	0.1±0.0	=	0.1±0.1	=
18:1/16:4	0.1±0.0	=	0.1±0.1	=	0.1±0.1	=	0.1±0.0	=	0.1±0.0	=	0.1±0.0	=	0.1±0.0	<	0.2±0.1	<		+	=	0.1±0.1	=	0.1±0.1	=	+	=	+	<	0.1±0.0	>	0.1±0.0	=	+	=
20:5/14:0	0.1±0.0	=	0.1±0.0	>	+	=	+	=	+	=	0.1±0.1	=	0.1±0.1	=	0.1±0.1	=		+	=	+	=	+	=	+	=	+	=	+	=	+	=	+	=
14:0/20:5	0.1±0.1	=	0.1±0.0	=	0.1±0.1	=	0.1±0.1	=	0.1±0.0	=	0.1±0.1	=	0.1±0.1	=	0.1±0.0	=		+	=	+	=	+	<	0.1±0.0	>	+	=	+	=	+	=	+	=
18:5/16:0	+	=	+	=	+	=	+	=	+	=	+	<	0.1±0.0	=	0.1±0.0	<		0.1±0.0	>	0.1±0.0	=	0.1±0.0	=	0.1±0.0	>	+	=	+	=	+	<	0.1±0.0	>
18:3/16:1	0.2±0.1	=	0.3±0.0	=	0.2±0.0	=	0.2±0.0	>	0.1±0.0	=	0.2±0.1	=	0.1±0.1	=	0.2±0.1	=		0.1±0.0	=	0.1±0.0	=	0.1±0.0	=	0.1±0.1	=	0.1±0.1	=	0.1±0.1	=	0.1±0.1	=	0.1±0.0	=

16:1/18:3	0.1±0.1	=	0.1±0.0	<	0.2±0.1	>	0.1±0.0	=	0.1±0.0	=	0.2±0.1	>	0.1±0.0	<	0.1±0.0	=	0.1±0.1	=	0.1±0.0	=	0.1±0.1	=	0.2±0.1	=	0.1±0.1	=	0.1±0.0	=	0.1±0.0	=	0.1±0.1	=
18:1/16:3	0.1±0.0	=	+	=	0.1±0.0	=	0.1±0.1	=	+	=	+	=	+	=	0.1±0.1	=	0.1±0.0	=	0.1±0.0	=	0.1±0.1	=	0.1±0.0	=	0.1±0.0	=	0.1±0.0	=	0.1±0.1	=	+	=
14:0/20:4	0.1±0.1	=	0.1±0.1	>	+	=	0.1±0.0	<	0.2±0.1	=	0.1±0.1	=	0.1±0.0	=	0.1±0.0	=	0.1±0.0	=	0.1±0.1	=	0.2±0.1	=	0.3±0.0	>	0.1±0.0	<	0.2±0.1	=	0.3±0.2	=	0.1±0.1	=
16:0/18:4	0.1±0.0	>	0.1±0.0	=	0.1±0.1	=	0.2±0.0	=	0.1±0.0	=	0.1±0.0	>	0.1±0.0	=	0.2±0.1	=	0.1±0.0	=	0.1±0.1	=	0.2±0.2	=	0.4±0.2	>	0.2±0.0	=	0.2±0.1	=	0.1±0.0	=	0.1±0.1	=
18:2/16:1	+	=	+	=	0.1±0.0	=	+	=	+	=	+	=	+	<	+	=	+	=	+	=	+	=	+	=	+	=	+	=	+	=	+	=
16:1/18:2	+	=	+	=	+	>	+	<	+	=	+	=	+	=	+	=	+	=	+	=	+	=	+	=	+	=	+	=	+	>	+	=
18:1/16:2	+	=	+	=	+	=	+	=	+	=	+	=	+	<	+	=	+	=	+	=	+	=	+	=	+	=	+	=	+	=	+	>
16:0/18:3	0.4±0.2	=	0.3±0.2	=	0.3±0.1	=	0.5±0.2	=	0.5±0.2	=	0.6±0.1	=	0.4±0.3	<	1.2±0.2	<	0.4±0.1	=	0.6±0.3	<	1.0±0.2	=	1.1±0.3	=	1.4±0.4	=	1.4±0.3	=	1.8±0.5	=	2.3±0.5	<
18:0/16:3	+	=	0.1±0.2	=	+	=	+	=	+	=	+	>	+	<	+	=	+	=	0.1±0.2	=	+	=	+	=	+	=	+	=	+	=	+	>
18:1/16:1	+	=	+	=	+	=	+	=	+	=	+	=	+	=	+	=	+	=	+	=	+	=	+	=	+	=	+	=	+	=	+	=
18:1/16:1	+	=	+	=	+	=	+	=	+	=	0.1±0.0	=	0.1±0.1	=	0.1±0.1	=	+	<	0.2±0.1	=	0.1±0.1	=	0.2±0.2	=	0.4±0.1	=	0.3±0.2	=	0.3±0.0	=	0.1±0.0	<
16:0/18:2	+	=	0.1±0.0	=	+	=	+	=	0.1±0.0	=	0.1±0.0	<	0.1±0.0	=	0.1±0.1	<	0.2±0.1	=	0.2±0.0	=	0.3±0.2	=	0.4±0.1	=	0.4±0.1	=	0.4±0.1	=	0.7±0.4	=	1.2±0.4	<
16:0/18:1	0.3±0.1	=	0.3±0.1	=	0.4±0.0	<	0.4±0.1	<	0.6±0.0	<	0.8±0.2	=	0.8±0.2	=	1.1±0.2	<	1.3±0.1	<	1.6±0.2	=	1.3±0.1	=	1.4±0.2	=	1.8±0.4	=	2.0±0.3	=	2.3±0.2	<	3.9±0.3	<
16:0/18:0	+	=	+	=	+	=	+	=	+	=	+	=	+	=	+	>	+	=	+	=	+	=	+	=	+	=	+	=	0.1±0.2	=	0.2±0.2	=
18:3/17:3	+	=	+	=	+	=	+	=	+	=	+	=	+	=	+	=	+	<	+	=	+	=	+	=	+	=	+	=	+	=	+	=
18:4/17:2	+	=	+	=	+	=	+	<	+	=	+	=	+	>	+	=	+	<	+	>	+	=	+	>	+	<	+	=	+	=	+	=
17:2/18:4	+	=	+	=	+	=	+	=	+	=	+	=	+	<	+	=	+	<	+	=	+	<	+	>	+	=	+	<	+	>	+	=
17:2/18:3	+	<	+	>	+	=	+	=	+	>	+	=	+	=	+	=	+	=	+	=	+	=	+	=	+	=	+	=	+	=	+	=
17:1/18:3	+	=	+	=	+	=	+	=	+	=	+	=	+	=	+	=	+	=	+	>	+	=	+	=	+	=	+	=	+	=	+	=
15:0/20:4	+	>	+	=	+	=	+	=	+	=	+	=	+	=	+	=	+	=	+	=	+	=	+	=	+	=	+	=	+	=	+	<
17:0/18:3	+	>	+	=	+	>	+	<	+	=	+	=	+	>	+	>	+	<	+	>	+	=	+	=	+	=	+	=	+	=	+	<
18:1/17:1	+	=	+	=	+	=	+	=	+	=	+	=	+	<	+	=	+	>	+	=	+	=	+	=	+	=	+	=	+	=	+	>
17:0/18:2	+	=	+	=	+	=	+	=	+	=	+	=	+	<	+	=	+	=	+	<	+	=	+	=	+	=	+	=	+	=	+	=
17:0/18:1	+	=	+	=	+	=	+	<	+	=	+	>	+	<	+	<	+	=	+	=	+	=	+	=	+	<	0.1±0.0	=	+	=	+	=
20:5/16:4	0.6±0.1	=	0.6±0.0	>	0.4±0.1	>	0.3±0.1	=	0.3±0.0	=	0.4±0.1	=	0.3±0.1	<	0.7±0.2	=	0.1±0.1	=	0.1±0.0	=	+	=	0.1±0.0	=	0.1±0.0	=	0.1±0.0	=	0.1±0.1	=	0.2±0.1	=
18:4/18:4	13.1±0.3	<	13.4±0.1	=	13.6±0.1	>	12.6±0.5	=	12.8±0.2	<	13.6±0.7	=	13.3±0.1	<	15.2±1.1	<	20.4±1.0	=	19.8±1.1	<	23.6±2.4	=	20.7±2.8	>	17.6±1.1	=	18.2±1.5	=	20.1±2.1	=	20.8±1.4	=
18:3/18:4	17.0±0.5	<	17.5±0.2	=	18.0±1.6	=	18.6±0.5	>	17.8±0.6	=	17.3±0.1	=	17.6±0.8	>	16.7±0.5	=	26.8±1.0	=	25.7±2.4	=	23.3±1.4	=	22.2±1.8	=	22.7±2.2	=	22.2±2.0	=	23.8±1.2	=	23.5±1.1	>
18:3/18:3	6.3±0.0	=	6.2±0.3	=	6.7±0.4	<	7.4±0.1	<	8.8±0.5	=	8.5±0.3	=	8.8±0.2	=	8.6±0.2	<	5.9±0.3	=	5.8±0.4	>	4.9±0.5	=	5.5±0.5	<	10.3±0.2	=	11.0±0.8	=	10.6±0.8	<	12.7±1.0	<
18:2/18:4	2.2±0.4	=	2.4±0.3	<	3.5±0.5	=	3.5±0.1	=	3.1±0.3	>	2.6±0.3	=	2.6±0.6	>	1.8±0.2	=	3.0±0.6	=	2.6±0.5	=	3.2±0.6	=	3.5±0.7	<	4.7±0.9	=	3.9±1.0	=	3.4±0.5	>	2.5±0.5	=
20:5/16:1	0.8±0.1	<	1.0±0.1	<	1.1±0.1	>	0.6±0.2	>	0.3±0.1	=	0.3±0.1	=	0.3±0.1	=	0.2±0.1	>	0.3±0.1	=	0.3±0.4	=	0.5±0.1	=	0.3±0.1	=	0.2±0.1	=	0.1±0.1	=	0.1±0.1	=	+	>
18:3/18:2	3.1±0.3	=	2.9±0.4	<	4.1±0.2	<	5.0±0.4	=	4.9±0.4	>	4.3±0.2	=	4.5±0.8	>	3.1±0.2	=	1.7±0.4	<	2.5±0.1	=	2.7±0.6	=	3.3±1.3	<	8.0±0.8	=	7.5±2.0	=	6.1±0.5	>	5.1±0.2	<
18:2/18:3	0.1±0.1	=	0.1±0.1	=	0.3±0.3	=	0.3±0.2	=	0.3±0.1	=	0.1±0.1	<	0.3±0.0	>	0.2±0.1	=	0.1±0.0	=	0.1±0.1	=	0.1±0.0	<	0.4±0.1	>	0.1±0.0	=	+	=	0.2±0.2	=	+	>
18:1/18:4	0.2±0.1	<	0.4±0.1	=	0.3±0.2	=	0.3±0.1	=	0.2±0.2	=	0.3±0.1	=	0.3±0.2	=	0.2±0.1	=	0.1±0.1	=	0.1±0.1	=	0.1±0.1	=	0.1±0.1	=	+	=	+	=	+	=	0.1±0.0	=
20:5/16:0	0.3±0.1	=	0.2±0.1	=	0.3±0.2	=	0.4±0.1	=	0.4±0.2	=	0.2±0.1	<	0.3±0.0	>	0.2±0.1	=	0.1±0.0	=	0.1±0.0	=	0.1±0.1	=	0.1±0.1	=	+	=	+	=	+	=	+	>
16:0/20:5	0.5±0.1	<	0.7±0.1	=	0.6±0.2	=	0.6±0.2	>	0.2±0.1	=	0.2±0.1	=	0.2±0.0	=	0.2±0.0	>	0.1±0.0	=	0.2±0.2	=	0.1±0.1	<	0.3±0.1	>	0.1±0.1	=	0.1±0.1	=	0.2±0.1	<	0.3±0.0	<
18:2/18:2	0.4±0.3	=	0.3±0.2	=	0.5±0.2	<	0.7±0.1	=	0.4±0.4	=	0.4±0.1	=	0.8±0.5	>	0.1±0.1	>	0.3±0.3	=	0.3±0.0	<	0.8±0.2	=	0.7±0.4	<	2.4±0.3	=	2.0±0.5	>	1.3±0.0	>	0.7±0.3	=
18:1/18:3	2.6±0.1	=	2.7±0.2	<	3.2±0.5	=	3.2±0.4	=	3.5±0.5	>	2.8±0.1	=	2.8±0.5	=	2.4±0.5	=	1.0±0.4	=	0.6±0.3	=	0.6±0.3	=	0.7±0.2	<	1.0±0.2	=	1.1±0.1	=	1.1±0.2	=	1.1±0.0	=
16:0/20:4	+	=	+	=	+	=	+	=	+	=	+	<	0.1±0.1	>	+	=	0.1±0.1	=	+	=	+	=	+	=	+	=	+	=	0.1±0.0	=	+	=
16:0/20:4	+	=	0.1±0.1	=	0.1±0.0	=	0.1±0.0	>	+	=	+	=	+	=	+	=	0.2±0.1	>	0.1±0.0	=	0.1±0.0	=	0.1±0.1	=	0.1±0.0	=	0.1±0.1	=	0.1±0.0	=	0.1±0.0	>
18:1/18:2	0.4±0.2	=	0.5±0.1	<	0.7±0.1	=	0.7±0.1	=	0.7±0.1	>	0.5±0.1	=	0.6±0.2	=	0.4±0.1	=	0.1±0.1	=	0.1±0.0	=	0.2±0.0	=	0.2±0.0	<	0.4±0.1	=	0.4±0.1	=	0.4±0.1	=	0.5±0.1	<
20:3/16:0	+	=	+	=	+	=	+	=	+	=	0.1±0.0	>	+	<	+	<	+	=	+	=	+	=	+	=	0.1±0.0	=	+	=	+	=	+	=
18:0/18:3	+	=	+	=	+	=	0.1±0.0	=	0.1±0.0	>	0.1±0.0	>	+	<	0.1±0.0	<	+	<	+	<	0.1±0.0	=	0.1±0.0	=	0.1±0.1	=	0.1±0.0	=	0.1±0.0	=	0.1±0.0	<
18:1/18:1	+	>	+	=	+	=	+	<	0.1±0.0	=	0.1±0.0	=	0.1±0.0	=	0.1±0.0	=	+	>	+	=	+	=	+	=	+	=	+	=	+	=	+	=
18:1/18:1	+	=	0.1±0.0	<	0.2±0.1	<	0.3±0.1	>	0.1±0.0	=	0.1±0.1	=	0.1±0.0	=	0.1±0.1	=	+	=	+	=	+	=	+	=	+	>	+	<	0.1±0.0	=	0.1±0.0	=
18:0/18:2	+	=	+	=	+	=	+	=	+	=	0.1±0.1	=	+	=	+	=	+	=	+	>	+	<	+	=	+	=	+	>	+	=	+	=
18:0/18:1	+	<	+	<	0.1±0.0	=	0.1±0.0	=	0.1±0.0	=	0.1±0.0	=	0.1±0.0	=	0.1±0.0	<	+	=	0.1±0.1	=	0.1±0.0	>	+	<	0.1±0.0	=	0.1±0.1	=	0.1±0.0	=	0.1±0.0	=

18:0/18:0	+	=	+	=	+	=	+	=	+	>	-	<	+	>	-	>		+	>	+	=	+	=	+	<	+	=	-	=	+	>	-	>
18:4/19:2	+	=	+	=	+	=	+	=	+	=	+	=	+	=	+	=		+	<	+	=	+	=	+	=	+	=	+	=	+	=	+	=
19:2/18:4	+	=	+	=	+	=	+	=	-	<	+	>	-	<	+	=		+	=	+	=	+	=	+	>	-	<	+	>	-	<	+	=
18:3/19:2	+	=	+	=	+	=	0.1±0.0	=	0.1±0.0	=	+	=	+	=	+	=		+	>	+	=	+	<	+	=	+	=	+	=	+	=	+	=
19:2/18:3	+	>	+	=	+	=	+	=	+	=	+	=	+	=	-	>		+	=	+	=	+	=	+	=	+	=	+	=	+	>	-	>
18:4/19:1	+	=	+	<	+	>	+	=	+	>	+	=	+	>	-	=		+	=	+	=	+	=	+	=	+	=	+	=	+	>	-	>
18:3/19:1	0.1±0.0	=	0.1±0.0	=	+	=	0.1±0.1	=	0.1±0.0	=	0.1±0.0	=	0.1±0.1	=	+	=		+	>	+	=	+	=	+	=	+	=	+	=	+	=	+	=
20:5/18:4	18.0±1.4	>	16.4±0.1	=	15.3±1.7	=	14.8±0.6	>	13.3±0.3	<	14.8±0.7	=	15.0±0.7	<	16.6±0.7	=		16.2±0.8	=	16.0±1.2	=	16.1±1.9	=	15.0±1.3	>	8.6±1.0	=	8.6±1.2	=	8.6±0.9	>	7.3±0.6	>
20:5/18:3	10.8±0.9	=	10.9±0.7	>	8.5±0.5	=	8.5±0.8	=	8.5±0.7	<	9.4±0.3	=	9.2±1.5	=	10.4±0.4	=		6.3±0.3	>	5.6±0.5	>	3.9±0.9	=	4.4±0.7	<	5.4±0.4	=	5.7±0.5	=	5.6±0.2	=	5.5±0.1	>
20:4/18:4	3.3±0.2	<	3.6±0.1	<	4.2±0.5	=	4.1±0.1	>	2.8±0.2	>	2.3±0.1	<	2.5±0.2	>	1.6±0.2	>		1.3±0.3	<	2.1±0.4	=	2.5±0.4	=	2.5±0.2	>	1.2±0.4	=	1.2±0.3	=	1.0±0.4	=	0.8±0.3	>
20:4/18:3	0.7±0.1	>	0.4±0.1	=	0.3±0.1	=	0.3±0.2	<	0.5±0.1	=	0.6±0.1	=	0.4±0.2	=	0.4±0.3	>		0.3±0.2	=	0.3±0.1	=	0.3±0.0	=	0.3±0.1	=	0.3±0.1	=	0.4±0.2	=	0.5±0.2	=	0.5±0.1	=
20:5/18:2	4.1±0.1	>	3.7±0.2	=	3.7±0.3	=	3.8±0.1	<	4.5±0.2	>	3.8±0.4	=	3.7±0.3	>	2.7±0.4	>		2.1±0.2	=	2.1±0.2	>	1.5±0.4	<	2.4±0.6	=	3.0±0.1	=	3.0±0.4	>	2.3±0.2	>	1.8±0.2	=
20:3/18:3	0.4±0.1	>	0.2±0.1	<	0.4±0.0	=	0.3±0.1	=	0.4±0.1	=	0.3±0.1	=	0.4±0.2	=	0.3±0.2	=		0.1±0.0	=	0.1±0.1	<	0.2±0.1	=	0.2±0.1	<	0.5±0.2	=	0.5±0.1	=	0.5±0.1	>	0.2±0.1	<
20:4/18:2	0.4±0.2	>	0.1±0.1	=	0.4±0.2	=	0.3±0.1	=	0.4±0.2	=	0.3±0.2	=	0.4±0.2	=	0.2±0.2	=		0.3±0.2	=	0.4±0.2	=	0.2±0.0	=	0.3±0.1	=	0.4±0.2	=	0.4±0.3	=	0.1±0.0	>	0.1±0.0	=
20:5/18:1	2.5±0.4	=	2.7±0.5	>	1.7±0.3	=	1.5±0.1	<	2.0±0.2	<	2.5±0.4	=	2.3±0.2	=	2.1±0.2	=		0.6±0.3	=	0.3±0.2	=	0.3±0.1	=	0.2±0.1	=	0.2±0.1	=	0.3±0.1	=	0.3±0.0	=	0.3±0.1	=
20:3/18:2	0.1±0.0	>	0.1±0.0	=	0.1±0.1	=	0.1±0.0	=	0.1±0.1	=	0.1±0.1	=	0.1±0.1	>	+	>		+	=	+	<	0.1±0.0	=	+	<	0.2±0.1	=	0.2±0.1	=	0.1±0.0	>	0.1±0.0	=
20:4/18:1	0.3±0.1	=	0.2±0.1	=	0.1±0.1	=	0.2±0.0	<	0.3±0.0	=	0.3±0.0	>	0.2±0.1	=	0.2±0.1	=		0.1±0.1	=	+	=	+	=	+	=	0.1±0.0	=	0.1±0.0	=	+	=	0.1±0.0	=
20:4/18:1	0.7±0.1	=	0.4±0.2	=	0.4±0.2	=	0.6±0.1	<	0.7±0.1	>	0.5±0.1	=	0.6±0.2	>	0.2±0.1	>		0.1±0.0	=	+	=	+	=	+	<	+	<	0.1±0.0	=	0.1±0.0	>	+	>
20:5/18:0	+	=	0.1±0.1	=	+	>	+	=	+	=	+	=	+	=	0.1±0.1	=		0.1±0.1	=	+	=	+	=	+	=	+	=	+	<	+	=	+	=
20:3/18:1	+	=	+	<	0.1±0.0	=	0.1±0.0	=	0.1±0.1	=	0.1±0.0	=	0.1±0.0	=	0.1±0.1	<		+	=	+	<	+	=	+	<	0.1±0.0	=	+	=	+	=	0.1±0.0	=
20:5/20:5	0.4±0.2	<	0.6±0.1	>	0.4±0.1	=	0.4±0.1	=	0.5±0.1	=	0.5±0.3	=	0.5±0.2	=	0.7±0.3	=		1.3±0.2	<	1.7±0.2	>	1.4±0.1	=	1.6±0.2	>	0.7±0.0	=	0.8±0.2	=	0.8±0.2	=	0.6±0.2	>
20:5/20:4	0.2±0.1	=	0.1±0.0	=	0.2±0.0	>	0.1±0.1	=	0.1±0.1	<	0.2±0.1	>	+	<	0.1±0.0	>		0.1±0.1	=	0.2±0.1	=	0.3±0.1	=	0.3±0.1	>	0.1±0.0	=	0.1±0.0	=	0.1±0.0	=	0.1±0.0	=
20:4/20:5	1.9±0.2	=	1.9±0.1	=	1.7±0.4	=	1.5±0.1	=	1.5±0.1	<	1.9±0.0	=	1.7±0.3	=	1.7±0.3	=		3.2±0.2	=	3.3±0.5	=	2.8±0.3	=	3.3±0.4	>	1.9±0.2	=	1.7±0.2	=	1.7±0.1	>	1.3±0.3	>
20:3/20:5	0.4±0.2	=	0.2±0.1	<	0.3±0.1	=	0.4±0.1	>	0.3±0.0	=	0.1±0.1	=	0.2±0.1	=	0.2±0.1	=		0.4±0.2	=	0.3±0.1	>	0.1±0.0	=	0.2±0.1	>	0.1±0.1	=	0.1±0.1	=	0.1±0.1	=	0.1±0.1	>
20:4/20:4	2.3±0.1	=	2.5±0.2	>	2.1±0.3	=	2.0±0.2	<	2.3±0.1	>	2.1±0.2	=	2.1±0.2	>	1.5±0.2	>		1.8±0.4	=	2.3±0.3	>	1.6±0.4	=	1.8±0.3	>	1.4±0.1	=	1.3±0.4	=	1.0±0.1	>	0.6±0.2	>
20:4/20:3	0.1±0.0	=	0.1±0.0	=	+	=	+	=	+	=	+	=	+	=	+	>		+	=	0.1±0.0	>	+	<	0.1±0.0	>	+	=	+	>	+	=	+	=
20:3/20:4	+	=	+	=	+	=	+	=	+	=	+	=	+	=	+	=		+	=	+	>	+	=	+	=	+	=	+	=	+	=	+	=

DGDG	*Streblonema corymbiferum*																	*Streblonema* sp.															
	0		5		12		20		50		100		150		200	T	#	0		5		12		20		50		100		150		200	T
14:0/16:1	0.1±0.0	=	0.1±0.1	=	0.1±0.0	=	0.1±0.1	=	0.1±0.0	=	0.1±0.0	=	0.1±0.1	=	0.1±0.0	=		0.1±0.0	=	0.1±0.1	=	+	=	0.2±0.1	=	0.1±0.0	=	+	=	0.1±0.0	=	0.1±0.1	=
14:0/16:0	0.1±0.1	=	+	=	+	=	0.1±0.0	>	+	=	+	=	+	=	+	=		0.1±0.0	=	0.1±0.0	=	0.1±0.0	=	0.1±0.1	=	0.1±0.1	=	+	=	+	=	+	>
16:1/16:4	0.1±0.1	=	0.1±0.0	=	+	=	+	=	+	<	0.1±0.0	=	+	=	+	=		0.2±0.0	>	+	=	+	=	+	=	+	=	+	=	+	=	+	>
16:3/16:1	+	=	0.1±0.0	=	0.1±0.0	=	+	=	+	=	+	=	+	=	+	=		0.2±0.1	>	+	=	0.1±0.0	=	+	=	+	=	0.1±0.0	>	+	=	+	>
14:0/18:4	0.3±0.2	=	0.1±0.0	<	0.3±0.1	=	0.2±0.1	>	0.1±0.0	=	0.1±0.1	=	0.1±0.1	=	0.1±0.0	=		0.1±0.0	=	0.1±0.1	=	0.4±0.6	=	0.1±0.1	=	0.1±0.0	=	0.1±0.1	=	0.2±0.2	=	0.1±0.1	=
16:1/16:2	0.3±0.2	=	0.3±0.1	=	0.3±0.1	>	0.1±0.0	=	0.1±0.1	=	0.1±0.0	=	0.1±0.1	=	0.2±0.1	=		0.1±0.1	=	0.1±0.1	=	0.2±0.1	=	0.1±0.1	=	0.1±0.0	>	+	=	0.1±0.1	=	0.1±0.0	=
14:0/18:3	1.7±0.4	<	2.4±0.4	=	2.6±0.4	=	2.3±0.3	>	1.9±0.2	=	1.9±0.1	=	2.0±0.6	<	2.6±0.1	<		1.8±0.4	=	2.4±0.8	<	4.7±0.8	=	4.0±0.9	>	2.3±0.6	=	2.5±0.5	=	2.5±0.4	=	2.1±0.2	=
16:1/16:1	0.1±0.1	<	0.7±0.3	=	0.9±0.4	=	0.6±0.3	>	0.2±0.2	=	0.3±0.2	=	0.2±0.0	=	0.1±0.0	=		0.5±0.1	=	0.6±0.4	=	0.8±0.5	=	0.4±0.1	>	0.1±0.0	=	0.1±0.1	=	0.1±0.1	=	0.2±0.1	>
14:0/18:2	0.7±0.2	=	0.4±0.2	=	0.3±0.2	<	0.8±0.2	>	0.2±0.1	=	0.4±0.2	=	0.5±0.2	=	0.3±0.2	>		0.4±0.2	=	0.3±0.1	<	1.5±0.2	=	1.4±0.1	>	0.5±0.2	=	0.4±0.2	=	0.5±0.1	>	0.2±0.2	=
14:0/18:1	1.2±0.5	<	3.3±0.6	=	3.3±0.2	=	3.2±0.3	>	2.3±0.6	=	2.5±0.1	=	2.5±0.2	>	2.1±0.2	<		2.3±0.4	=	2.1±0.5	=	2.0±0.1	=	1.9±0.3	>	0.7±0.4	=	0.5±0.3	=	0.7±0.3	=	0.4±0.2	>
16:0/16:1	1.1±0.0	>	-	#	-	<	+	>	-	#	-	#	-	<	+	>		0.1±0.0	>	-	<	+	=	+	=	+	=	0.2±0.2	=	+	=	0.2±0.3	=
16:0/16:0	-	<	+	>	+	=	+	=	+	=	+	=	+	=	+	<		0.1±0.0	=	0.1±0.0	>	+	>	+	<	+	=	+	>	-	<	+	>
15:0/18:4	-	<	+	=	+	=	+	=	+	>	+	=	+	=	+	<		-	<	0.1±0.0	=	0.1±0.0	=	0.1±0.0	=	+	=	+	=	+	=	+	<
15:0/18:3	0.1±0.1	=	+	=	0.1±0.0	=	0.1±0.1	=	+	=	+	=	+	=	0.1±0.1	=		0.2±0.1	=	0.3±0.2	=	0.1±0.0	<	0.2±0.0	>	0.1±0.0	=	0.1±0.1	=	0.1±0.1	=	0.1±0.1	=
15:0/18:2	+	=	+	=	+	=	+	=	+	=	+	>	+	=	+	>		0.1±0.0	>	+	<	0.1±0.1	>	+	>	+	=	+	=	0.1±0.1	=	+	>

15:0/18:1	0.1±0.0 =	0.1±0.1 =	0.1±0.1 =	0.1±0.0 =	+ =	+ <	0.1±0.0 =	0.1±0.0 =	0.1±0.0 <	0.1±0.0 >	0.1±0.0 =	0.1±0.1 =	+ >	+ <	+ =	+ >
16:3/18:3	0.1±0.0 =	0.1±0.1 =	0.1±0.0 >	+ =	+ =	+ =	+ <	+ >	0.1±0.0 =	0.1±0.0 =	0.1±0.1 =	+ =	+ =	+ =	+ =	0.1±0.0 =
18:4/16:2	- =	0.2±0.2 =	+ =	+ =	0.1±0.1 =	+ =	+ =	0.1±0.0 <	- <	0.1±0.0 =	0.1±0.0 =	0.1±0.0 =	+ =	+ =	+ =	+ =
16:2/18:3	0.1±0.1 =	0.1±0.1 =	0.1±0.0 =	0.1±0.1 =	+ =	+ =	+ =	+ >	0.1±0.0 =	0.1±0.1 =	0.1±0.0 =	0.1±0.1 =	0.1±0.0 =	0.1±0.0 =	0.1±0.0 =	+ =
18:4/16:1	0.1±0.0 <	0.2±0.1 >	0.1±0.0 >	+ =	+ =	+ =	+ =	+ =	0.1±0.1 >	+ <	0.1±0.0 =	0.1±0.0 =	+ =	0.1±0.1 =	+ =	+ >
20:5/14:0	0.2±0.1 >	0.1±0.0 =	0.1±0.1 =	0.1±0.0 =	0.1±0.1 =	0.2±0.1 =	0.1±0.1 <	0.3±0.1 =	+ =	0.1±0.0 =	0.1±0.0 =	0.1±0.0 =	0.1±0.1 =	+ =	+ =	0.1±0.0 =
16:1/18:3	0.5±0.1 =	0.5±0.1 >	0.3±0.0 =	0.3±0.1 =	0.2±0.0 =	0.2±0.1 =	0.2±0.1 =	0.3±0.1 >	0.5±0.4 =	0.2±0.2 =	0.2±0.1 =	0.4±0.1 >	0.1±0.0 =	0.2±0.0 <	0.3±0.1 >	0.1±0.1 =
16:0/18:4	0.4±0.2 =	0.2±0.1 =	0.3±0.1 =	0.3±0.1 =	0.2±0.1 =	0.1±0.0 =	0.1±0.0 =	0.1±0.0 =	0.2±0.0 =	0.3±0.1 <	1.4±0.4 =	1.5±0.5 >	0.4±0.3 =	0.5±0.2 =	0.4±0.3 =	0.2±0.1 =
16:1/18:2	0.1±0.1 =	0.1±0.1 =	0.1±0.0 =	+ =	+ =	+ =	+ =	+ =	0.4±0.0 >	0.1±0.0 =	0.1±0.0 >	+ =	+ =	0.1±0.0 =	0.1±0.1 =	0.1±0.1 >
14:0/20:3	0.2±0.1 =	0.1±0.1 =	0.1±0.0 =	+ =	+ =	0.1±0.1 =	+ =	+ >	0.2±0.1 =	0.2±0.2 =	0.3±0.4 =	0.1±0.0 =	+ =	0.2±0.2 =	0.4±0.5 =	0.1±0.0 =
16:0/18:3	1.4±0.2 >	1.0±0.2 <	1.8±0.3 =	1.8±0.5 >	0.8±0.3 <	1.4±0.2 =	1.3±0.2 <	2.0±0.2 <	1.2±0.3 <	4.6±0.4 <	5.6±0.1 =	5.5±0.8 =	4.8±1.0 =	5.4±0.8 =	5.9±0.5 =	5.7±0.2 <
16:0/18:2	0.5±0.5 =	0.1±0.1 =	0.1±0.1 =	0.2±0.1 =	0.2±0.0 =	0.2±0.0 >	0.1±0.0 <	0.2±0.0 =	0.4±0.2 <	1.1±0.2 >	0.6±0.4 =	1.0±0.2 =	1.0±0.6 =	1.3±0.2 =	1.2±0.1 =	1.2±0.2 <
16:0/18:1	0.5±0.1 =	0.6±0.2 =	0.7±0.4 =	0.9±0.3 >	0.5±0.1 =	0.7±0.2 =	0.7±0.1 <	0.9±0.0 <	3.3±0.6 >	2.2±0.6 >	1.4±0.3 =	1.7±0.5 =	1.4±0.4 =	1.5±0.4 <	1.9±0.1 =	2.2±0.5 >
20:5/16:4	- <	+ >	- <	+ >	+ =	+ =	+ =	+ <	- #	- #	- <	+ =	0.1±0.0 >	+ =	+ =	+ <
18:4/18:4	1.1±0.2 =	0.9±0.2 <	1.3±0.3 =	1.1±0.2 <	1.7±0.2 =	1.9±0.1 =	1.6±0.3 =	1.6±0.2 <	1.7±0.2 >	0.6±0.3 =	0.9±0.3 =	1.3±0.2 =	1.0±0.4 =	1.0±0.1 =	1.1±0.4 =	1.4±0.6 =
18:3/18:4	5.8±0.7 =	4.0±1.7 =	3.2±0.8 =	2.4±1.2 =	2.7±0.4 >	1.4±0.3 <	3.1±0.6 =	3.0±0.5 >	8.0±1.8 >	5.8±0.6 >	3.2±0.2 <	4.2±0.9 >	2.5±0.8 <	3.7±0.8 >	1.7±0.2 <	4.0±0.4 >
20:5/16:2	3.4±0.5 >	2.2±0.7 =	1.9±0.3 =	1.8±1.5 =	2.3±0.2 <	2.9±0.4 >	1.5±0.1 =	1.4±0.6 >	1.5±0.9 >	0.5±0.2 <	2.1±0.6 >	1.0±0.5 <	1.9±0.1 >	1.0±0.5 <	2.8±0.4 >	0.5±0.3 >
16:3/20:4	0.2±0.2 =	+ >	+ =	+ =	+ =	+ =	0.1±0.1 =	0.1±0.1 =	0.4±0.3 =	0.2±0.2 =	+ =	+ =	0.1±0.1 =	0.2±0.2 =	+ =	+ >
18:3/18:3	14.2±0.4 >	11.2±1.5 >	7.0±1.0 =	7.6±0.8 >	5.8±0.3 =	5.7±0.2 =	6.0±0.4 =	6.1±0.6 >	16.5±0.6 >	13.0±2.4 >	10.3±0.8 =	10.9±0.4 <	14.0±2.8 =	12.5±1.1 =	13.1±0.9 =	12.1±1.5 >
20:5/16:1	1.4±0.7 <	3.7±0.7 =	4.0±0.5 >	2.6±0.6 =	2.4±0.6 >	1.4±0.6 =	1.7±0.5 >	1.0±0.3 =	0.5±0.2 <	2.1±0.5 =	1.9±0.6 =	1.7±0.3 =	1.4±0.4 =	1.0±0.6 =	0.5±0.1 =	0.4±0.3 =
18:3/18:2	4.1±0.6 >	2.9±0.1 =	2.6±0.4 =	3.1±0.1 >	1.8±0.2 >	1.4±0.3 =	1.3±0.1 >	0.6±0.2 >	4.7±1.3 =	3.4±1.0 =	3.8±0.6 =	3.4±0.5 <	5.2±0.6 =	4.7±1.1 >	3.2±0.2 >	1.7±0.4 >
20:4/16:1	0.1±0.0 =	0.1±0.1 <	0.2±0.0 >	0.1±0.0 =	+ =	+ =	+ =	+ =	0.2±0.1 <	0.5±0.2 >	0.1±0.1 =	0.2±0.1 =	0.1±0.1 =	0.1±0.0 =	0.1±0.1 =	0.1±0.0 >
20:5/16:0	1.6±0.2 =	1.7±0.2 =	1.8±0.5 =	2.0±0.3 >	0.9±0.4 =	0.9±0.1 =	0.9±0.2 =	0.7±0.3 >	0.5±0.1 =	0.6±0.2 =	0.7±0.1 >	0.3±0.2 =	0.2±0.2 <	0.4±0.1 >	0.2±0.1 =	0.1±0.1 >
18:2/18:2	0.2±0.3 =	0.2±0.1 =	0.1±0.1 <	0.3±0.0 >	0.1±0.1 =	0.1±0.1 =	0.1±0.0 =	0.1±0.0 =	0.2±0.0 =	0.3±0.1 =	0.2±0.1 =	0.1±0.1 =	0.4±0.3 =	0.4±0.3 =	0.2±0.1 =	0.1±0.1 =
18:3/18:1	1.8±1.1 =	2.3±0.5 =	2.2±0.7 =	1.6±0.4 =	1.7±0.3 >	0.6±0.2 <	1.0±0.2 >	0.5±0.1 >	1.9±0.6 >	0.7±0.2 =	0.5±0.2 =	0.6±0.2 =	0.6±0.3 <	0.9±0.1 =	0.7±0.5 =	0.4±0.1 >
20:4/16:0	- #	- #	- #	- #	- #	- #	- #	- #	0.1±0.1 =	0.2±0.2 =	0.3±0.2 >	0.1±0.1 =	0.2±0.1 =	0.1±0.1 =	0.1±0.1 =	+ >
16:0/20:4	+ =	+ >	- <	+ =	+ =	+ <	+ >	+ >	0.3±0.1 >	+ <	0.1±0.0 =	0.1±0.0 =	+ =	+ =	0.1±0.0 >	+ >
18:2/18:1	0.1±0.0 =	0.2±0.1 =	0.3±0.1 =	0.4±0.2 >	0.1±0.1 =	+ =	0.1±0.1 =	+ =	0.2±0.1 =	0.2±0.0 >	0.1±0.0 =	0.1±0.1 =	0.1±0.0 <	0.2±0.1 >	+ =	0.1±0.0 >
20:3/16:0	0.2±0.0 =	0.2±0.1 =	0.1±0.1 =	0.2±0.0 >	0.1±0.0 =	0.1±0.0 =	+ =	+ >	0.1±0.1 =	0.1±0.0 =	0.1±0.0 =	+ <	0.1±0.1 =	0.1±0.1 =	0.1±0.0 =	+ >
16:0/20:3	- <	+ <	0.1±0.0 =	0.1±0.0 =	0.1±0.0 =	0.1±0.1 =	0.1±0.1 =	+ <	0.1±0.0 =	0.1±0.0 =	0.2±0.2 =	0.2±0.1 =	0.2±0.1 =	0.1±0.1 =	0.2±0.1 =	0.3±0.2 =
18:0/18:3	0.1±0.1 =	0.1±0.0 =	0.1±0.1 =	0.1±0.0 =	0.1±0.0 >	+ =	+ =	0.1±0.1 =	0.4±0.4 =	0.1±0.0 =	0.2±0.1 =	0.3±0.1 =	0.2±0.1 <	0.4±0.1 =	0.4±0.3 =	0.4±0.3 =
18:1/18:1	0.2±0.1 =	0.1±0.1 =	0.2±0.2 =	0.3±0.2 =	0.1±0.0 =	0.1±0.1 =	0.1±0.1 =	+ >	0.1±0.0 =	0.1±0.1 =	0.1±0.1 =	+ =	+ <	0.1±0.0 >	+ =	+ =
18:0/18:2	- #	- <	+ =	0.1±0.1 >	+ <	+ =	+ =	+ <	0.1±0.0 =	0.2±0.3 =	+ =	+ =	0.1±0.1 =	0.1±0.1 =	0.1±0.2 =	+ =
18:0/18:1	0.1±0.0 =	0.1±0.0 =	0.2±0.2 =	0.2±0.1 =	0.2±0.0 >	0.1±0.0 =	0.1±0.1 =	+ =	0.1±0.1 =	0.2±0.1 >	0.1±0.0 =	0.1±0.1 =	0.1±0.0 =	0.1±0.0 >	+ =	0.1±0.1 =
18:2/19:3	0.1±0.1 =	0.1±0.1 =	0.1±0.0 >	+ =	+ =	+ =	+ =	0.1±0.0 =	0.5±0.3 =	0.4±0.3 =	0.2±0.1 =	0.4±0.3 =	0.2±0.2 =	0.1±0.1 =	0.1±0.0 =	0.2±0.2 =
18:3/19:1	- <	0.1±0.1 >	- <	+ =	+ >	+ =	+ =	+ =	0.2±0.2 =	0.1±0.0 =	0.1±0.1 =	0.1±0.0 =	0.1±0.0 <	0.1±0.0 >	0.1±0.0 =	0.1±0.0 =
20:5/18:4	27.3±1.3 =	29.3±1.9 =	31.4±1.9 =	33.1±1.2 <	37.1±1.2 <	39.3±0.9 =	39.8±2.8 =	42.1±0.5 <	27.9±2.5 <	34.8±3.9 =	33.7±3.0 =	33.9±1.2 =	31.5±3.7 =	30.5±2.8 =	33.0±3.4 <	37.0±1.7 <
20:5/18:3	19.7±1.1 =	18.6±0.7 =	18.2±1.0 =	16.5±1.7 <	20.0±0.4 <	22.3±0.7 =	22.2±0.9 <	23.8±1.0 <	14.6±1.6 =	14.2±1.4 =	11.8±2.8 =	11.5±0.3 <	17.6±2.6 =	19.4±5.5 =	19.3±3.8 =	21.6±1.2 <
20:4/18:4	3.3±0.4 <	4.6±0.6 <	5.5±0.3 =	6.4±1.2 >	5.2±0.1 =	5.0±0.5 >	4.1±0.3 =	3.3±0.9 =	3.5±0.4 =	3.7±0.5 <	6.2±0.4 =	6.8±2.1 =	4.4±2.2 =	4.5±2.1 =	4.5±0.9 >	2.4±0.6 >
20:4/18:3	2.2±0.0 =	1.8±0.6 <	3.4±1.3 =	4.2±0.8 >	2.5±0.3 <	4.0±0.3 =	2.8±2.0 =	2.0±0.9 =	2.1±0.6 >	1.3±0.4 =	1.4±0.6 =	1.9±0.6 <	4.3±0.8 =	4.1±0.7 >	2.4±0.1 >	1.3±0.2 >
20:5/18:2	2.2±0.3 =	2.2±0.7 =	2.9±0.5 >	1.6±0.3 <	3.4±0.3 >	1.0±0.4 <	2.9±0.4 >	1.2±0.2 >	0.8±0.2 =	1.1±0.3 =	1.3±0.4 =	1.2±0.2 >	0.7±0.3 =	0.5±0.1 <	0.9±0.2 <	1.6±0.3 <
20:3/18:3	0.1±0.0 =	0.1±0.1 =	0.1±0.0 =	0.1±0.1 =	0.1±0.0 =	+ =	+ =	0.1±0.1 =	0.3±0.2 >	+ =	0.1±0.0 =	0.1±0.1 =	0.1±0.0 =	0.1±0.1 =	+ =	+ >
20:4/18:2	0.2±0.1 =	0.3±0.2 =	0.4±0.1 =	0.5±0.3 =	0.4±0.1 >	0.3±0.0 =	0.4±0.3 =	0.1±0.1 =	0.2±0.0 =	0.2±0.1 >	0.1±0.0 =	0.1±0.1 <	0.4±0.2 >	0.1±0.0 <	0.2±0.0 >	0.1±0.1 >
20:5/18:1	1.0±0.2 <	2.0±0.1 >	0.7±0.2 <	2.2±0.2 <	3.1±0.3 =	2.7±0.5 >	1.3±0.1 <	2.1±0.3 <	0.7±0.4 =	0.3±0.1 =	0.2±0.1 =	0.2±0.1 =	0.3±0.1 =	0.2±0.2 =	0.2±0.1 =	0.2±0.1 >
18:3/20:2	0.2±0.2 =	0.1±0.0 =	0.1±0.0 =	+ =	+ =	+ =	+ =	+ =	0.1±0.1 =	+ =	+ =	0.1±0.0 >	+ <	0.1±0.0 =	+ =	+ >
20:4/18:1	0.1±0.2 =	0.1±0.1 =	0.2±0.1 =	0.2±0.1 <	0.4±0.2 >	0.2±0.1 =	0.4±0.2 =	0.1±0.0 =	0.4±0.2 >	0.1±0.0 =	0.1±0.0 =	0.1±0.1 =	0.1±0.1 =	+ =	0.1±0.1 =	+ >

20:3/18:1	0.1±0.0 =	+ =	+ =	+ =	+ =	+ >	+ =	+	=		0.1±0.0 =	+ =	+ =	0.1±0.0 =	+ =	+ =	+ <	0.1±0.0 =
18:1/20:2	- <	0.1±0.1 =	+ =	+ =	+ >	+ =	+ >	-	#		0.1±0.0 =	0.1±0.0 =	+ =	+ >	- <	+ =	+ =	+ >

SQDG	*Streblonema corymbiferum*										*Streblonema* sp.								
	0	5	12	20	50	100	150	200	T	#	0	5	12	20	50	100	150	200	T
14:0/14:0	0.3±0.1 =	0.4±0.3 =	0.3±0.3 =	0.5±0.4 =	0.3±0.2 =	0.4±0.2 =	0.5±0.0 =	0.3±0.1	=		0.4±0.2 =	0.5±0.2 =	0.3±0.2 =	0.4±0.1 >	0.2±0.1 <	0.4±0.2 =	0.3±0.0 >	0.1±0.1	>
14:0/16:1	0.7±0.0 <	1.3±0.3 =	0.9±0.5 =	0.6±0.1 <	1.1±0.3 =	1.2±0.2 =	1.2±0.3 >	0.7±0.3	=		0.6±0.2 =	0.4±0.3 <	0.9±0.2 =	0.6±0.2 =	0.5±0.1 <	0.7±0.1 =	0.7±0.3 >	0.3±0.2	=
14:0/16:0	2.6±0.4 <	4.6±1.2 =	4.1±1.1 =	5.1±0.8 >	3.3±0.2 =	2.9±0.4 =	3.2±1.3 >	1.7±0.5	>		6.1±1.2 >	4.1±1.2 <	6.8±0.7 >	5.6±0.7 >	3.9±0.6 =	3.5±1.2 =	4.7±0.8 =	3.7±0.9	>
16:1/15:0	0.2±0.0 >	0.1±0.1 =	0.3±0.2 =	0.2±0.1 =	0.2±0.1 =	0.3±0.2 >	0.1±0.0 <	0.3±0.2	=		0.2±0.1 =	0.3±0.4 =	0.2±0.0 =	0.2±0.1 =	0.2±0.2 =	0.2±0.1 =	0.1±0.1 =	0.1±0.1	=
16:0/15:0	- #	+ <	0.1±0.1 =	0.1±0.1 =	0.1±0.1 =	+ =	0.1±0.0 =	0.1±0.0	#		0.1±0.1 =	0.2±0.2 =	0.1±0.2 =	0.1±0.0 =	0.1±0.1 =	0.1±0.1 =	0.2±0.1 >	0.1±0.0	=
14:0/18:3	1.7±0.8 =	2.3±0.1 =	2.6±0.9 =	2.1±0.4 =	2.5±0.6 =	2.6±0.3 =	2.8±0.6 =	2.2±0.2	=		0.4±0.1 <	1.2±0.6 =	1.6±0.8 =	1.7±0.2 =	1.4±0.4 =	1.1±0.4 =	1.2±0.3 =	1.2±0.5	<
14:0/18:2	2.0±0.4 <	2.7±0.1 <	4.6±0.4 =	4.7±1.1 =	5.6±0.7 >	4.5±0.8 =	5.0±1.6 >	2.9±0.6	<		0.2±0.0 <	2.6±0.3 <	4.2±1.1 =	3.7±0.7 =	4.4±0.6 =	3.8±0.7 =	3.7±0.7 >	2.2±0.4	<
14:0/18:1, 16:0/16:1, 16:1/16:0	18.8±1.2 =	20.4±1.5 =	18.9±1.6 >	16.2±1.1 <	20.2±0.5 =	19.8±1.2 =	18.3±1.3 =	17.8±1.0	=		8.7±0.6 >	6.5±1.1 <	9.3±0.7 =	8.7±1.0 =	7.9±0.6 >	6.0±1.4 <	7.8±0.8 =	6.6±1.0	>
16:0/16:0	0.3±0.1 <	0.5±0.1 <	1.0±0.4 =	0.5±0.2 >	0.3±0.1 =	0.2±0.2 =	0.4±0.2 =	0.4±0.1	=		3.9±0.6 <	5.6±0.7 >	4.0±0.5 =	3.8±0.4 =	3.8±0.5 =	4.0±0.6 =	4.0±0.2 =	4.4±0.4	=
15:0/18:3	0.2±0.1 =	0.1±0.1 =	0.1±0.1 =	0.1±0.0 =	0.1±0.1 =	0.2±0.1 >	0.1±0.0 <	0.2±0.1	=		0.1±0.1 =	0.1±0.0 =	0.1±0.1 =	0.2±0.1 =	0.1±0.0 =	0.2±0.1 =	0.1±0.1 =	0.2±0.1	=
15:0/18:2	0.4±0.3 >	0.1±0.1 =	0.2±0.1 =	0.2±0.1 =	0.2±0.1 =	0.1±0.0 <	0.3±0.2 =	0.2±0.1	=		0.3±0.2 =	0.1±0.2 =	0.2±0.0 =	0.2±0.1 <	0.6±0.2 >	0.3±0.2 =	0.3±0.1 =	0.3±0.1	=
15:0/18:1	- #	0.1±0.1 =	0.4±0.3 =	0.5±0.3 =	0.4±0.2 >	- <	0.4±0.0 =	0.3±0.0	#		+ =	0.1±0.1 <	0.4±0.0 =	0.4±0.0 =	0.3±0.2 =	0.1±0.0 >	- <	0.1±0.1	=
17:1/16:0	0.4±0.0 =	0.4±0.2 =	0.3±0.3 =	0.5±0.5 =	0.4±0.4 =	0.8±0.2 =	0.5±0.3 =	0.6±0.2	<		0.4±0.2 =	0.2±0.1 =	0.5±0.4 =	0.3±0.1 =	0.2±0.1 <	0.5±0.0 =	0.4±0.2 >	0.2±0.0	=
14:0/20:5	0.1±0.0 >	+ >	- <	0.1±0.0 >	+ <	+ =	+ =	+	>		0.1±0.0 =	+ <	0.1±0.0 >	+ >	- <	+ =	+ =	0.1±0.0	=
16:1/18:3	+ =	0.1±0.0 =	0.1±0.0 =	+ =	0.1±0.1 =	+ =	0.1±0.1 >	-	>		0.1±0.0 =	0.2±0.3 =	0.1±0.1 =	+ >	+ <	0.1±0.1 =	+ =	+	>
20:4/14:0	- #	0.1±0.0 =	0.1±0.0 =	0.2±0.1 >	+ >	+ =	0.1±0.1 =	+	#		0.2±0.0 =	0.2±0.2 =	+ =	0.1±0.1 =	0.1±0.0 =	0.1±0.1 =	+ =	0.1±0.0	>
14:0/20:4	- #	0.2±0.0 >	- <	0.1±0.0 =	+ =	0.1±0.0 =	0.1±0.0 =	0.1±0.0	#		0.1±0.0 =	+ =	0.1±0.0 =	0.1±0.1 =	+ =	+ >	- <	0.1±0.0	=
18:3/16:0	12.6±0.7 >	11.5±0.7 =	9.2±2.2 =	10.7±1.0 =	9.9±1.6 =	11.7±0.9 =	11.0±1.1 =	13.1±2.9	=		24.1±2.3 =	23.8±1.1 >	22.3±0.8 =	22.5±2.0 >	14.5±1.8 =	14.7±1.6 =	15.6±1.2 =	15.1±0.5	>
18:2/16:0	7.9±0.3 =	7.0±1.1 =	7.8±0.9 =	7.7±1.9 =	7.5±1.0 >	6.0±0.4 =	6.2±0.6 >	4.4±0.3	>		16.2±1.3 =	15.7±1.3 >	14.0±1.0 =	14.6±1.3 <	17.6±1.4 =	15.8±1.2 >	13.6±1.2 >	10.4±0.6	>
18:1/16:0	43.4±1.4 >	40.9±1.6 =	40.3±2.1 =	41.5±1.8 =	40.0±1.2 <	42.4±1.8 =	42.6±4.8 =	46.6±4.0	=		32.4±4.1 =	31.9±2.5 =	29.2±1.9 =	31.6±4.2 <	37.7±2.3 <	42.1±3.3 =	41.4±3.2 <	49.8±3.1	<
18:1/16:0	3.7±0.0 =	3.2±0.7 =	3.9±0.9 =	3.2±0.8 =	2.5±0.9 =	2.5±0.4 =	2.8±1.2 =	3.5±1.4	=		2.0±0.9 =	2.2±0.6 =	2.8±0.2 =	2.7±0.1 =	2.5±0.5 =	2.4±0.2 =	1.9±0.7 =	2.0±0.6	=
18:0/16:0	0.6±0.5 =	0.4±0.5 =	0.4±0.5 =	0.1±0.1 =	0.1±0.1 =	0.1±0.0 =	+ <	0.2±0.1	=		0.1±0.1 =	0.3±0.3 =	0.1±0.1 =	0.1±0.1 =	0.1±0.0 =	0.2±0.2 =	0.2±0.1 =	0.1±0.0	=
16:0/19:1	0.3±0.1 =	0.2±0.2 =	0.2±0.3 =	0.4±0.4 =	0.3±0.1 =	0.3±0.2 =	0.3±0.3 =	0.4±0.3	=		0.2±0.2 =	0.2±0.2 =	0.1±0.0 =	0.1±0.1 =	0.2±0.2 =	0.1±0.0 <	0.3±0.2 >	+	=
17:0/18:1	- #	0.1±0.0 =	0.2±0.1 =	0.1±0.0 =	0.1±0.0 >	+ <	0.2±0.0 =	0.1±0.1	#		- #	- <	0.1±0.0 =	0.1±0.0 >	- #	- #	- #	-	#
18:4/18:4	0.5±0.3 >	0.1±0.0 =	0.1±0.1 =	0.1±0.1 =	0.1±0.1 =	+ =	0.1±0.1 =	0.1±0.1	>		0.1±0.1 =	+ =	+ =	+ =	0.1±0.1 =	0.1±0.1 =	+ =	0.1±0.0	=
18:3/18:4	0.3±0.2 =	0.3±0.1 =	0.3±0.1 =	0.2±0.1 =	0.3±0.1 =	0.2±0.1 =	0.3±0.2 =	0.3±0.1	=		0.4±0.2 =	0.8±0.8 =	0.3±0.2 =	0.2±0.2 =	0.4±0.3 =	0.4±0.1 =	0.4±0.3 =	0.4±0.1	=
18:3/18:3	0.3±0.0 =	0.5±0.3 =	0.4±0.3 =	0.6±0.2 =	0.6±0.1 =	0.7±0.1 =	0.8±0.1 =	0.6±0.2	<		0.7±0.5 =	0.4±0.0 <	0.6±0.1 =	0.5±0.1 =	0.4±0.2 =	0.7±0.5 =	0.6±0.1 =	0.4±0.1	=
18:2/18:4	0.1±0.1 =	0.1±0.1 =	0.1±0.0 =	0.1±0.0 >	0.1±0.0 =	0.1±0.1 =	0.1±0.2 =	0.1±0.1	=		0.4±0.1 =	0.3±0.0 =	0.1±0.1 =	0.2±0.2 =	0.2±0.1 =	0.1±0.1 =	0.1±0.1 =	0.1±0.0	>
18:3/18:2	0.3±0.2 >	0.1±0.0 <	1.0±0.0 >	0.1±0.0 =	+ <	0.1±0.0 >	- <	0.1±0.0	>		0.1±0.0 <	1.5±0.0 >	+ =	0.1±0.1 >	- <	0.1±0.0 <	0.4±0.0 >	0.1±0.1	=
18:2/18:3	0.4±0.2 =	0.3±0.1 =	0.3±0.0 <	1.0±0.1 =	1.0±0.3 >	0.5±0.2 =	0.7±0.3 =	0.5±0.2	=		0.2±0.1 =	0.7±0.2 =	0.6±0.1 =	0.4±0.3 <	1.3±0.2 =	1.2±0.2 >	0.8±0.2 =	0.6±0.2	<
18:1/18:4	- #	0.1±0.0 =	0.2±0.0 >	+ =	0.1±0.0 =	+ >	- #	-	#		0.1±0.0 >	- <	0.1±0.0 =	0.1±0.1 =	0.1±0.0 >	+ <	0.1±0.0 >	-	>
20:5/16:0	0.4±0.1 >	0.1±0.0 <	0.2±0.1 =	0.2±0.1 =	0.1±0.0 =	0.1±0.1 >	+ =	+	>		0.1±0.1 =	0.1±0.1 =	0.1±0.1 =	0.1±0.0 =	0.1±0.1 =	+ =	0.1±0.0 =	+	=
18:3/18:1	0.1±0.0 <	0.6±0.3 >	0.2±0.1 <	0.6±0.1 =	0.6±0.7 =	0.7±0.2 =	0.5±0.4 =	0.5±0.3	<		0.1±0.1 =	0.3±0.2 =	0.2±0.2 =	+ =	0.1±0.1 =	0.3±0.3 =	0.3±0.1 =	0.2±0.1	=
18:0/18:4	0.1±0.0 =	0.1±0.0 <	0.2±0.0 >	0.1±0.0 =	+ =	0.1±0.0 =	0.1±0.1 =	+	>		0.2±0.0 =	0.1±0.0 =	+ <	0.1±0.0 =	0.1±0.0 =	+ =	+ =	+	>
18:2/18:1	0.1±0.1 =	0.2±0.1 =	0.1±0.1 =	0.2±0.0 =	0.1±0.2 =	0.1±0.1 =	0.3±0.3 =	0.2±0.2	=		0.1±0.1 =	0.1±0.1 =	0.1±0.0 =	0.2±0.2 =	0.1±0.0 =	0.1±0.0 =	0.1±0.1 =	0.1±0.0	=
20:3/16:0	0.1±0.1 =	0.2±0.1 =	0.1±0.1 =	0.1±0.1 >	+ =	0.1±0.0 =	0.1±0.0 <	0.2±0.0	=		0.2±0.1 =	0.2±0.2 =	0.1±0.1 =	0.1±0.1 >	+ <	0.1±0.1 >	+ <	0.1±0.0	=
16:0/20:3	0.1±0.0 >	+ <	0.1±0.0 =	0.2±0.1 =	0.1±0.1 =	0.1±0.1 =	0.1±0.0 =	0.2±0.0	=		0.2±0.1 =	0.2±0.1 =	0.1±0.0 =	0.1±0.2 =	+ <	0.2±0.1 >	0.1±0.0 >	0.1±0.0	>
18:1/18:1	0.3±0.0 =	0.3±0.0 =	0.1±0.0 =	0.3±0.2 =	0.4±0.2 >	0.2±0.1 =	0.3±0.2 =	0.4±0.2	=		0.1±0.1 =	0.2±0.2 =	0.1±0.1 <	0.2±0.0 =	0.2±0.1 =	0.3±0.3 =	0.2±0.0 =	0.5±0.0	<
20:2/16:0	0.2±0.1 =	0.1±0.0 =	0.3±0.2 <	0.5±0.1 =	0.3±0.2 =	0.3±0.2 =	0.3±0.1 =	0.3±0.2	=		0.1±0.1 =	0.2±0.2 =	0.1±0.0 =	0.1±0.0 =	0.1±0.0 =	0.1±0.0 =	0.1±0.0 =	0.1±0.1	=

18:0/18:2	0.4±0.2	=	0.2±0.0	<	0.5±0.2	=	0.5±0.4	=	0.4±0.1	>	0.1±0.0	=	0.2±0.1	=	0.1±0.1	>	0.1±0.1	=	0.1±0.0	=	0.1±0.0	=	0.1±0.1	<	0.4±0.2	>	0.1±0.1	=	0.1±0.0	=	0.1±0.1	=
18:0/18:1	+	<	0.1±0.0	<	0.2±0.1	=	0.1±0.1	<	0.3±0.0	=	0.2±0.1	=	0.2±0.1	=	0.3±0.3	=	0.1±0.0	=	0.1±0.0	=	0.1±0.0	=	+	<	0.1±0.0	>	+	=	0.1±0.0	=	0.1±0.0	=
16:0/20:0	0.1±0.0	=	0.1±0.0	=	0.1±0.1	=	0.1±0.1	=	+	=	+	=	0.1±0.1	=	0.2±0.2	=	0.2±0.1	=	+	=	+	=	+	<	0.1±0.0	=	0.1±0.0	=	+	=	+	=
20:5/18:4	0.2±0.0	>	0.1±0.0	=	0.1±0.0	=	0.1±0.1	=	0.2±0.0	>	0.1±0.1	=	0.2±0.1	=	0.2±0.1	=	-	<	0.1±0.1	=	0.1±0.1	=	0.2±0.1	=	0.2±0.1	=	0.2±0.2	=	0.1±0.0	=	0.2±0.2	<
20:5/18:3	0.1±0.1	=	0.1±0.0	=	0.1±0.0	=	0.1±0.0	=	0.1±0.1	=	+	=	0.1±0.1	=	0.2±0.1	=	0.4±0.0	>	+	=	0.1±0.0	>	+	=	+	=	+	=	+	=	0.1±0.0	>
20:4/18:4	0.1±0.0	=	0.1±0.0	=	0.1±0.0	=	+	=	+	=	0.1±0.0	=	+	>	-	>	0.3±0.0	>	+	=	0.1±0.0	=	0.1±0.1	=	0.1±0.0	=	+	=	+	=	+	>
20:5/20:5	-	#	-	<	0.1±0.0	>	-	#	-	<	+	>	-	<	+	#	-	#	-	<	+	=	+	=	-	<	+	>	-	#	-	#
20:5/20:4	-	#	-	<	+	=	0.1±0.0	>	-	<	+	>	-	#	-	#	0.3±0.0	>	-	<	+	>	+	<	0.1±0.0	>	+	=	+	=	+	>
20:4/20:4	-	#	0.1±0.0	>	-	<	0.1±0.0	>	-	<	+	>	-	<	0.1±0.0	#	0.1±0.0	>	-	<	0.1±0.0	>	+	<	0.1±0.0	>	-	#	-	#	-	>
16:0/24:0	0.1±0.0	=	0.1±0.1	=	0.1±0.1	=	0.1±0.0	=	0.1±0.1	=	+	=	+	=	0.1±0.2	=	0.1±0.0	>	+	=	+	=	+	=	0.1±0.1	=	0.1±0.0	=	+	<	0.2±0.1	=

PG	*Streblonema corymbiferum*																*Streblonema* sp.															
	0		5		12		20		50		100		150		200	T #	0		5		12		20		50		100		150		200	T
16:1/15:0	0.2±0.1	=	0.2±0.1	>	0.1±0.0	=	+	<	0.1±0.0	=	0.1±0.1	=	0.1±0.0	<	0.2±0.1	=	0.2±0.1	>	+	<	+	>	+	=	0.1±0.1	=	0.1±0.0	=	+	<	0.4±0.1	<
16:0/15:0	0.1±0.0	>	+	<	+	=	+	=	0.1±0.1	=	0.2±0.0	=	0.1±0.1	<	0.4±0.2	<	0.4±0.2	>	0.1±0.0	>	+	<	0.1±0.0	=	0.1±0.1	=	0.2±0.1	<	0.8±0.2	>	0.4±0.0	=
18:3/14:0	0.4±0.1	<	0.6±0.2	>	0.3±0.2	=	0.3±0.2	=	0.3±0.1	=	0.3±0.2	=	0.3±0.3	=	0.2±0.1	=	0.5±0.4	=	0.2±0.0	=	0.1±0.1	=	0.2±0.1	=	0.1±0.1	=	0.1±0.1	=	0.2±0.0	>	0.1±0.0	>
14:0/18:2	0.2±0.1	=	0.1±0.1	=	0.2±0.1	=	0.2±0.0	=	0.4±0.2	=	0.2±0.1	=	0.3±0.3	=	0.1±0.1	=	0.7±0.1	>	0.4±0.1	>	0.1±0.0	=	0.1±0.1	=	0.1±0.1	=	+	=	0.1±0.0	<	0.2±0.0	>
14:0/18:1	0.2±0.2	<	0.5±0.1	>	0.2±0.1	=	0.2±0.0	<	0.4±0.1	=	0.4±0.2	=	0.4±0.3	=	0.2±0.1	=	0.4±0.2	=	0.3±0.1	>	0.1±0.1	=	0.2±0.1	=	0.2±0.1	=	0.1±0.1	=	0.2±0.0	>	0.1±0.0	>
16:0/16:1	0.1±0.1	=	0.2±0.2	=	+	=	0.1±0.1	=	+	=	0.1±0.0	=	0.3±0.2	=	0.2±0.1	=	0.2±0.1	>	+	=	0.1±0.1	=	0.1±0.1	=	0.2±0.1	=	0.1±0.0	=	0.2±0.2	=	0.3±0.1	=
17:0/16:1	0.1±0.0	>	+	=	0.1±0.0	=	+	<	0.1±0.0	=	+	=	0.1±0.0	=	0.1±0.1	=	0.1±0.0	=	0.1±0.0	=	+	=	+	<	0.1±0.0	=	0.1±0.1	<	0.3±0.0	>	0.1±0.0	=
18:3/16:1	7.1±0.2	>	5.5±0.7	>	3.6±0.2	<	4.3±0.5	=	5.5±1.8	<	9.6±0.8	>	6.9±1.7	<	13.6±4.1	<	2.5±0.4	>	1.4±0.1	>	0.7±0.2	=	0.9±0.1	<	4.1±1.2	=	4.9±0.4	<	8.4±1.8	<	14.5±0.8	<
18:3/16:0	23.9±0.6	<	29.2±1.2	<	32.2±2.0	=	32.4±0.9	>	20.4±0.8	>	18.7±0.6	=	19.6±1.2	=	17.8±2.1	>	24.6±0.9	<	27.5±0.2	=	27.6±1.6	=	28.4±1.0	=	26.0±3.9	=	23.9±1.1	=	24.2±2.7	>	18.1±2.7	>
18:1/16:1	0.2±0.2	=	0.1±0.1	=	0.1±0.1	=	0.1±0.1	=	0.1±0.1	=	0.2±0.0	=	0.2±0.1	<	0.6±0.2	<	3.0±0.8	=	2.0±1.1	>	0.6±0.1	=	0.7±0.1	>	0.4±0.1	=	0.5±0.1	>	0.3±0.2	<	0.9±0.4	>
16:0/18:2, 18:2/16:0	5.3±0.5	>	4.7±0.2	<	5.6±0.4	=	5.6±0.2	=	5.6±0.3	>	4.7±0.3	=	5.1±0.6	=	5.2±0.2	=	5.7±2.4	=	5.8±0.6	=	4.8±0.7	=	5.0±0.7	<	7.6±0.4	<	8.1±0.2	=	7.1±2.0	=	6.0±1.1	=
16:0/18:1	4.4±1.3	=	4.0±0.1	>	3.4±0.5	=	4.0±0.6	<	5.9±0.6	<	6.7±0.6	=	6.3±1.2	<	9.7±1.0	<	6.6±0.9	<	8.3±0.9	=	8.5±0.7	=	9.2±0.6	<	13.7±1.7	=	15.8±2.4	=	17.0±1.9	<	26.6±1.3	<
17:0/18:3	0.1±0.1	=	+	=	+	=	+	=	+	=	0.1±0.0	>	+	<	0.1±0.1	=	0.2±0.1	>	+	=	+	=	+	<	0.1±0.1	>	+	<	0.1±0.0	<	0.2±0.0	=
17:0/18:1	0.2±0.1	=	0.2±0.1	=	0.1±0.1	=	0.2±0.1	=	0.2±0.1	=	0.3±0.2	=	0.3±0.1	=	0.3±0.2	=	0.2±0.0	=	0.2±0.2	=	0.2±0.1	>	0.1±0.0	<	0.2±0.1	=	0.2±0.1	=	0.3±0.1	=	0.2±0.1	=
18:4/18:4	+	=	+	=	+	=	+	<	+	=	0.1±0.1	=	+	=	+	=	0.1±0.2	=	0.1±0.1	=	0.1±0.1	=	+	=	0.1±0.1	=	0.2±0.2	=	+	<	0.1±0.0	=
18:3/18:4	3.5±1.1	=	3.8±0.6	=	3.3±0.5	=	3.5±0.5	<	4.2±0.3	>	3.5±0.6	=	3.6±0.7	=	2.6±0.9	=	5.1±0.3	=	5.4±0.2	<	8.0±0.5	=	8.1±0.6	>	4.0±0.4	=	4.8±0.9	=	4.6±0.6	=	4.3±0.2	>
18:3/18:3	27.7±1.4	=	29.5±1.6	>	27.6±0.6	=	27.7±0.9	>	25.3±0.9	=	25.7±0.9	=	25.8±1.3	>	20.4±1.5	>	26.8±1.2	=	27.9±1.5	<	32.5±1.3	=	31.0±3.5	>	20.3±2.4	=	19.7±1.8	=	18.3±2.2	>	14.4±0.4	>
16:1/20:5	0.1±0.1	>	+	=	+	=	+	<	+	=	+	=	0.1±0.1	=	0.2±0.1	=	0.2±0.2	=	0.2±0.3	=	+	=	+	<	0.1±0.0	=	0.1±0.1	<	0.2±0.1	=	0.2±0.1	=
18:3/18:2	11.8±1.0	>	10.0±0.8	<	11.0±0.2	=	10.7±0.6	<	13.1±0.5	=	12.0±1.1	=	12.0±0.7	>	9.2±0.6	>	10.6±1.9	=	10.0±0.5	=	9.7±1.2	=	9.7±0.9	=	11.1±2.0	=	9.2±1.9	=	8.7±1.8	>	3.7±0.2	>
18:2/18:3	0.2±0.2	=	0.1±0.0	=	0.1±0.0	>	+	=	0.2±0.2	=	0.1±0.1	=	0.1±0.0	>	0.1±0.0	=	0.1±0.1	=	0.2±0.1	=	+	=	0.1±0.1	=	0.2±0.3	=	0.1±0.0	<	0.5±0.2	>	0.1±0.0	=
20:5/16:0	0.1±0.1	=	0.1±0.1	=	+	=	+	=	+	=	+	=	+	=	0.1±0.1	=	0.1±0.0	=	0.1±0.1	=	+	=	+	=	0.1±0.1	=	0.1±0.0	=	0.1±0.1	=	0.1±0.1	<
16:0/20:5	0.3±0.2	=	0.1±0.0	=	0.5±0.5	=	0.1±0.1	=	+	<	0.2±0.0	>	+	=	0.1±0.2	=	0.4±0.3	=	0.3±0.2	=	0.2±0.1	=	0.1±0.0	=	0.1±0.0	=	0.4±0.3	=	0.3±0.1	=	0.2±0.0	=
18:2/18:2	2.3±1.1	=	1.8±0.5	<	2.5±0.3	=	2.2±0.6	=	2.7±0.6	>	1.6±0.1	=	1.5±0.5	=	1.9±1.3	=	1.5±0.6	=	2.4±0.9	=	2.0±0.1	<	2.3±0.3	<	3.3±0.9	=	3.4±0.4	>	1.8±0.1	>	0.6±0.1	>
18:3/18:1	4.9±0.4	>	3.6±0.3	>	2.3±0.1	=	2.9±0.6	<	5.4±1.2	=	5.8±0.5	<	7.0±0.9	>	5.2±0.4	=	2.7±0.5	>	1.6±0.3	>	0.8±0.1	=	0.7±0.2	<	1.7±0.1	=	1.4±0.8	=	1.3±0.4	=	1.0±0.0	>
18:1/18:3	0.3±0.0	=	0.1±0.1	=	0.1±0.1	=	+	=	+	=	+	=	0.1±0.1	=	0.1±0.1	=	0.1±0.1	=	0.1±0.1	=	0.1±0.1	=	+	<	0.3±0.2	=	0.1±0.1	=	0.1±0.1	=	0.1±0.0	=
20:4/16:0	0.1±0.0	=	0.1±0.0	=	+	=	+	=	+	=	+	<	0.1±0.1	=	+	>	0.1±0.1	=	0.1±0.0	=	0.1±0.0	>	+	=	0.1±0.1	=	0.2±0.1	>	+	=	0.1±0.1	=
16:0/20:4	0.1±0.0	=	0.1±0.0	=	+	>	+	<	+	=	0.1±0.0	=	+	=	0.1±0.1	=	0.2±0.1	=	0.2±0.2	=	+	=	+	=	0.1±0.0	=	0.1±0.1	=	0.1±0.1	<	0.3±0.0	<
18:2/18:1	2.4±0.8	=	2.0±0.2	=	2.6±0.7	=	2.1±0.2	<	3.9±0.2	>	3.3±0.4	=	3.7±0.2	=	3.7±1.2	=	1.5±0.4	>	0.8±0.2	=	0.7±0.0	=	0.6±0.1	<	1.6±0.1	=	1.8±0.2	>	1.0±0.2	>	0.7±0.1	>
18:1/18:2	0.2±0.2	=	0.1±0.2	=	+	=	+	=	0.1±0.1	=	+	<	0.1±0.0	<	0.2±0.1	=	0.3±0.1	>	0.2±0.0	>	0.1±0.0	<	0.1±0.0	=	0.1±0.1	=	0.3±0.3	=	0.1±0.1	<	0.3±0.0	=
20:3/16:0	0.1±0.0	>	+	<	+	=	+	<	0.1±0.0	=	0.1±0.0	>	+	=	0.1±0.1	=	0.1±0.1	=	+	=	+	=	+	=	0.1±0.1	=	0.1±0.0	=	+	<	0.1±0.0	=
18:3/18:0	0.1±0.0	=	0.1±0.0	<	0.1±0.1	=	0.1±0.0	>	+	=	0.1±0.0	=	0.1±0.1	=	0.1±0.0	=	0.2±0.0	=	0.1±0.1	=	+	=	+	<	0.2±0.0	=	0.1±0.0	>	+	#	-	#
18:0/18:3	-	<	+	=	0.1±0.0	=	+	<	0.1±0.0	=	0.1±0.0	>	+	=	0.1±0.0	<	+	=	0.1±0.2	=	+	=	+	=	0.1±0.1	>	-	<	0.1±0.0	#	-	#

18:1/18:1	1.8±0.2	>	1.5±0.0	<	2.0±0.1	=	1.9±0.3	<	3.3±0.5	=	3.8±0.9	=	3.4±0.4	<	5.8±0.1	<		2.2±1.0	>	0.9±0.2	=	1.1±0.5	=	0.6±0.0	<	2.2±0.3	=	2.7±1.0	=	2.1±0.4	<	3.9±0.7	<
18:0/18:2	0.2±0.1	=	0.1±0.1	=	0.2±0.1	=	0.2±0.1	=	0.3±0.1	=	0.3±0.1	>	0.1±0.1	=	0.1±0.0	=		0.2±0.1	=	0.1±0.0	=	0.1±0.0	=	0.1±0.0	=	0.3±0.3	=	0.2±0.1	>	0.1±0.0	<	0.4±0.1	=
18:0/18:1	0.3±0.2	=	0.2±0.1	<	0.7±0.1	>	0.4±0.2	<	1.2±0.4	=	1.1±0.2	=	1.3±0.5	>	0.5±0.2	=		0.1±0.0	=	0.1±0.1	=	+	=	0.1±0.0	<	0.2±0.0	=	0.4±0.3	=	0.6±0.3	=	0.5±0.2	<
18:4/20:5	0.2±0.2	=	0.1±0.0	>	+	=	0.1±0.0	=	+	=	0.1±0.1	=	0.1±0.0	>	+	>		0.1±0.0	=	0.1±0.1	>	+	=	0.1±0.1	=	+	=	+	<	0.1±0.0	=	0.1±0.0	>
20:5/18:3	0.5±0.1	<	0.8±0.2	>	0.4±0.0	=	0.4±0.0	>	0.2±0.1	=	0.3±0.1	=	0.3±0.0	=	0.4±0.2	=		0.7±0.3	<	1.4±0.2	>	0.9±0.4	=	0.6±0.2	=	0.4±0.2	=	0.2±0.1	=	0.3±0.2	=	0.3±0.1	>
20:5/18:2	0.2±0.1	<	0.3±0.0	>	0.1±0.0	<	0.1±0.0	=	0.2±0.1	=	0.1±0.1	=	0.1±0.0	=	0.2±0.0	=		1.0±0.5	=	1.4±0.3	>	0.3±0.2	=	0.4±0.3	=	0.1±0.0	=	0.2±0.1	=	0.1±0.1	=	0.2±0.1	>
20:5/18:2	0.1±0.1	=	+	>	+	=	+	=	+	=	0.1±0.0	=	+	=	+	=		-	=	+	=	+	=	+	<	+	=	+	=	+	#	-	#
18:2/20:5	0.2±0.1	>	+	>	-	#	-	#	-	<	0.1±0.0	=	+	=	0.1±0.0	=		0.1±0.0	=	+	=	+	=	+	=	0.1±0.1	>	-	<	0.1±0.1	=	0.1±0.0	=
18:1/20:5	0.3±0.1	>	+	=	0.1±0.1	=	+	=	0.1±0.1	=	0.1±0.0	=	0.1±0.2	=	0.1±0.0	>		0.1±0.0	=	+	>	+	=	+	<	0.1±0.0	=	+	=	0.1±0.1	=	0.1±0.0	=
20:5/20:5	0.1±0.0	>	+	<	+	>	+	=	+	>	-	<	0.1±0.0	=	0.1±0.1	=		0.1±0.1	=	0.1±0.0	>	+	=	+	=	0.1±0.0	=	+	<	0.1±0.0	<	0.3±0.0	<
20:4/20:5	0.1±0.0	=	+	=	0.1±0.0	=	+	=	+	=	0.1±0.0	=	0.1±0.0	=	0.1±0.0	=		0.3±0.3	=	+	>	-	=	+	=	0.3±0.4	=	0.1±0.0	=	0.3±0.2	=	0.2±0.0	=

PE	*Streblonema corymbiferum*																	*Streblonema* sp.															
	0		5		12		20		50		100		150		200	T	#	0		5		12		20		50		100		150		200	T
16:1/16:1	0.3±0.1	=	0.5±0.1	=	0.3±0.4	=	0.3±0.2	=	0.2±0.1	>	+	=	0.1±0.0	<	0.2±0.1	=		1.0±0.6	=	0.9±0.5	=	1.0±1.2	=	0.3±0.2	=	0.3±0.1	=	0.3±0.2	=	0.2±0.2	=	0.3±0.2	>
16:0/16:1	0.4±0.5	=	0.1±0.0	=	0.1±0.1	=	+	<	0.1±0.0	=	0.1±0.1	=	0.1±0.0	<	0.1±0.0	=		1.4±0.6	>	0.2±0.2	=	0.1±0.1	=	+	=	0.1±0.1	=	0.1±0.0	<	0.2±0.1	>	0.1±0.0	>
14:0/20:5	1.8±0.4	>	1.0±0.3	=	0.5±0.4	=	0.7±0.2	<	1.6±0.4	=	2.0±0.6	=	1.4±0.5	=	2.0±1.4	=		1.6±1.2	=	0.9±0.4	<	1.8±0.3	=	1.4±0.6	>	0.5±0.3	=	0.4±0.1	=	0.6±0.2	=	0.5±0.2	=
14:0/20:4	7.6±0.5	>	4.8±0.5	>	3.0±0.5	=	3.6±0.4	=	4.1±0.9	=	3.6±0.2	=	3.6±0.1	>	3.4±0.1	>		1.0±0.7	=	1.5±0.2	>	0.9±0.3	<	1.6±0.5	>	0.6±0.1	<	0.8±0.1	=	0.8±0.2	>	0.4±0.1	=
18:1/16:1	5.5±1.5	=	3.8±1.2	>	1.7±0.8	=	1.5±0.6	>	0.5±0.3	=	0.2±0.1	=	0.1±0.0	<	0.3±0.2	>		26.3±1.6	>	23.5±1.9	>	16.2±0.4	>	12.1±2.3	>	3.1±0.3	=	3.7±0.6	>	1.0±0.2	=	0.7±0.1	>
16:0/18:1	0.4±0.1	=	0.5±0.4	=	0.3±0.0	=	0.3±0.2	=	0.2±0.0	=	0.2±0.1	=	0.3±0.1	=	0.3±0.2	=		1.5±0.8	=	1.2±0.1	=	1.0±0.6	=	0.8±0.1	>	0.3±0.2	=	0.5±0.2	=	0.7±0.5	=	0.4±0.2	>
18:0/16:0	0.5±0.0	>	+	=	+	=	+	=	+	=	+	=	0.1±0.0	=	0.1±0.0	>		0.2±0.2	=	0.1±0.1	=	+	=	+	<	0.1±0.0	=	0.1±0.1	<	0.4±0.0	>	0.1±0.1	=
20:5/15:0	0.1±0.0	=	+	<	0.1±0.0	=	0.1±0.0	=	0.1±0.0	=	0.1±0.0	=	+	=	+	>		0.4±0.2	>	0.1±0.0	=	0.1±0.1	=	0.1±0.0	=	+	=	+	=	0.5±0.6	=	0.1±0.0	>
20:4/15:0	0.1±0.0	>	0.1±0.0	=	0.2±0.1	=	+	=	+	<	0.1±0.0	=	0.1±0.1	=	+	>		0.2±0.1	=	0.1±0.1	=	+	=	+	<	0.1±0.0	>	0.1±0.0	<	0.4±0.3	=	0.1±0.1	=
18:3/18:4	0.9±0.2	>	0.2±0.2	=	+	=	+	=	+	=	+	=	0.1±0.1	=	+	>		+	=	0.1±0.0	=	+	=	0.1±0.0	>	-	<	+	>	-	<	+	=
18:4/18:3	0.7±0.5	=	0.3±0.2	=	0.2±0.1	=	0.3±0.2	>	+	<	0.1±0.0	=	0.1±0.1	>	+	>		0.3±0.1	>	0.1±0.0	=	0.1±0.1	=	+	=	0.1±0.0	=	+	>	-	<	0.1±0.1	>
16:0/20:5	1.0±0.8	=	1.1±0.3	>	0.6±0.2	=	0.7±0.5	<	2.1±0.1	>	1.3±0.6	=	1.2±0.5	<	3.5±0.7	<		3.5±0.9	<	6.4±0.3	=	6.2±1.1	=	6.3±0.7	>	2.8±0.1	=	2.3±1.1	=	3.5±0.8	=	4.3±0.1	=
18:2/18:2	0.3±0.2	=	0.3±0.1	=	0.2±0.1	=	0.1±0.1	=	0.1±0.0	=	+	=	0.1±0.0	=	0.1±0.0	=		1.0±1.3	=	0.2±0.1	=	0.2±0.1	>	+	<	0.1±0.0	=	0.1±0.0	=	0.2±0.1	=	0.1±0.1	=
16:0/20:4	4.6±0.7	=	5.0±0.8	=	4.3±0.6	=	4.5±0.1	>	4.1±0.4	<	4.7±0.3	>	3.3±0.3	<	4.8±0.5	=		12.0±2.3	>	7.9±0.2	=	7.1±0.9	=	5.9±1.0	=	5.5±0.2	=	6.2±1.0	=	6.7±0.8	=	6.5±1.1	>
18:2/18:1	0.7±0.2	>	0.3±0.2	=	0.3±0.2	=	0.3±0.1	=	0.3±0.1	=	0.3±0.2	=	0.3±0.1	=	0.4±0.2	>		0.5±0.1	=	0.3±0.2	=	0.2±0.1	=	0.2±0.1	>	0.1±0.0	<	0.2±0.1	=	0.3±0.1	=	0.4±0.3	=
18:1/18:1	1.1±0.4	=	1.1±0.4	=	0.9±0.5	=	1.0±0.3	<	1.8±0.4	=	1.8±0.7	=	1.9±0.2	=	1.7±0.8	=		0.7±0.2	<	1.3±0.2	=	1.1±0.5	=	0.9±0.2	>	0.5±0.0	<	1.1±0.1	>	0.9±0.0	=	1.0±0.4	=
20:5/18:4	4.9±2.5	=	4.1±0.4	>	2.7±0.6	=	2.2±0.9	>	0.6±0.4	=	0.3±0.2	=	0.4±0.2	=	0.3±0.2	>		1.5±0.4	>	0.9±0.4	=	0.7±0.1	>	0.3±0.1	>	0.1±0.0	=	0.1±0.0	<	0.3±0.0	=	0.1±0.1	>
20:5/18:3	1.4±0.8	=	0.8±0.3	=	0.5±0.2	=	0.4±0.2	=	0.4±0.2	=	0.4±0.3	<	0.8±0.2	>	0.2±0.2	>		0.6±0.3	=	0.5±0.5	=	0.3±0.3	=	0.4±0.1	>	0.2±0.2	=	0.2±0.1	=	0.2±0.0	=	0.1±0.1	>
18:3/20:5	6.5±0.7	=	5.9±0.6	>	4.1±0.1	=	4.1±0.5	>	0.8±0.2	>	0.3±0.1	<	0.7±0.2	=	0.4±0.3	>		0.9±0.3	=	0.8±0.5	=	0.4±0.0	=	0.5±0.2	>	0.3±0.0	>	0.1±0.0	<	0.3±0.2	>	0.1±0.0	>
18:3/20:4	1.4±0.4	=	1.3±0.7	=	0.8±0.3	=	0.6±0.2	<	1.4±0.4	=	1.2±0.8	=	1.1±0.4	=	0.9±0.1	>		0.4±0.2	=	0.6±0.2	=	0.5±0.2	=	0.7±0.2	>	0.2±0.1	=	0.5±0.4	=	0.3±0.0	=	0.3±0.2	=
18:2/20:5	0.3±0.3	=	0.1±0.1	=	0.1±0.0	=	0.1±0.1	=	0.1±0.1	>	+	<	0.1±0.0	=	0.1±0.0	>		0.6±0.8	=	0.1±0.0	>	0.1±0.0	=	0.1±0.0	=	0.1±0.1	=	0.1±0.1	<	0.3±0.2	>	0.1±0.0	=
18:2/20:4	1.9±1.5	=	0.9±0.7	=	0.5±0.3	=	0.6±0.5	=	0.6±0.4	=	0.6±0.2	=	0.6±0.5	=	0.3±0.1	>		0.6±0.3	=	0.3±0.3	=	0.3±0.1	=	0.2±0.1	=	0.3±0.1	=	0.3±0.1	=	0.4±0.1	=	0.2±0.1	=
18:1/20:5	1.1±1.0	=	1.0±0.4	=	0.4±0.4	=	0.5±0.1	<	0.9±0.2	=	0.8±0.3	=	0.8±0.6	=	0.4±0.3	=		0.7±0.3	>	0.3±0.3	=	0.2±0.1	=	0.3±0.1	=	0.2±0.1	=	0.3±0.1	=	0.5±0.5	=	0.2±0.1	>
18:1/20:5	0.7±0.8	=	0.4±0.2	=	0.5±0.2	=	0.6±0.2	=	0.5±0.4	=	0.5±0.2	=	0.4±0.2	=	0.5±0.2	=		0.5±0.5	=	0.4±0.4	=	0.3±0.2	=	0.3±0.1	>	0.1±0.0	=	0.2±0.1	=	0.2±0.1	=	0.2±0.1	=
18:1/20:4	0.9±0.4	<	2.1±0.6	=	1.9±0.7	=	2.1±1.2	>	0.7±0.1	<	1.2±0.2	=	1.0±0.4	>	0.3±0.2	>		1.0±0.8	=	0.3±0.2	=	0.5±0.1	>	0.2±0.1	<	0.3±0.1	=	0.5±0.2	=	0.9±0.4	>	0.2±0.1	=
18:0/20:5	0.5±0.2	=	0.4±0.2	=	0.7±0.2	=	0.9±0.3	=	0.6±0.2	<	1.4±0.2	=	1.1±0.4	=	1.7±0.8	<		2.3±2.1	=	1.2±0.9	=	1.0±0.5	=	1.3±0.2	=	0.9±0.5	=	1.2±0.4	=	1.3±0.9	=	1.6±0.5	=
18:0/20:4	2.7±0.7	=	3.1±0.1	<	3.5±0.4	=	4.0±0.5	=	3.8±0.2	>	2.7±0.1	<	3.4±0.4	=	2.3±1.0	=		1.5±1.0	<	2.6±0.2	>	2.2±0.3	=	2.4±0.5	<	4.0±0.1	<	5.1±0.8	=	4.6±0.2	=	4.4±0.5	<
20:5/20:5	2.9±1.0	=	3.0±0.5	>	2.2±0.3	=	2.3±0.4	<	2.9±0.4	<	4.7±0.3	>	3.1±1.0	<	6.6±2.1	<		2.3±1.6	<	8.6±0.9	=	9.2±1.2	=	8.8±0.2	>	5.9±0.4	=	5.6±0.9	<	7.2±0.6	=	7.7±0.7	<
20:4/20:5	10.5±1.0	<	12.4±1.5	=	13.8±0.8	=	14.2±1.1	<	18.9±0.5	<	23.1±1.1	=	22.2±2.0	<	25.3±0.8	<		12.7±0.3	<	17.0±1.6	<	21.3±0.3	=	22.9±2.4	<	26.0±0.8	=	24.0±2.6	=	27.0±2.7	=	28.6±0.3	<
20:5/20:3	2.0±0.4	>	0.6±0.2	>	0.3±0.2	=	0.1±0.0	=	0.3±0.2	=	0.3±0.2	=	0.2±0.1	=	0.2±0.0	>		0.9±0.5	>	0.4±0.1	=	0.3±0.1	=	0.3±0.1	=	0.2±0.1	=	0.3±0.1	=	0.2±0.3	=	0.1±0.0	>
20:3/20:5	1.2±0.6	>	0.3±0.1	=	0.2±0.0	=	0.2±0.1	=	0.2±0.1	=	0.2±0.1	=	0.2±0.2	=	0.3±0.3	>		1.9±0.4	>	0.6±0.8	=	0.2±0.1	=	0.2±0.2	=	0.1±0.0	<	0.2±0.1	=	0.6±0.9	=	0.2±0.0	>

20:4/20:4	20.0±1.4	<	27.9±2.3	<	39.5±4.5	=	38.4±1.8	=	40.4±1.6	>	35.1±1.5	=	37.7±3.1	>	30.2±1.5	<	13.4±1.2	=	15.3±2.3	<	20.5±0.2	<	26.0±0.8	<	41.9±1.7	>	37.8±2.2	>	34.3±1.9	=	35.0±1.1	<
20:3/20:4	1.3±0.0	>	0.7±0.1	=	0.6±0.3	=	0.4±0.2	=	0.2±0.1	<	0.3±0.1	=	0.2±0.1	=	0.3±0.0	>	0.3±0.2	=	0.1±0.0	=	0.3±0.2	=	0.3±0.2	=	0.1±0.0	<	0.4±0.2	=	0.2±0.2	=	0.2±0.3	=
20:4/20:2	0.8±0.1	=	0.7±0.2	>	0.2±0.1	=	0.4±0.3	=	0.2±0.1	=	0.1±0.1	=	0.1±0.0	=	0.2±0.2	>	0.8±0.8	=	0.1±0.0	=	0.1±0.0	>	+	<	0.1±0.0	=	0.1±0.0	=	0.2±0.2	=	0.1±0.1	=
20:1/20:5	0.6±0.1	=	0.7±0.3	=	0.7±0.6	=	0.5±0.3	=	0.5±0.1	=	0.6±0.2	=	1.0±0.3	=	0.6±0.2	=	0.2±0.2	=	0.2±0.1	=	0.2±0.1	=	0.2±0.0	=	0.1±0.1	=	0.1±0.0	=	0.2±0.1	=	+	=
20:1/20:4, 20:0/20:5	4.1±0.8	=	4.4±0.2	=	4.1±0.6	=	4.6±0.4	=	4.1±0.5	=	4.0±0.4	=	3.8±0.9	=	4.9±1.4	=	0.8±0.6	<	1.9±0.5	=	1.9±0.0	=	1.7±0.5	>	1.0±0.4	<	2.2±0.9	>	1.1±0.3	=	1.4±0.3	=
20:0/20:4	4.8±1.0	=	5.9±0.2	=	6.3±1.1	=	6.6±0.9	>	4.3±0.7	=	4.6±0.3	=	5.2±1.1	>	3.7±0.6	=	2.7±0.3	>	1.8±0.5	=	1.7±0.5	=	1.6±0.3	=	2.0±0.6	<	3.1±0.2	=	3.1±0.6	>	2.1±0.5	>
20:4/22:1	0.1±0.2	=	0.1±0.1	=	0.1±0.1	=	0.1±0.1	>	+	=	0.1±0.1	=	0.1±0.1	=	0.1±0.1	=	0.3±0.1	>	+	=	0.2±0.2	=	0.1±0.1	=	+	=	0.3±0.4	=	0.3±0.1	=	0.2±0.1	=
22:0/20:5	1.0±0.6	=	1.0±0.2	=	0.9±0.3	=	0.6±0.2	=	0.6±0.2	=	1.0±0.3	=	1.1±0.5	=	1.4±0.3	=	0.3±0.1	=	0.2±0.2	=	0.3±0.1	=	0.4±0.1	>	0.2±0.1	=	0.2±0.2	=	0.2±0.1	=	0.3±0.2	=
22:0/20:4	2.2±0.6	<	2.8±0.3	=	2.7±0.6	=	1.8±1.1	=	1.8±0.3	=	1.8±0.4	=	2.4±0.5	=	1.7±0.7	=	0.9±0.3	>	0.2±0.1	=	0.3±0.2	<	0.5±0.1	=	0.6±0.1	=	0.8±0.6	=	0.4±0.3	<	0.8±0.0	=
24:0/20:5	0.2±0.3	=	0.1±0.1	=	0.1±0.0	=	+	=	+	=	0.1±0.0	=	0.1±0.0	=	0.1±0.0	=	0.8±0.8	=	0.2±0.1	=	0.2±0.2	=	0.3±0.1	=	0.3±0.1	=	0.1±0.1	=	0.3±0.3	=	0.5±0.4	=
24:0/20:4	0.1±0.0	=	0.2±0.2	>	+	=	0.1±0.1	=	0.1±0.1	=	0.1±0.1	=	0.1±0.0	=	0.1±0.0	=	0.3±0.2	=	0.8±0.6	=	0.5±0.2	=	0.4±0.3	=	0.1±0.1	=	0.6±0.6	=	0.3±0.2	=	0.4±0.4	=

PC	*Streblonema corymbiferum*																	*Streblonema* sp.															
	0		5		12		20		50		100		150		200	T	#	0		5		12		20		50		100		150		200	T
14:0/14:1	+	=	+	>	-	<	+	>	+	<	+	=	+	>	-	>		+	=	+	=	+	>	+	<	+	>	-	<	+	>	-	>
14:0/14:0	0.2±0.2	=	0.1±0.0	=	0.1±0.1	=	0.1±0.1	=	0.1±0.0	=	0.1±0.0	=	0.1±0.0	=	0.1±0.1	=		+	=	0.1±0.1	=	0.1±0.0	=	0.1±0.1	>	+	=	+	=	+	=	+	=
14:0/16:1	0.1±0.0	=	+	=	+	=	0.1±0.0	=	+	<	0.1±0.0	>	+	=	+	=		+	=	0.1±0.0	>	+	=	+	=	+	=	+	=	+	=	+	=
14:0/16:1	0.1±0.1	=	+	=	+	=	+	=	+	=	+	=	+	=	+	=		+	=	+	>	+	=	+	=	+	=	+	=	+	=	+	>
14:0/16:0	0.1±0.0	=	0.1±0.0	>	+	=	+	=	+	=	+	=	+	=	+	>		0.1±0.0	=	0.2±0.1	>	0.1±0.1	=	0.1±0.1	>	+	=	+	=	+	=	+	>
18:4/14:1	-	#	-	<	+	=	+	=	+	=	+	>	-	<	+	<		+	=	+	>	-	<	+	>	+	>	-	#	-	#	-	>
14:0/18:4	0.1±0.1	=	+	=	+	<	0.1±0.0	=	0.1±0.1	=	+	<	0.1±0.1	>	+	=		+	<	0.1±0.0	=	0.1±0.0	=	0.1±0.0	=	0.1±0.1	=	+	=	0.1±0.0	=	+	=
14:0/18:3	3.1±0.3	<	3.8±0.3	>	2.0±0.1	<	2.8±0.4	<	3.6±0.3	=	3.4±0.2	=	3.3±0.3	>	2.8±0.2	>		1.1±0.3	=	1.3±0.4	<	2.4±0.5	=	2.2±0.2	>	1.5±0.3	=	1.5±0.2	=	1.5±0.4	=	1.4±0.1	=
14:0/18:3	0.5±0.1	>	0.3±0.1	=	0.4±0.2	=	0.2±0.1	=	0.2±0.1	=	0.3±0.2	=	0.6±0.3	=	0.4±0.2	=		0.2±0.1	=	0.2±0.1	=	0.3±0.3	=	0.1±0.1	=	0.1±0.1	=	0.1±0.1	=	0.2±0.1	=	0.1±0.0	>
14:0/18:2	3.7±0.1	<	5.5±0.6	>	3.4±0.1	<	4.2±0.3	=	4.5±0.9	=	3.9±0.1	=	3.6±0.7	=	3.4±0.3	>		2.2±0.3	<	3.2±0.3	=	3.8±0.6	<	4.6±0.5	=	4.1±0.3	>	3.6±0.4	=	3.6±0.9	>	2.3±0.0	=
14:0/18:2	1.2±0.1	>	0.4±0.2	<	1.3±0.4	=	0.9±0.2	=	0.9±0.4	>	0.4±0.2	<	1.0±0.3	>	0.3±0.1	>		0.2±0.1	=	0.1±0.0	<	0.4±0.2	=	0.2±0.2	=	0.1±0.1	=	0.2±0.0	>	0.1±0.0	=	0.1±0.1	=
14:0/18:1	6.0±0.5	<	9.7±1.2	=	9.0±1.4	=	9.6±0.6	>	5.8±0.2	>	4.6±0.2	=	5.4±1.1	>	3.1±0.3	>		2.0±0.2	=	2.3±0.2	<	3.1±0.4	=	2.7±0.6	>	0.9±0.2	=	1.0±0.2	=	0.8±0.1	>	0.5±0.1	>
16:0/16:0	+	=	+	=	+	=	+	=	+	=	+	=	+	=	+	=		0.1±0.1	=	+	>	+	=	+	=	+	=	+	=	+	>	+	=
17:3/16:1	0.1±0.0	>	+	=	+	=	0.1±0.1	=	0.1±0.0	=	+	=	0.1±0.0	=	+	>		+	=	+	=	0.1±0.0	>	+	=	+	=	+	=	+	=	+	=
15:0/18:2	0.1±0.1	=	+	=	+	=	+	=	+	=	+	<	+	=	+	=		0.1±0.0	>	+	=	+	=	+	=	0.1±0.1	=	+	=	0.1±0.1	=	+	>
15:0/18:1	0.1±0.0	=	0.2±0.1	=	0.2±0.1	=	0.3±0.1	>	0.1±0.1	=	0.2±0.1	>	0.1±0.0	<	0.1±0.0	=		0.1±0.1	=	0.1±0.0	>	+	<	0.1±0.0	>	+	=	+	=	+	=	+	>
14:0/20:5	11.0±0.7	>	7.5±0.5	>	2.2±0.3	<	6.6±0.5	<	9.9±0.8	<	14.5±2.3	=	14.8±0.3	<	19.3±1.6	<		8.8±0.4	<	11.7±1.6	=	10.3±1.4	=	10.2±1.4	=	9.8±0.4	=	9.6±1.0	=	10.4±0.5	=	10.1±0.4	<
14:0/20:4	4.4±0.2	>	1.7±0.1	>	0.4±0.3	=	0.6±0.1	<	1.0±0.2	=	1.0±0.0	=	0.9±0.6	=	1.0±0.3	>		1.9±1.0	=	0.9±0.1	=	1.1±0.2	=	0.9±0.3	>	0.5±0.2	=	0.6±0.2	=	0.4±0.1	>	0.2±0.1	>
14:0/20:4	7.3±0.5	<	8.8±1.0	=	8.0±0.3	=	7.8±0.8	<	12.8±0.8	>	11.3±1.0	=	10.4±1.0	>	8.7±1.2	<		3.9±0.2	<	4.5±0.3	<	4.9±0.3	=	5.5±0.7	=	5.7±0.4	=	5.9±0.5	=	5.4±0.5	>	4.7±0.3	<
14:0/20:3	0.3±0.2	=	0.4±0.4	=	0.2±0.0	<	0.5±0.3	>	0.2±0.1	=	0.3±0.2	=	0.5±0.1	=	0.3±0.2	=		0.2±0.2	=	0.2±0.2	=	0.3±0.1	>	0.2±0.1	=	0.4±0.1	=	0.2±0.1	=	0.2±0.1	=	0.3±0.1	=
16:0/18:3	2.3±1.0	<	3.5±0.2	>	2.3±0.5	=	2.3±0.3	=	2.8±0.4	>	2.3±0.3	=	2.4±0.2	=	2.4±0.3	=		1.8±0.3	<	3.5±0.5	=	3.3±0.8	=	3.2±0.2	>	2.5±0.2	=	2.6±0.1	=	2.5±0.2	=	2.3±0.3	<
18:0/16:3	0.5±0.2	=	0.3±0.2	=	0.3±0.2	=	0.3±0.1	=	0.4±0.2	>	0.1±0.1	<	0.4±0.2	>	0.2±0.1	>		0.1±0.1	=	0.2±0.1	=	0.3±0.2	=	0.1±0.1	=	0.1±0.1	=	0.1±0.1	=	0.4±0.3	=	0.1±0.1	=
16:0/18:2	4.1±0.5	=	3.9±0.4	=	4.0±0.5	=	3.9±0.2	>	2.7±0.3	>	2.4±0.2	=	2.7±0.3	>	1.9±0.2	>		4.7±0.8	<	5.6±0.5	=	5.8±0.3	=	5.7±0.3	=	5.8±0.5	=	5.2±0.4	=	5.4±0.7	>	4.3±0.5	=
16:0/18:1	4.1±0.4	=	3.9±0.6	<	6.9±0.6	>	6.2±0.2	>	2.5±0.1	=	2.4±0.2	=	2.6±0.4	>	1.7±0.0	>		4.6±0.4	=	4.7±0.2	=	4.5±0.2	=	4.5±1.1	>	1.7±0.3	=	1.9±0.7	>	1.2±0.2	>	0.9±0.0	>
18:4/17:2	0.1±0.1	=	0.1±0.1	=	0.1±0.0	=	+	<	0.1±0.0	=	0.1±0.0	=	0.2±0.1	=	0.2±0.1	=		0.1±0.1	=	0.1±0.0	=	0.1±0.1	=	0.1±0.0	=	0.2±0.1	>	0.1±0.0	=	0.1±0.1	=	0.1±0.0	=
17:2/18:3	1.5±0.1	>	0.8±0.1	>	0.4±0.2	=	0.2±0.1	>	0.1±0.0	=	0.1±0.0	=	0.1±0.0	=	+	>		0.3±0.2	=	0.1±0.0	=	+	=	+	=	+	=	+	=	+	=	+	>
15:0/20:5	0.2±0.2	=	0.3±0.1	=	0.1±0.1	=	0.1±0.1	=	0.2±0.1	=	0.3±0.0	=	0.4±0.3	=	0.6±0.3	=		0.3±0.1	=	0.2±0.1	=	0.2±0.2	=	0.2±0.1	=	0.4±0.1	>	0.2±0.1	=	0.3±0.1	=	0.2±0.1	=
17:1/18:3	0.1±0.1	=	0.1±0.0	=	0.1±0.0	=	0.1±0.0	=	0.1±0.0	=	+	<	0.1±0.0	=	0.1±0.0	=		0.1±0.1	=	0.1±0.1	=	0.1±0.1	=	0.2±0.1	=	0.2±0.1	=	0.1±0.0	=	0.1±0.1	=	0.1±0.0	=
15:0/20:4	0.1±0.0	=	0.2±0.1	>	0.1±0.0	=	0.1±0.0	<	0.2±0.1	=	0.2±0.1	=	0.1±0.0	<	0.2±0.0	=		0.1±0.1	=	0.1±0.0	=	0.1±0.0	=	0.1±0.1	=	0.1±0.1	=	0.1±0.0	>	0.1±0.0	=	0.1±0.0	=
15:0/20:4	0.1±0.1	=	0.1±0.1	=	0.1±0.0	=	+	=	0.1±0.1	=	0.1±0.0	=	0.1±0.0	=	0.1±0.1	=		0.1±0.0	=	0.1±0.2	=	0.1±0.1	=	0.1±0.1	=	0.1±0.1	=	0.1±0.1	=	0.1±0.0	=	0.1±0.0	=
17:1/18:2	+	=	+	=	+	=	+	=	+	=	+	=	+	>	+	=		+	=	+	>	+	=	+	<	0.1±0.0	>	+	=	+	=	+	>

17:1/18:2	+ =	+ =	0.1±0.0 =	0.1±0.1 =	+ =	+ =	+ <	0.1±0.0 =	0.1±0.2 =	0.1±0.0 =	+ <	0.1±0.0 =	0.1±0.1 =	0.1±0.0 =	0.1±0.0 >	+ =
17:0/18:2	0.1±0.0 =	0.1±0.0 =	0.1±0.1 <	0.2±0.1 >	0.1±0.0 =	0.1±0.0 =	+ =	+ =	0.2±0.0 >	0.1±0.0 =	0.2±0.1 =	0.1±0.0 >	+ =	0.1±0.0 =	+ =	+ >
16:0/19:1	+ =	+ =	+ >	+ <	+ =	+ =	+ =	+ >	0.1±0.0 =	+ =	+ =	+ =	+ =	+ =	+ =	+ =
18:3/18:3	0.2±0.1 =	0.2±0.1 >	0.1±0.0 =	0.1±0.1 =	0.1±0.1 =	0.1±0.1 =	0.1±0.0 =	+ >	0.2±0.1 =	0.1±0.0 =	0.1±0.0 =	0.1±0.0 =	0.1±0.0 =	0.2±0.1 =	0.1±0.0 =	0.1±0.0 =
20:5/16:1	0.1±0.0 =	+ =	0.1±0.1 =	+ =	+ <	+ >	+ =	0.1±0.1 =	0.1±0.1 =	0.2±0.1 =	0.1±0.1 =	0.1±0.1 =	0.1±0.1 =	0.1±0.0 =	0.1±0.1 =	0.1±0.0 =
16:1/20:5	0.1±0.2 =	+ =	+ =	+ =	+ <	+ >	+ =	+ =	0.1±0.0 =	+ =	+ =	+ =	+ =	+ =	+ =	+ =
18:3/18:2	0.1±0.1 =	0.1±0.0 <	0.2±0.0 >	0.1±0.0 =	0.1±0.0 =	0.1±0.0 =	+ =	+ =	0.1±0.0 =	0.2±0.1 <	0.4±0.1 >	0.2±0.1 =	0.2±0.1 =	0.2±0.0 =	0.1±0.1 =	0.1±0.1 =
20:4/16:1	0.1±0.0 >	+ =	0.1±0.1 =	0.1±0.1 =	0.1±0.0 =	0.1±0.0 =	+ =	+ >	0.2±0.1 >	0.1±0.0 =	0.1±0.1 =	0.1±0.0 =	0.1±0.1 =	0.1±0.0 =	0.1±0.0 =	0.1±0.1 >
16:0/20:5	3.6±0.6 =	3.8±0.0 >	2.1±0.4 =	2.3±0.5 <	3.2±0.3 <	4.9±0.7 =	4.4±1.7 <	7.4±0.6 <	14.5±2.2 =	15.8±0.6 >	11.8±0.9 =	12.5±1.2 >	11.1±0.7 <	12.4±0.9 =	13.0±0.7 <	16.2±1.0 =
16:0/20:4	1.5±0.5 >	0.4±0.3 =	0.1±0.1 <	0.3±0.1 =	0.2±0.2 >	0.1±0.0 <	0.4±0.2 =	0.3±0.1 >	2.2±0.6 >	1.6±0.1 >	1.1±0.3 >	0.4±0.0 =	0.4±0.2 =	0.5±0.1 =	0.3±0.3 =	0.4±0.1 >
16:0/20:4	2.8±0.4 =	3.0±0.4 <	4.4±0.5 =	3.8±0.5 =	3.6±0.4 =	4.2±0.4 =	4.1±0.3 =	3.6±0.5 <	7.5±1.3 =	7.3±0.9 =	6.3±1.0 =	6.4±0.3 <	7.9±0.5 =	7.3±0.5 =	7.4±0.1 =	7.3±0.4 =
18:2/18:1	0.8±0.1 =	0.8±0.3 <	1.7±0.1 >	1.3±0.3 >	0.7±0.1 >	0.2±0.2 =	0.4±0.1 >	0.2±0.0 >	0.6±0.2 >	0.2±0.1 <	0.5±0.2 =	0.4±0.1 >	0.2±0.1 =	0.2±0.1 =	0.2±0.1 =	0.3±0.2 =
18:1/18:2	0.5±0.3 =	0.7±0.4 =	0.4±0.1 <	0.7±0.2 >	0.4±0.1 >	0.2±0.1 =	0.3±0.1 =	0.3±0.1 =	0.5±0.2 =	0.5±0.3 =	0.4±0.3 =	0.4±0.1 =	0.3±0.1 =	0.3±0.2 =	0.4±0.3 =	0.2±0.1 >
16:0/20:3	0.8±0.2 =	0.6±0.2 =	0.9±0.3 =	0.9±0.2 >	0.5±0.3 =	0.6±0.1 =	0.5±0.3 =	0.5±0.2 >	0.3±0.3 =	0.1±0.0 <	0.6±0.3 =	1.0±0.3 =	0.7±0.3 =	0.6±0.2 =	0.6±0.2 =	0.4±0.1 =
16:0/20:3	0.2±0.2 =	0.3±0.3 =	0.3±0.2 =	0.5±0.1 >	0.1±0.0 =	0.1±0.1 =	0.3±0.2 =	0.1±0.1 =	0.1±0.1 =	0.2±0.3 =	0.4±0.2 =	0.2±0.2 =	0.2±0.0 =	0.1±0.1 =	0.2±0.1 =	0.2±0.3 =
18:1/18:1	0.4±0.2 =	0.6±0.1 <	1.5±0.1 >	0.7±0.2 >	0.4±0.1 =	0.4±0.0 >	0.2±0.1 =	0.2±0.1 =	0.5±0.1 >	0.3±0.1 =	0.3±0.1 =	0.2±0.1 =	0.2±0.2 =	0.2±0.1 =	0.2±0.1 >	+ >
18:1/18:1	0.4±0.2 =	0.4±0.1 =	0.6±0.2 =	0.4±0.1 >	0.2±0.0 =	0.3±0.1 >	0.1±0.1 =	0.2±0.0 >	0.4±0.2 =	0.1±0.0 =	0.1±0.0 =	0.2±0.1 =	0.1±0.1 =	0.2±0.1 =	0.1±0.1 =	0.1±0.0 >
18:0/18:2	0.4±0.3 =	0.1±0.1 <	0.3±0.0 >	0.2±0.0 =	0.2±0.1 >	0.1±0.0 =	0.1±0.0 <	0.2±0.0 =	0.3±0.0 >	0.2±0.1 =	0.1±0.1 =	0.1±0.0 =	0.3±0.2 =	0.2±0.1 >	0.1±0.0 =	0.1±0.1 >
18:0/18:1	0.1±0.1 =	0.1±0.0 =	0.1±0.1 =	0.4±0.3 =	0.1±0.0 =	0.1±0.1 =	0.1±0.1 =	0.2±0.1 =	0.2±0.1 =	0.1±0.0 =	0.1±0.0 =	0.1±0.0 =	+ =	+ =	+ =	+ >
20:5/18:4	+ =	+ =	+ =	+ =	+ >	+ =	+ =	+ =	+ =	0.1±0.1 =	0.1±0.0 =	0.1±0.0 >	0.1±0.0 =	+ =	+ =	0.1±0.1 =
20:5/18:4	+ =	+ =	0.1±0.1 =	+ =	+ =	+ >	+ =	+ =	0.2±0.2 =	+ <	0.1±0.0 >	+ <	0.1±0.0 >	+ <	0.1±0.0 >	+ =
20:5/18:3	1.5±0.2 =	1.4±0.2 =	1.3±0.1 =	1.3±0.4 =	1.4±0.1 <	1.6±0.1 =	1.3±0.4 <	1.8±0.1 <	2.7±1.0 =	2.5±0.3 =	2.4±0.3 =	2.4±0.2 =	2.2±0.2 >	1.9±0.2 =	2.0±0.3 =	2.3±0.5 =
20:5/18:2	2.6±0.4 =	2.1±0.3 =	2.4±0.6 =	2.7±0.0 <	3.4±0.2 >	2.8±0.1 >	2.5±0.1 =	2.3±0.3 =	3.6±0.3 =	4.0±0.4 >	3.5±0.2 <	4.2±0.5 =	4.5±0.5 =	4.7±0.5 =	4.9±0.3 =	4.8±0.3 <
20:4/18:3	0.5±0.1 <	1.2±0.5 =	1.2±0.1 >	1.0±0.1 >	0.6±0.1 <	1.2±0.2 =	1.1±0.1 =	0.9±0.2 <	0.9±0.6 =	0.5±0.2 <	1.4±0.5 >	0.5±0.3 =	0.6±0.1 =	0.7±0.1 =	0.5±0.1 >	0.3±0.0 =
20:3/18:3	0.1±0.0 =	0.1±0.1 =	0.1±0.0 =	+ =	+ <	0.1±0.0 >	+ =	+ =	0.5±0.3 =	0.3±0.1 >	0.1±0.0 =	0.1±0.1 >	0.1±0.0 <	0.2±0.0 =	0.1±0.1 =	0.1±0.1 >
20:4/18:2	1.3±0.5 <	4.1±1.0 <	5.5±0.7 =	5.4±0.7 >	3.0±0.2 =	2.6±0.8 =	2.0±0.6 >	0.9±0.2 =	1.7±0.5 =	2.0±0.3 =	2.5±0.7 =	2.5±0.9 <	3.7±0.5 =	3.4±0.8 =	3.1±0.5 >	2.0±0.4 =
20:5/18:1	3.4±0.8 >	2.1±0.8 =	2.4±0.5 >	1.6±0.4 <	2.4±0.2 >	1.5±0.3 <	2.2±0.1 =	2.2±0.1 >	2.0±0.3 >	1.5±0.3 =	1.9±0.2 <	2.4±0.3 >	1.2±0.1 =	1.5±0.5 =	1.0±0.2 <	1.8±0.3 =
18:2/20:3	0.3±0.1 >	0.2±0.0 <	0.4±0.1 =	0.3±0.1 =	0.1±0.1 =	0.1±0.1 =	0.1±0.1 =	0.1±0.1 >	0.5±0.3 =	0.2±0.2 =	0.2±0.1 =	0.2±0.1 >	+ <	0.2±0.1 =	0.1±0.0 <	0.2±0.0 >
20:4/18:1	4.8±0.2 <	7.8±1.6 <	12.4±1.4 =	11.2±1.1 >	4.9±0.3 >	3.4±0.3 =	4.3±1.0 >	2.3±0.7 >	2.7±0.9 >	1.6±0.1 <	2.1±0.1 =	1.9±0.2 >	0.9±0.0 <	1.2±0.2 >	0.8±0.2 =	0.9±0.1 >
18:0/20:5	0.2±0.1 =	0.3±0.2 =	0.3±0.2 =	0.3±0.2 =	0.2±0.1 =	0.2±0.0 <	0.4±0.1 <	0.5±0.0 <	0.9±0.2 =	0.7±0.0 =	0.6±0.1 =	0.5±0.2 =	0.3±0.2 <	1.0±0.1 =	0.8±0.2 =	1.0±0.0 =
18:0/20:4	0.1±0.0 >	0.1±0.0 =	0.1±0.1 =	0.2±0.0 =	0.2±0.1 =	0.2±0.1 =	0.3±0.1 =	0.2±0.1 =	0.3±0.4 =	0.1±0.1 =	0.2±0.1 =	0.2±0.1 =	0.2±0.1 =	0.2±0.1 =	0.2±0.1 <	0.3±0.1 =
18:1/20:1	+ =	+ =	+ >	+ =	+ =	+ =	+ =	+ =	0.1±0.0 =	+ =	+ =	+ =	+ =	+ =	+ =	+ >
18:2/20:0	+ >	+ =	+ =	+ >	+ <	+ =	+ <	+ =	+ =	+ >	+ >	+ =	+ =	+ >	- <	+ =
16:0/22:1	0.1±0.0 =	+ =	+ =	+ =	+ =	+ =	+ =	+ =	+ >	- <	+ =	+ =	+ <	+ >	+ =	+ =
18:1/20:0	+ =	+ =	+ =	+ =	+ =	+ =	+ >	+ =	0.1±0.0 =	+ >	+ =	+ =	+ =	+ =	+ =	+ >
20:4/19:2	0.1±0.1 =	0.1±0.1 =	0.1±0.0 =	0.2±0.1 =	0.1±0.0 =	0.1±0.0 =	0.1±0.0 =	+ =	0.2±0.0 =	0.2±0.1 >	0.1±0.0 =	0.1±0.0 =	0.1±0.0 =	0.1±0.0 =	0.1±0.0 =	0.1±0.0 >
20:4/19:1	0.1±0.0 =	0.1±0.0 =	0.1±0.0 =	0.1±0.1 >	+ =	+ =	+ =	+ >	0.1±0.1 =	0.2±0.2 >	+ =	+ =	+ =	+ >	+ <	0.1±0.0 =
20:5/20:5	2.3±0.3 =	2.0±0.2 >	1.4±0.3 =	1.4±0.3 <	2.3±0.5 <	4.2±0.1 =	3.3±1.2 <	7.9±1.5 <	5.1±1.3 =	6.1±0.4 >	5.5±0.4 =	5.9±0.9 =	5.7±0.5 =	6.4±1.1 =	7.3±0.9 <	8.4±0.5 <
20:5/20:4	1.0±0.3 >	0.3±0.2 =	+ =	0.1±0.1 =	0.1±0.1 =	+ <	0.2±0.1 =	0.3±0.1 >	1.2±0.6 >	0.3±0.3 =	0.3±0.3 =	0.3±0.1 =	0.1±0.2 =	0.2±0.1 =	0.3±0.2 =	0.2±0.3 >
20:4/20:5	6.3±0.9 =	5.9±1.4 =	5.7±0.5 >	4.8±0.4 <	9.9±0.4 <	12.2±1.1 >	10.0±1.4 <	12.8±0.4 <	8.7±1.1 =	8.0±0.9 =	9.0±0.9 =	8.3±1.8 <	13.6±1.0 =	13.2±0.6 <	14.8±1.2 =	15.7±1.7 <
20:5/20:3	1.7±0.4 >	0.5±0.2 >	0.1±0.0 =	0.2±0.2 =	0.3±0.1 <	0.5±0.2 =	0.6±0.1 >	0.2±0.1 >	0.9±0.6 =	0.3±0.3 =	0.3±0.0 =	0.3±0.1 =	0.2±0.1 =	0.3±0.1 =	0.2±0.1 =	0.2±0.1 >
20:3/20:5	0.8±0.4 =	0.5±0.2 =	0.4±0.3 =	0.4±0.3 =	0.5±0.1 =	0.5±0.1 =	0.5±0.1 =	0.7±0.5 =	0.7±0.5 =	0.3±0.3 =	0.2±0.2 =	0.4±0.3 =	0.6±0.2 =	0.7±0.1 =	0.4±0.3 =	0.5±0.2 =
20:4/20:4	5.8±0.9 =	4.8±0.3 <	7.6±0.7 =	6.8±1.0 <	9.5±0.5 >	6.9±1.1 =	7.7±2.2 >	4.8±1.0 =	4.0±0.3 >	3.0±0.2 <	3.9±0.3 =	4.2±1.0 <	7.6±0.2 =	6.8±1.4 =	5.9±0.4 =	6.0±0.4 <
20:4/20:3	1.8±0.3 <	2.1±0.1 =	2.4±0.4 >	1.6±0.3 =	1.6±0.2 =	1.4±0.2 =	1.6±0.2 >	1.0±0.3 >	0.4±0.1 <	0.5±0.0 <	0.9±0.2 =	0.8±0.0 <	1.1±0.1 =	0.8±0.4 =	0.6±0.0 <	0.7±0.1 <
20:4/20:2	0.6±0.4 =	0.3±0.1 >	0.1±0.0 =	0.2±0.2 =	0.2±0.0 >	0.1±0.0 >	0.1±0.0 =	0.1±0.1 =	0.1±0.1 =	0.1±0.0 =	0.1±0.0 =	0.1±0.1 =	0.1±0.1 =	0.1±0.1 =	0.1±0.0 =	0.1±0.0 =
20:4/20:2	0.3±0.2 =	0.3±0.1 =	0.5±0.5 =	0.3±0.1 >	0.1±0.0 =	0.2±0.1 >	0.1±0.0 =	0.1±0.1 =	0.1±0.1 =	0.1±0.2 =	+ =	+ =	0.1±0.0 =	+ =	+ >	+ =

20:5/20:1	0.1±0.0	=	0.1±0.0	=	0.1±0.1	>	+	=	+	=	+	<	0.1±0.0	>	+	>		+	=	+	=	+	=	+	=	+	=	+	=	+	=	+	=
20:4/20:1	0.1±0.1	=	0.1±0.0	=	0.1±0.0	>	+	=	0.1±0.0	=	0.1±0.0	=	0.1±0.0	=	+	>		+	=	+	=	+	=	+	<	+	>	+	=	+	>	-	>
20:1/20:4	0.1±0.0	=	+	<	0.1±0.0	>	+	=	0.1±0.0	=	+	=	+	=	+	=		0.1±0.0	>	+	<	+	=	+	=	+	=	+	=	+	>	-	>
20:4/20:1	0.1±0.0	=	0.1±0.0	=	0.1±0.0	=	0.1±0.0	=	0.1±0.0	=	0.1±0.0	=	0.1±0.0	=	+	=		0.1±0.1	=	+	=	0.1±0.0	<	0.1±0.0	>	+	<	0.1±0.0	=	0.1±0.1	=	+	=
20:0/20:4	0.1±0.1	=	+	<	0.1±0.0	>	+	=	0.1±0.0	=	0.1±0.1	=	+	=	+	=		0.1±0.1	=	0.1±0.1	=	+	=	+	=	+	=	+	=	+	=	+	>
20:5/22:6	0.1±0.1	=	0.1±0.0	=	+	=	+	=	+	=	+	=	+	=	0.1±0.0	=		0.1±0.1	=	+	=	+	=	+	<	0.1±0.1	=	0.1±0.0	=	+	=	0.1±0.1	=
20:4/22:4	0.1±0.0	=	0.1±0.0	>	+	=	+	=	+	=	+	=	+	=	+	=		0.1±0.0	>	+	=	+	=	+	=	+	=	+	=	+	=	+	>

PI	*Streblonema corymbiferum*																	*Streblonema* sp.															
	0		5		12		20		50		100		150		200	T	#	0		5		12		20		50		100		150		200	T
14:0/18:1, 16:0/16:1	7.3±2.3	=	8.8±2.2	=	6.2±2.9	=	8.1±1.2	=	6.3±3.7	=	5.0±2.6	=	4.2±1.2	=	5.7±3.0	=		3.8±0.9	=	4.2±2.9	=	4.0±0.8	=	3.2±1.1	=	2.3±1.1	=	1.6±0.6	<	4.0±0.2	=	3.6±1.2	=
16:0/18:4	1.7±0.9	=	1.3±0.3	>	0.7±0.5	=	1.1±0.6	=	1.1±0.1	=	1.0±1.1	=	1.2±1.1	=	1.2±0.8	=		1.3±0.9	=	0.4±0.1	=	0.5±0.1	>	0.2±0.1	<	0.5±0.1	=	0.2±0.0	=	0.5±0.2	#	1.3±0.0	#
16:0/18:3	8.1±2.2	=	5.2±5.0	=	4.4±0.1	=	5.2±1.4	=	3.7±1.5	<	5.4±0.6	=	5.6±0.5	=	4.1±2.1	>		4.8±2.7	=	4.8±2.3	=	3.6±2.5	=	3.0±0.5	<	4.1±0.0	=	4.0±2.0	=	4.7±0.9	=	4.3±2.3	=
16:0/18:2	12.9±4.9	=	10.5±2.9	=	9.1±1.8	=	7.3±2.9	<	10.6±1.5	=	10.4±1.1	=	9.6±0.8	=	8.3±1.4	=		18.1±1.5	=	18.9±3.1	=	15.2±2.4	=	16.8±0.5	=	16.2±1.6	=	18.0±1.8	>	14.9±1.2	=	12.2±2.7	>
16:0/18:1	53.8±6.8	=	59.2±1.6	=	58.7±2.6	=	57.5±3.1	<	61.5±1.0	<	67.1±4.6	=	68.3±0.7	=	69.9±3.6	<		63.0±0.8	<	68.1±3.4	<	74.3±3.6	=	74.4±1.0	=	73.0±1.6	=	73.6±1.4	=	73.6±0.5	=	77.3±4.1	<
16:0/18:0	7.9±2.7	=	7.4±1.7	<	10.3±1.8	>	5.7±1.2	>	3.0±0.8	<	4.7±0.8	=	3.3±2.7	=	2.8±1.6	>		1.3±0.9	=	0.7±0.4	=	0.5±0.2	=	0.4±0.2	=	0.3±0.1	=	0.3±0.0	=	0.2±0.1	=	0.4±0.4	=
16:0/20:5	3.2±1.4	>	1.1±0.3	>	0.5±0.1	<	0.7±0.1	=	1.2±0.1	=	0.8±0.5	=	0.8±0.6	=	3.6±4.4	=		5.5±2.3	>	1.6±0.4	>	0.9±0.3	=	0.9±0.3	=	1.8±1.8	=	1.1±0.7	=	0.7±0.2	=	0.6±0.3	>
18:3/18:0	1.3±0.5	>	0.4±0.2	=	0.9±0.7	=	0.3±0.1	=	0.6±0.5	=	0.2±0.0	<	0.8±0.4	=	1.4±1.3	=		2.3±0.0	>	0.7±0.4	>	0.2±0.0	=	0.2±0.1	<	0.4±0.1	=	0.5±0.3	=	0.5±0.2	=	0.7±0.1	>
18:0/18:1	3.8±1.4	=	6.2±3.0	<	10.8±2.1	<	14.3±2.2	=	12.7±2.9	>	5.4±1.4	=	6.1±1.1	>	3.5±1.9	=		1.1±0.1	>	0.6±0.5	=	0.8±0.3	=	1.0±0.4	=	1.6±1.0	=	1.3±0.4	=	1.1±0.8	=	0.7±0.5	=

PHEG	*Streblonema corymbiferum*																	*Streblonema* sp.															
	0		5		12		20		50		100		150		200	T	#	0		5		12		20		50		100		150		200	T
20:5/20:5	0.7±0.2	=	0.7±0.5	=	0.2±0.1	=	0.3±0.2	=	0.3±0.1	=	0.2±0.2	=	0.2±0.2	=	0.7±0.5	=		0.6±0.3	<	1.7±0.7	=	1.2±0.2	=	1.3±0.3	>	0.6±0.4	=	0.9±0.4	<	1.4±0.2	>	0.3±0.1	=
20:5/20:4	13.0±0.5	=	11.6±1.4	=	12.8±0.8	>	10.4±1.6	<	14.3±1.7	<	19.6±0.5	=	17.3±5.0	<	25.6±3.5	<		12.2±2.7	<	26.3±2.6	=	26.7±3.7	=	27.5±1.9	>	19.7±7.0	=	17.5±2.7	=	20.4±2.4	=	19.1±1.9	<
20:4/20:4	86.6±0.8	=	87.7±0.9	=	87.0±0.7	<	89.3±1.8	>	85.4±1.8	>	80.2±0.7	=	82.5±5.1	>	73.7±3.8	>		87.2±2.6	>	72.1±2.8	=	72.1±3.9	=	71.3±2.2	<	79.7±7.5	=	81.6±2.9	=	78.2±2.3	=	80.5±1.7	>

DGTS	*Streblonema corymbiferum*																	*Streblonema* sp.															
	0		5		12		20		50		100		150		200	T	#	0		5		12		20		50		100		150		200	T
14:0/14:0	0.1±0.1	=	+	=	+	=	+	<	0.1±0.0	>	+	=	+	<	+	=		+	<	0.1±0.0	=	+	=	0.1±0.1	=	+	=	0.1±0.0	=	+	=	0.1±0.1	=
14:0/16:1	0.1±0.0	=	0.1±0.1	=	0.1±0.0	=	0.2±0.1	=	0.3±0.1	=	0.2±0.0	=	0.4±0.3	=	0.6±0.1	<		+	=	+	<	0.1±0.1	=	0.2±0.0	=	0.3±0.2	=	0.2±0.2	=	0.3±0.2	=	0.4±0.2	<
15:0/15:0	0.4±0.1	>	0.2±0.1	>	0.1±0.0	=	0.2±0.1	<	4.2±0.6	=	3.2±0.9	=	3.5±1.7	=	4.0±1.0	<		0.1±0.0	<	0.1±0.0	<	0.3±0.1	=	0.3±0.2	=	0.6±0.4	=	0.6±0.3	<	1.4±0.7	>	0.6±0.2	<
14:0/16:0	0.2±0.1	>	0.1±0.0	=	0.1±0.1	=	0.1±0.1	<	0.2±0.1	=	0.1±0.1	=	0.2±0.1	=	0.2±0.1	=		0.1±0.1	=	0.2±0.1	=	0.2±0.0	=	0.2±0.1	=	0.4±0.3	=	0.3±0.3	=	0.3±0.1	=	0.2±0.1	<
15:0/16:0	0.1±0.1	=	0.2±0.0	=	0.1±0.1	=	0.2±0.2	<	1.3±0.2	<	1.8±0.4	=	2.2±0.8	=	1.6±0.9	<		+	=	0.1±0.1	=	0.3±0.0	=	0.3±0.3	=	0.2±0.1	=	0.3±0.1	=	0.4±0.3	=	0.3±0.2	<
14:0/18:3	0.1±0.0	=	0.1±0.0	=	+	=	+	=	+	=	+	=	+	=	+	>		+	=	+	<	0.1±0.0	=	0.1±0.1	=	+	=	+	=	+	=	+	=
16:1/16:1	0.5±0.1	>	0.3±0.1	=	0.2±0.1	=	0.3±0.2	=	0.3±0.2	=	0.2±0.1	=	0.2±0.1	=	0.2±0.1	>		0.1±0.0	=	0.1±0.1	<	0.3±0.0	=	0.3±0.1	=	0.3±0.1	=	0.2±0.2	=	0.3±0.1	=	0.4±0.0	<
14:0/18:1	0.7±0.8	=	0.4±0.2	=	0.5±0.3	=	0.5±0.5	=	0.8±0.3	=	1.1±0.5	=	0.7±0.3	=	1.0±0.6	=		1.9±0.7	=	2.6±0.3	=	2.9±0.6	=	2.7±0.3	=	2.6±1.9	=	2.1±1.3	=	2.1±0.6	=	2.6±0.5	=
16:1/16:0	0.9±0.5	=	0.4±0.4	=	0.3±0.2	=	0.2±0.3	=	0.6±0.3	=	0.4±0.1	=	0.8±0.4	=	0.7±0.4	=		0.7±0.5	=	0.3±0.1	<	0.5±0.2	=	0.8±0.5	=	1.7±1.4	=	1.1±1.4	=	1.9±1.9	=	0.5±0.3	=
15:0/17:0	+	=	+	=	+	>	+	>	+	=	+	=	+	=	+	>		+	=	+	=	+	=	+	=	+	=	+	=	+	<	0.1±0.0	<
16:0/16:0	0.5±0.2	=	0.5±0.4	=	0.3±0.1	>	0.1±0.1	=	0.2±0.1	=	0.2±0.1	=	0.2±0.1	=	0.2±0.0	>		0.5±0.1	=	0.4±0.4	=	0.6±0.2	=	0.5±0.2	=	0.5±0.2	=	0.4±0.1	=	0.5±0.1	=	0.4±0.0	=
15:1/18:1	0.2±0.0	=	0.2±0.2	=	0.1±0.1	=	+	<	0.1±0.0	=	+	=	+	>	+	>		+	=	+	=	+	=	+	=	+	=	0.1±0.0	=	0.1±0.0	=	0.1±0.0	<
17:1/16:1	0.1±0.0	=	0.1±0.1	=	0.1±0.0	=	+	=	+	=	+	=	+	=	+	>		+	=	+	<	+	>	+	<	+	=	+	=	+	<	+	<
15:0/18:1	0.1±0.1	=	0.1±0.1	=	0.2±0.1	=	+	<	0.2±0.1	=	0.2±0.1	=	0.3±0.1	=	0.2±0.1	=		0.1±0.1	=	+	=	+	=	+	=	0.2±0.1	>	+	=	0.1±0.1	=	0.1±0.1	=
17:1/16:0	0.1±0.0	=	0.1±0.1	<	0.2±0.0	>	0.1±0.1	=	0.1±0.1	=	0.1±0.1	=	0.1±0.1	=	0.1±0.0	=		0.1±0.1	<	0.1±0.0	>	0.1±0.1	=	0.1±0.1	=	0.3±0.2	=	0.3±0.2	=	0.1±0.1	=	0.1±0.1	=

16:1/18:4	0.1±0.0	>	+	=	+	=	+	=	+	=	+	=	+	=	+	>	+	=	+	=	+	=	+	=	+	=	+	=	+	=	+	>
18:3/16:1	0.1±0.1	=	+	>	-	<	+	=	+	=	+	=	+	=	+	>	-	<	+	<	0.1±0.0	=	0.1±0.0	=	+	=	+	=	+	=	+	<
16:2/18:2	0.1±0.1	=	+	=	0.1±0.1	=	+	=	+	=	+	>	+	=	+	=	+	>	+	<	+	=	+	=	+	=	+	=	+	=	+	=
18:4/16:0	0.4±0.3	=	0.2±0.1	=	0.1±0.0	=	+	=	+	=	+	>	+	=	+	>	+	>	+	=	0.1±0.1	=	+	=	+	=	+	=	+	=	+	>
16:1/18:2	0.1±0.1	=	0.1±0.0	=	0.1±0.0	=	0.1±0.1	=	+	<	0.1±0.1	=	+	=	0.1±0.0	=	+	=	+	=	0.1±0.1	=	0.1±0.1	=	0.1±0.0	<	0.1±0.0	=	0.1±0.0	=	0.1±0.0	<
18:3/16:0	0.2±0.3	=	0.1±0.1	=	0.2±0.2	=	+	=	+	=	+	=	+	=	+	=	+	=	+	=	0.1±0.1	=	+	=	+	=	+	=	+	=	+	=
18:3/16:0	0.2±0.2	=	0.1±0.1	=	0.1±0.0	>	+	=	+	=	+	=	+	=	+	=	+	>	+	=	+	=	0.1±0.1	=	+	=	+	=	+	=	+	>
16:1/18:1	0.5±0.3	=	0.6±0.3	=	0.9±0.3	=	0.7±0.6	=	0.5±0.2	=	0.4±0.1	=	0.6±0.3	=	0.7±0.4	=	0.2±0.1	<	0.3±0.1	=	0.2±0.2	=	0.3±0.1	=	0.3±0.2	=	0.3±0.2	<	0.6±0.2	=	0.8±0.2	<
18:2/16:0	0.5±0.4	=	0.4±0.1	=	0.5±0.2	=	0.4±0.1	<	0.6±0.1	=	0.6±0.4	=	0.5±0.2	=	0.4±0.0	=	1.3±0.6	=	1.2±0.3	=	1.0±0.2	<	2.3±0.7	=	1.9±1.4	=	2.3±0.6	=	1.6±1.0	<	2.7±0.3	<
16:0/18:1	3.6±0.4	=	3.3±0.3	>	2.6±0.2	=	2.6±0.3	=	2.3±0.2	=	2.7±0.4	=	2.4±0.3	=	2.7±0.3	>	3.8±0.4	=	3.9±0.3	=	3.9±0.2	<	4.3±0.4	<	4.9±0.1	=	4.7±0.7	=	4.4±0.2	=	4.3±0.4	=
18:0/16:0	0.4±0.2	=	0.4±0.1	=	0.4±0.1	>	0.2±0.1	=	0.1±0.0	=	0.1±0.0	=	0.1±0.1	=	0.2±0.1	>	0.3±0.1	=	0.3±0.1	=	0.3±0.1	=	0.2±0.0	<	0.3±0.1	=	0.2±0.1	=	0.1±0.1	=	0.1±0.0	>
17:1/18:1	0.2±0.0	>	+	=	0.1±0.0	=	+	=	0.1±0.0	=	+	=	0.1±0.0	=	+	>	+	=	+	=	+	=	+	=	0.2±0.1	=	0.1±0.1	=	0.1±0.1	=	+	=
17:0/18:1	0.2±0.1	=	0.5±0.4	=	0.1±0.0	=	0.2±0.2	=	0.1±0.0	=	0.1±0.0	=	0.1±0.1	<	0.3±0.2	=	0.2±0.1	>	0.1±0.0	=	0.1±0.1	=	0.1±0.0	=	0.1±0.1	=	0.2±0.1	=	0.2±0.0	<	0.4±0.0	<
18:3/18:3	0.1±0.0	>	-	<	+	=	0.1±0.0	>	+	=	+	=	+	=	+	>	+	=	+	=	+	=	+	=	+	=	+	=	+	=	+	=
18:2/18:4	0.1±0.0	=	0.1±0.0	>	+	<	0.1±0.0	=	0.1±0.1	=	0.1±0.1	=	0.1±0.0	>	+	=	+	=	+	<	0.2±0.1	=	0.3±0.1	>	0.1±0.1	=	0.1±0.1	=	+	=	0.1±0.1	=
16:1/20:5	+	=	+	=	0.1±0.0	=	+	=	+	=	+	=	+	=	+	=	+	=	+	=	+	=	+	>	+	<	+	=	+	=	+	=
18:2/18:3	+	=	+	<	0.1±0.0	>	+	=	+	=	+	=	+	=	+	>	+	=	+	=	+	=	+	=	0.1±0.0	=	+	=	+	=	+	=
18:4/18:1	0.2±0.2	=	+	=	+	=	0.1±0.1	=	+	=	+	=	+	=	+	=	+	=	+	<	0.1±0.0	>	+	=	+	=	+	=	+	=	+	=
18:2/18:2	0.2±0.1	=	0.1±0.1	=	0.3±0.4	=	0.2±0.1	=	0.1±0.1	=	0.1±0.0	=	0.1±0.0	=	0.1±0.0	>	0.1±0.0	<	0.3±0.1	=	0.3±0.1	=	0.3±0.2	=	0.1±0.1	=	0.1±0.0	=	0.1±0.0	=	0.1±0.0	>
18:1/18:2	14.7±0.7	=	14.8±0.6	=	15.7±4.2	=	12.7±1.4	=	11.6±0.7	=	11.4±1.2	=	10.1±1.6	<	12.3±0.8	>	18.9±0.8	=	19.7±0.9	<	21.4±1.0	=	21.4±1.9	>	12.0±0.6	=	11.8±1.2	=	11.5±0.8	<	12.7±0.5	>
18:1/18:1	38.8±2.3	=	41.6±3.0	<	49.9±4.3	<	55.7±3.2	<	60.7±3.1	=	61.0±1.9	=	61.6±5.9	=	61.4±2.8	<	43.7±1.1	>	42.1±1.0	<	46.5±1.7	>	44.4±0.8	<	63.8±2.9	=	67.9±3.1	=	67.9±2.9	=	68.7±2.0	<
16:0/20:1, 18:1/18:0	24.6±1.8	=	24.1±3.2	>	15.5±2.5	=	15.7±0.9	>	9.2±0.7	=	8.4±1.0	=	8.8±0.8	>	6.2±0.7	>	23.6±1.4	=	23.5±1.3	>	16.5±1.9	=	16.6±3.7	>	6.9±1.2	=	5.0±1.5	=	3.9±0.6	>	2.6±0.4	>
18:0/18:0	0.1±0.1	=	0.1±0.0	=	+	=	0.1±0.0	>	+	=	+	=	+	=	+	>	0.1±0.0	=	0.1±0.0	=	0.1±0.0	=	0.1±0.0	=	0.1±0.0	=	+	=	+	=	0.1±0.1	=
18:1/19:1	3.9±1.2	=	4.4±0.4	=	5.3±1.0	=	4.5±0.9	>	3.2±0.8	<	4.7±0.7	=	4.1±2.1	=	4.6±0.9	=	0.4±0.1	>	0.3±0.1	<	1.1±0.1	=	1.0±0.9	=	0.5±0.2	=	0.5±0.3	=	0.7±0.1	=	0.7±0.1	<
19:1/18:1	1.0±0.2	=	1.0±0.2	=	0.9±0.2	=	0.8±0.2	=	0.7±0.2	=	0.5±0.2	=	0.6±0.2	=	0.6±0.1	>	0.1±0.0	=	0.2±0.1	=	0.1±0.0	=	0.2±0.1	=	0.3±0.1	=	0.3±0.1	=	0.5±0.1	=	0.4±0.1	<
18:3/20:3	+	=	0.1±0.1	=	0.2±0.3	=	+	=	+	=	+	=	+	=	+	=	+	=	+	=	+	=	+	=	+	=	+	=	+	>	+	>
18:1/20:5	0.1±0.0	=	0.1±0.1	=	0.1±0.1	=	0.1±0.0	>	+	=	+	=	+	=	+	=	+	=	+	=	0.1±0.1	=	+	=	+	=	+	=	+	=	+	=
16:0/22:6	0.1±0.0	=	+	=	0.1±0.0	=	+	=	+	=	0.1±0.0	>	+	=	+	>	+	=	+	=	+	=	+	<	0.1±0.0	>	+	=	+	<	+	<
18:1/20:1	4.3±0.8	=	4.5±0.5	>	3.9±0.2	>	3.2±0.3	>	1.9±0.3	>	1.4±0.2	=	1.6±0.3	>	1.0±0.1	>	2.7±0.2	=	2.7±0.2	>	1.7±0.1	=	1.7±0.4	>	0.7±0.2	>	0.3±0.0	=	0.4±0.1	>	0.2±0.1	>
18:1/20:0	1.3±0.3	=	0.8±0.4	=	0.4±0.3	=	0.5±0.1	>	0.1±0.1	=	0.2±0.1	=	0.2±0.1	>	0.1±0.0	>	0.9±0.3	=	0.9±0.2	>	0.4±0.2	=	0.6±0.3	>	0.1±0.1	=	+	=	0.1±0.0	=	+	>

Figure S1. Identification of the endophytic algae *Streblonema corymbiferum* and *Streblonema* sp.

Streblonyma corymbiferum grown inside *Undaria pinnatifida*

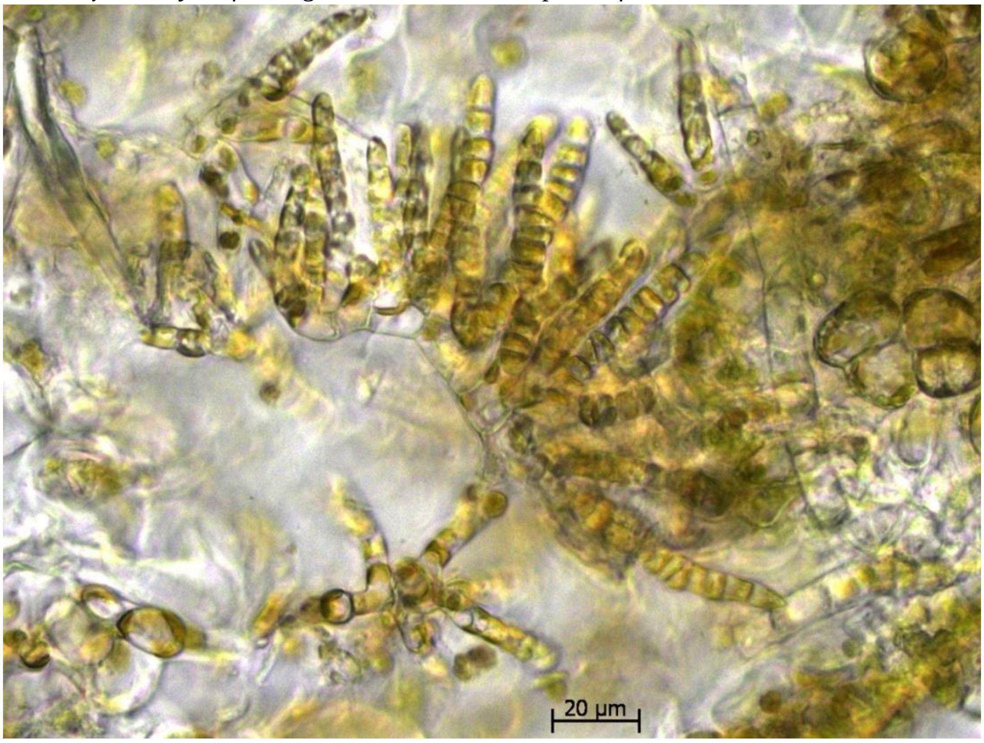

Streblonema sp. grown inside *Undaria pinnatifida* shares features of the genus

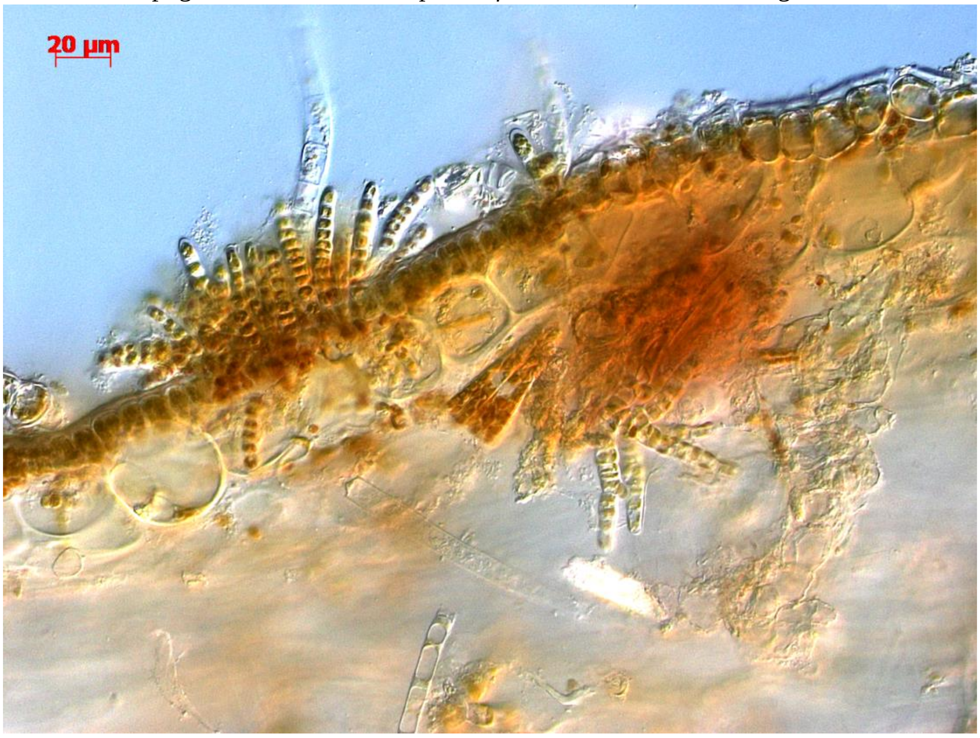

To identify the endophytes we observe they morphology in free-living culture and inside the host (*Undaria pinnatifida*). The obtaining and maintenance of free-living cultures is described in the Materials and Methods section (see Section 4.1). The blade's discs of *Undaria pinnatifida* (Laminariales, Ochrophyta) were infected with *Streblonema sp.* and *S. corymbiferum* separately. Undaria was collected in February of 2016 in Sobol Bay (Sea of Japan). Blades without visible signs of endophitic infection (such as darker spots, deformation and perforations) were selected. Fragments of middle part of the blades were cuted, washed with sterilized seawater using a brush. Then, cross-sections were made in different parts of fragments to check for the presence of endophytic algae. The cross-sections were inspected under AxioVert 2000 microscope. Fragments without endophytes were chosen. Discs 3 cm in diameter were prepared using a cork drill. The discs were placed in the 100 mL glasses separately. The suspension of free-living cultures of endophytic algae were added. After one week cross-sections were made from margin of the

discs and were checked under microscope. Discs infected with endophytes were cultured during one month under conditions described in section 4.1. When endophyte became well-developed and form dark spots on discs, cross-sections were made and checked. Endophytes were identified using descriptions of filamentous ectocarpalean algae [1-6].

Both species shared all the features of the species: deeply penetrating into the host's tissue curved uniseriate filaments of cells of irregular shape, 3–5 µm wide, single band-shaped chloroplast without pyrenoid, uniseriate cylindrical plurangia. In *Streblonema corymbiferum* endophytic filaments were crowded or corymbous branched, plurangia formed dense corymbous clusters near the host surface, which were characteristic of the species. The name *Streblonema* sp. was used in the present study because to date, due to the complexity of specific morphological features, this alga has not been certainly assigned to any of the described species of the genus. Plurangia of the species never formed corymbous clusters, they were unbranched and growed solitary from cells of creaping filaments.

References

1. Abbott, I.A., Hollenberg, G.J. *Marine algae of California*. Stanford University Press: Stanford, CA, USA, 1976, p. 827.
2. Luan R., Ding L., Lu B., Tseng, C.K. *Flora algarum marinarum sinicarum. Tomus III. Phaeophyta No. I(1) Ectocarpales Ralfsiales Sphaceariales Dictyotales*; Science Press: Beijing, China, 2013. [In Chinese].
3. Norris, J.N. Marine algae of the Northern Gulf of California: Chlorophyta and Phaeophyceae. *Smithson. Contrib. Bot.* **2010**, 94:1–94:276, doi.org/10.5479/si.0081024X.94.276.
4. Perestenko, L.P. *Vodorosli Zaliva Petra Velikogo [The seaweeds of Peter the Great Bay]*. NAUKA Leningradskoe Otdelenie: Leningrad, Russia, 1980, p. 231. [In Russian].
5. Tseng, C.K. *Seaweeds in Yellow Sea and Bohai Sea of China*. Science Press: Beijing, China, 2009, ISBN: 9787030249968. [In Chinese].
6. Womersley, H.B.S. *The Marine Benthic Flora of Southern Australia, Part II*. Government Printer: Adelaide, South Australia, 1987.

Table S3. MS parameters for polar lipids molecular species quantification and identification.

Lipid class	Quntitation parameters				Identification parameters			Ref.
	Precursor ion	Lipid class specific fragmentation reaction	Collision energy, V	Scan range, m/z	Precursor ion	Collision energy, V	Most abundant characteristic fragment	
MGDG	$[M+NH_4]^+$	Loss of 179.1	18	454-918	$[M+Li]^+$	37	FA loss at sn-1	[46]
DGDG	$[M+NH_4]^+$	Loss of 341.1	21	616-1080	$[M+Li]^+$	46	FA loss at sn-1	[46]
SQDG	$[M+NH_4]^+$	Loss of 261.1	32	518-982	$[M-H]^-$	50	FA loss at sn-1	[47]
GlcADG	$[M+NH_4]^+$	Loss of 211.1	22	468-932	$[M-H]^-$	36	$[FA-H]^-$*	-
PG	$[M+NH_4]^+$	Loss of 189.0	25	446-910	$[M-H]^-$	40	$[FA-H]^-$ from sn-2	[48, 49]
PI	$[M-H]^-$	Product 241.0	44	515-979	$[M-H]^-$	40	FA loss at sn-2, $[FA-H]^-$ from sn-1	[48, 49]
PHEG	$[M+H]^+$	Loss of 199.0	26	456-920	$[M+H]^+$	32	$[FA+C_3H_5O]^+$*	-
PE	$[M+H]^+$	Loss of 141.0	24	398-862	$[M-H]^-$	35	$[FA-H]^-$ from sn-2, keten loss at sn-2	[48, 49]
PC	$[M+H]^+$	Product 184.1	33	440-904	$[M+Li]^+$	40	$FA+N(CH_3)_3$ loss at sn-1	[49]
DGTS	$[M+H]^+$	Product 236.1	50	418-882	$[M+H]^+$	35	Keten loss at sn-1	[50]

*sn-positions of acyl chains for GlcADG and PHEG have not been determined.

Figure S2. Mass-spectra and MS/MS fragmentation schemes of polar lipid classes.

MGDG 16:0/18:1, precursor ion $[M+Li]^+$, m/z 763.6

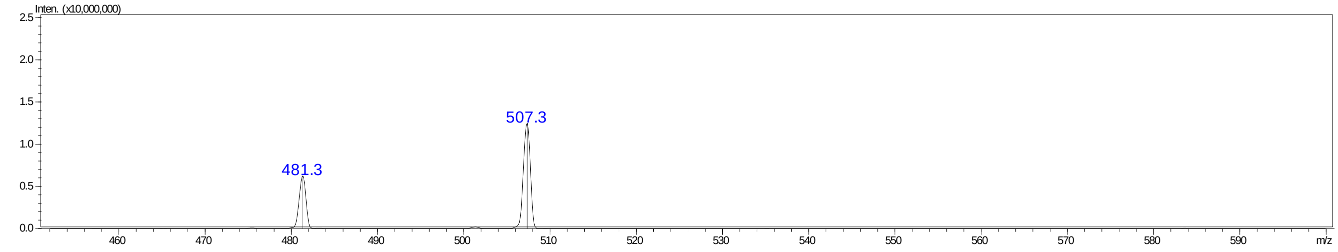

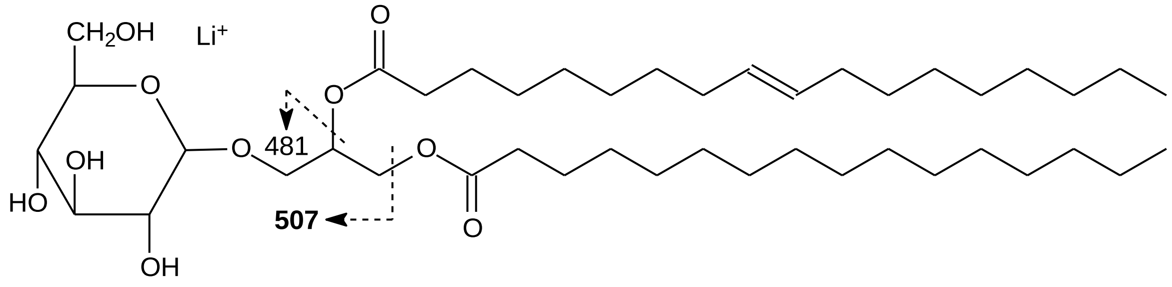

DGDG 14:0/18:3, precursor ion $[M+Li]^+$, m/z 893.6

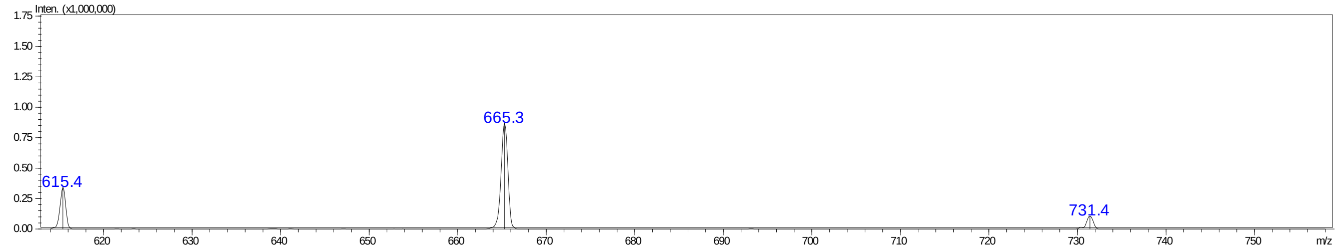

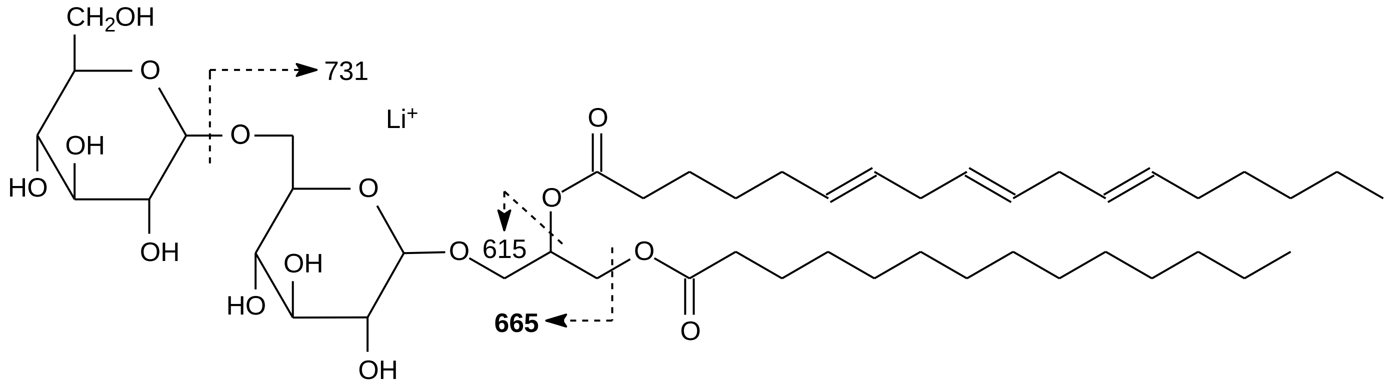

SQDG 14:0/18:3, precursor ion $[M-H]^-$, m/z 787.5

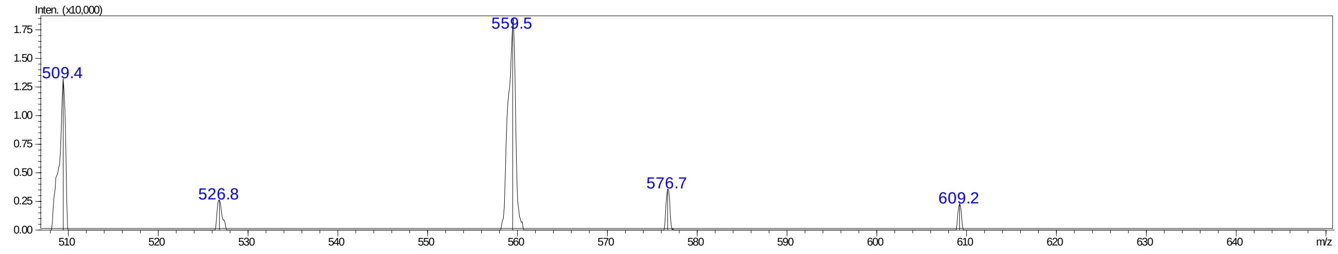

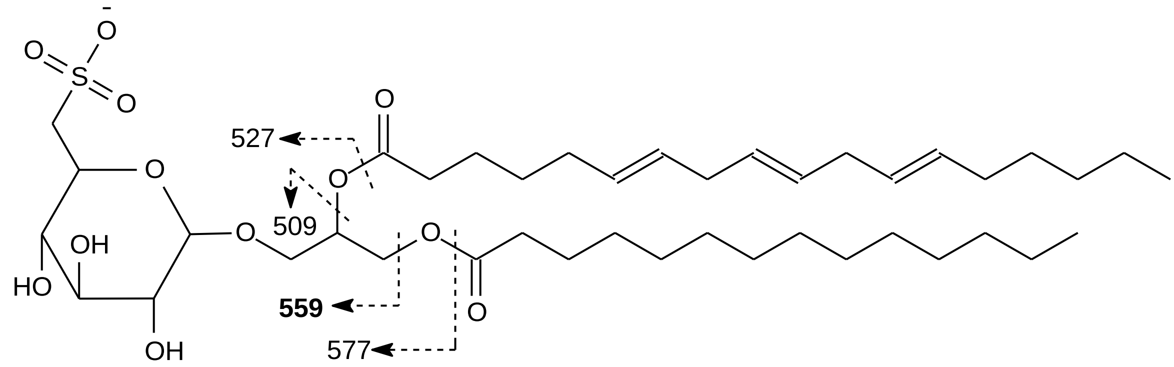

GlcADG 18:1/18:2, precursor ion [M-H]-, m/z 793.5 *(sn-position of acyl chains have not been determined)

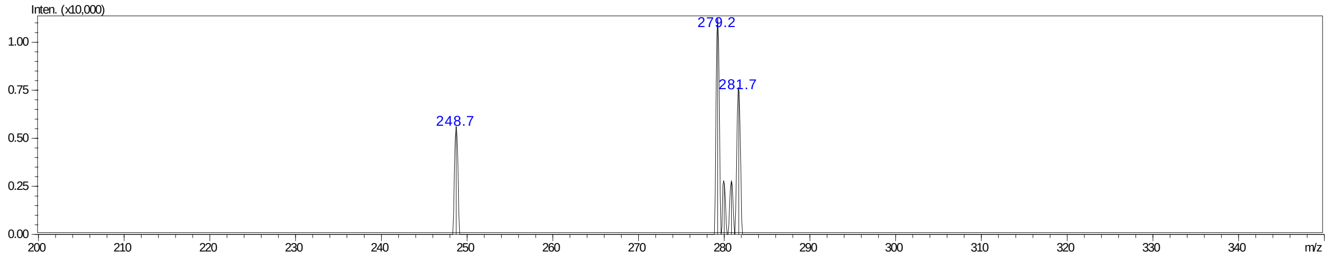

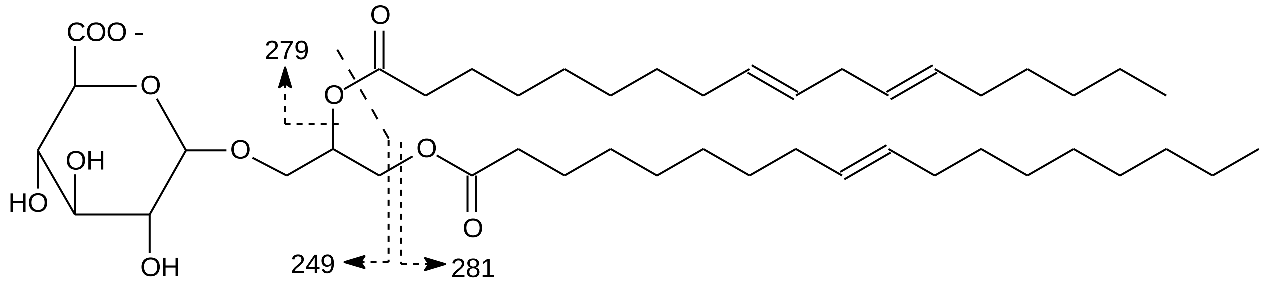

PG 18:2/16:0, precursor ion [M-H]-, m/z 745.5

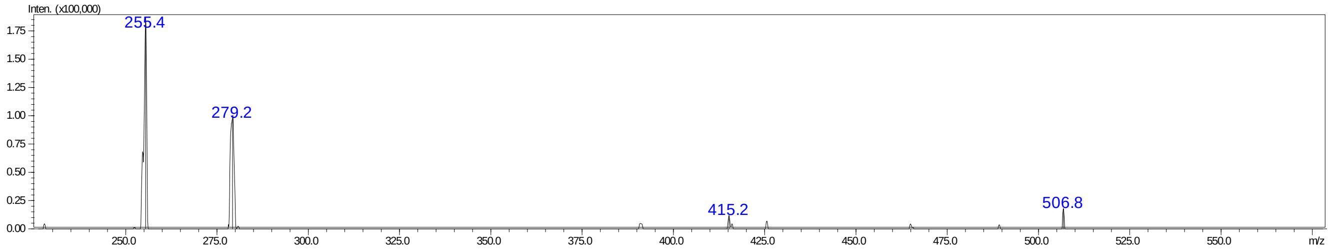

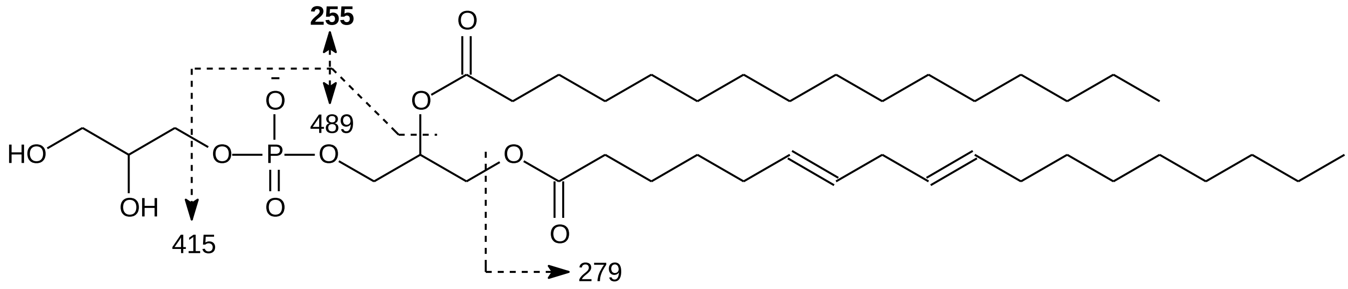

PG 16:0/18:2, precursor ion [M-H]-, m/z 745.5

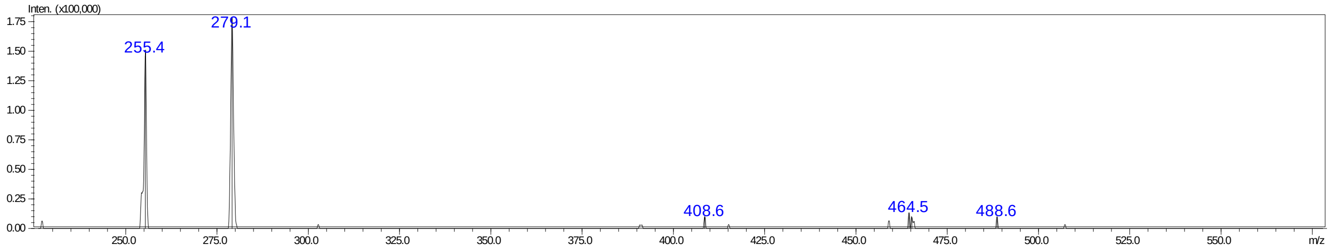

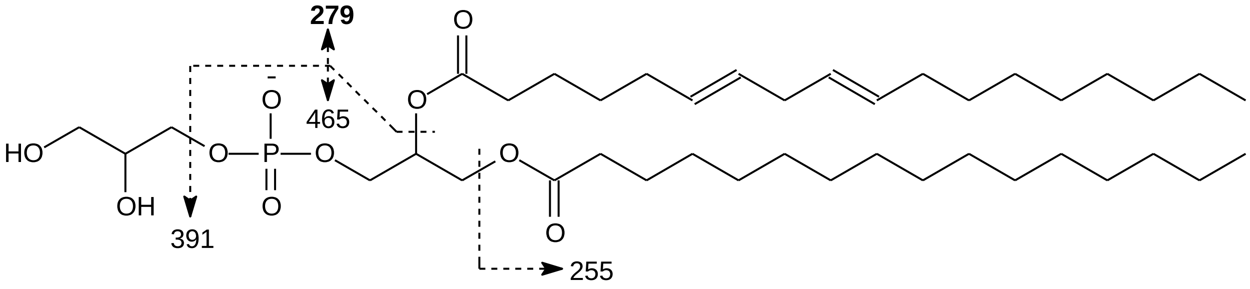

PI 16:0/18:3, precursor ion [M-H]$^{-}$, m/z 831.5

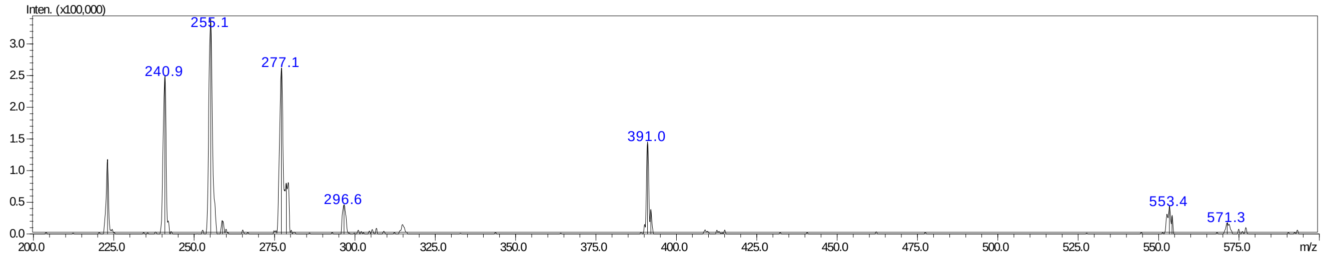

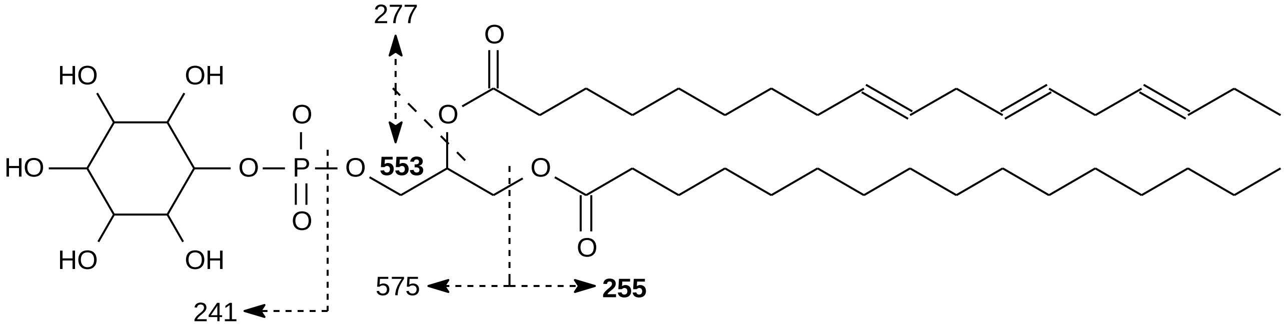

PHEG 20:5/20:4, precursor ion [M+H]$^{+}$, m/z 764.5 *(sn-position of acyl chains have not been determined)

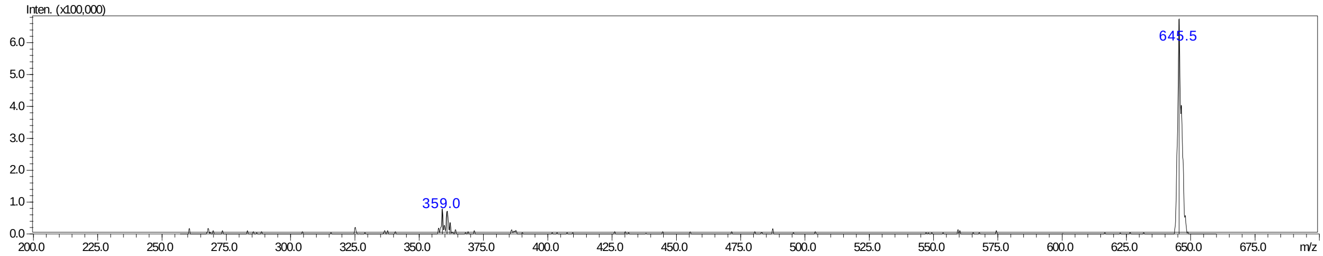

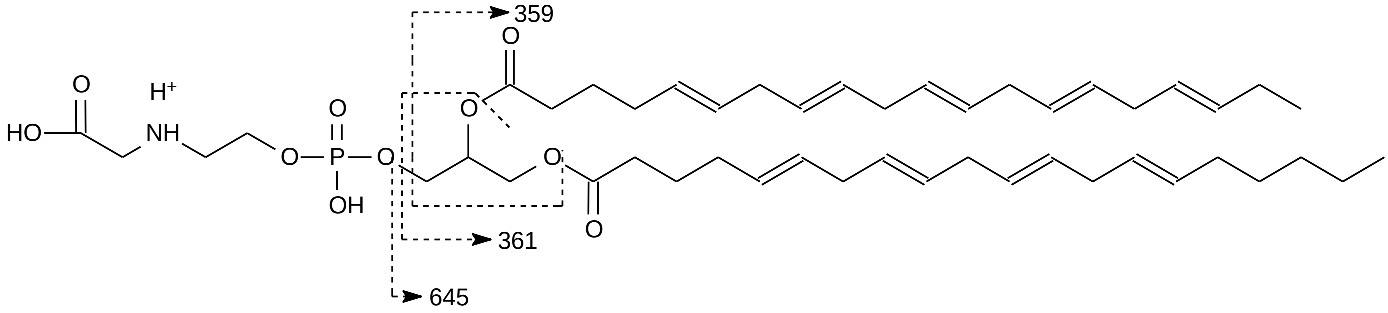

$C_{23}H_{37}O_3$
361.27

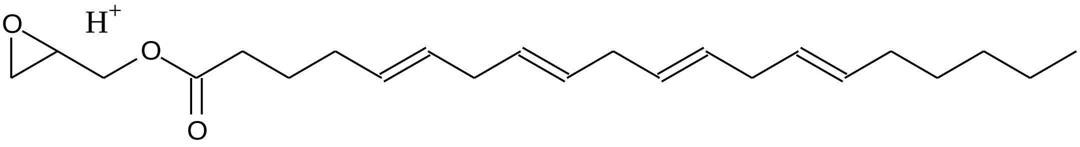

PE 18:1/20:4, precursor ion [M-H]$^{-}$, m/z 764.5

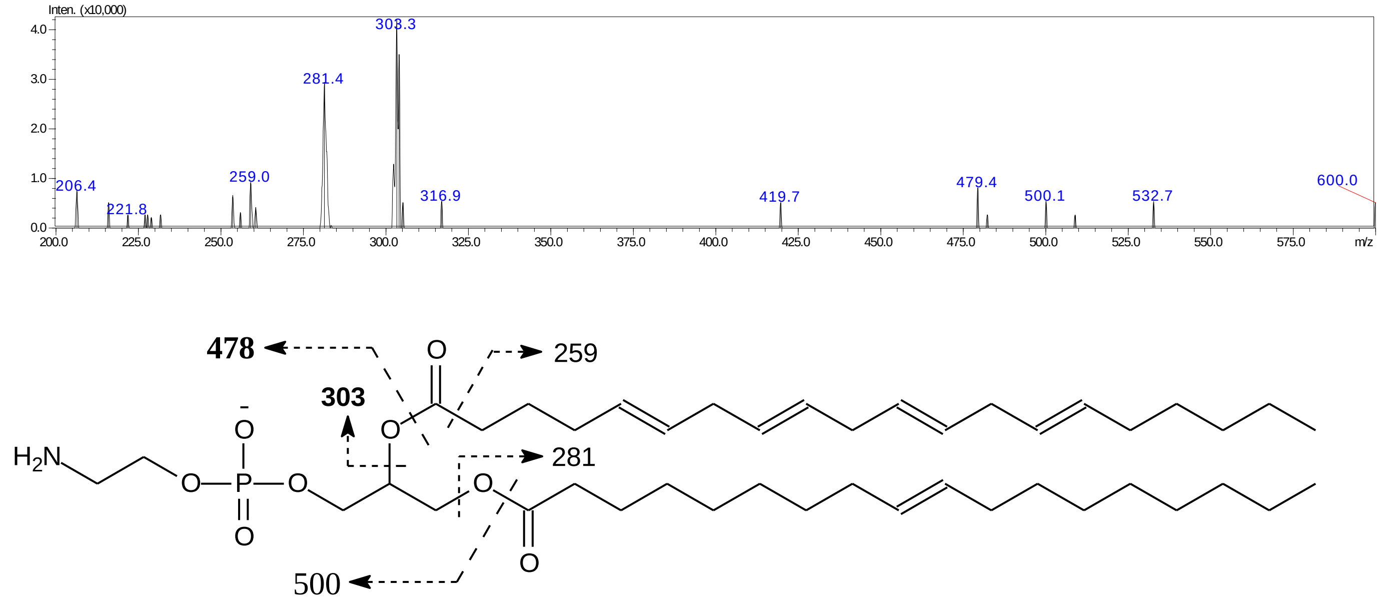

PE 18:1/16:1, precursor ion [M-H]$^-$, m/z 714.5

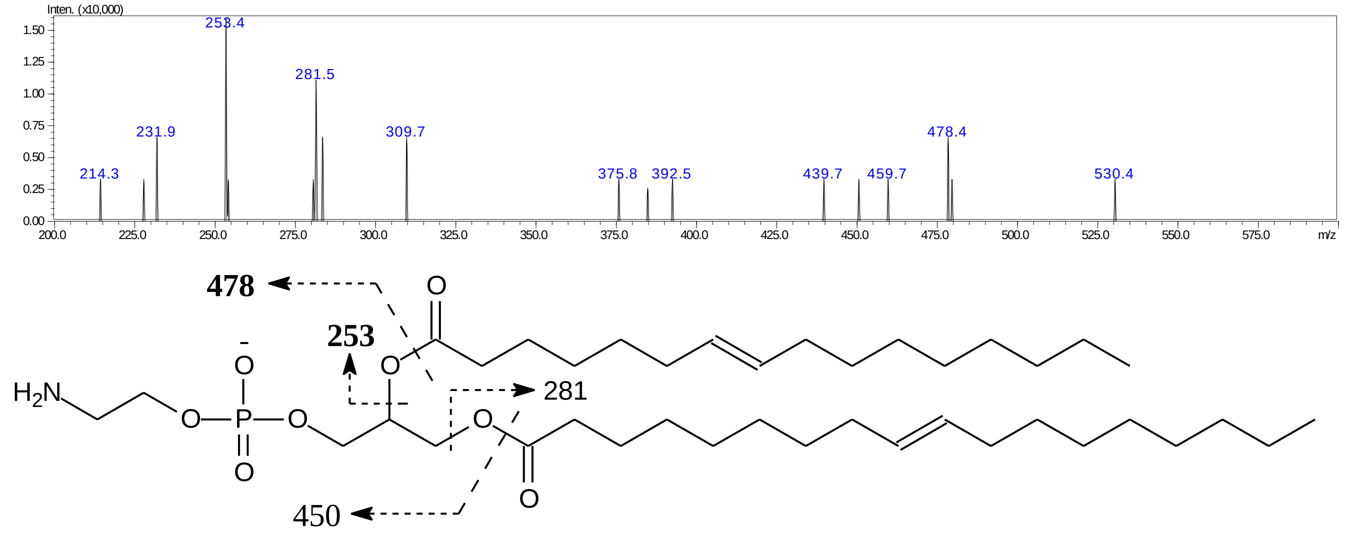

PC 16:0/18:2, precursor ion [M+Li]$^+$, m/z 764.6

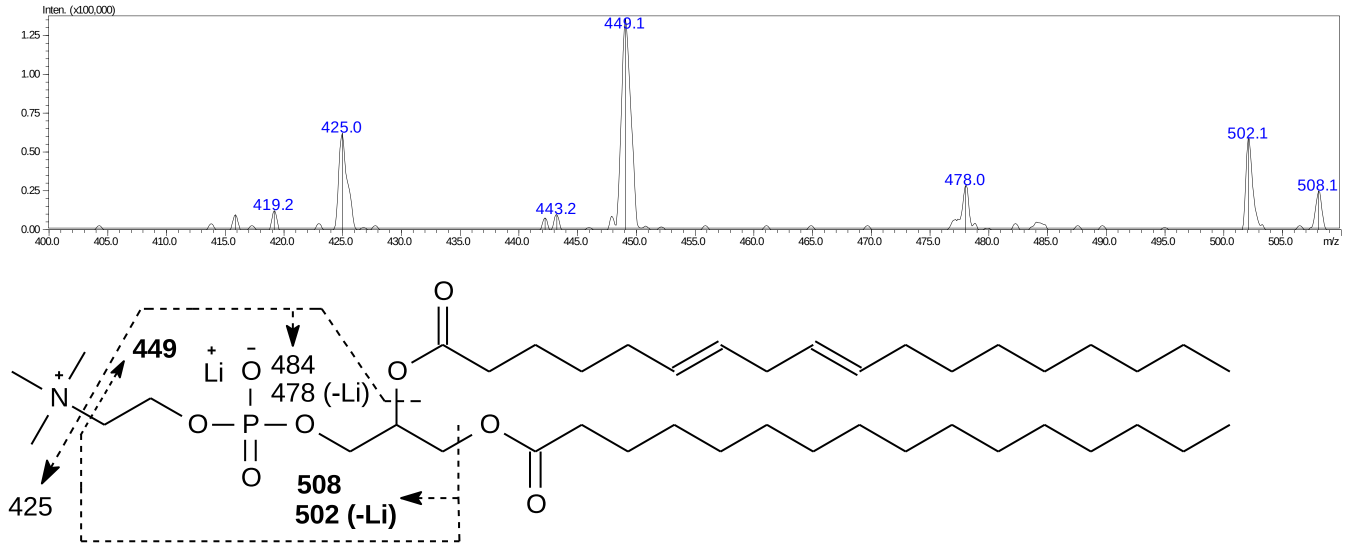

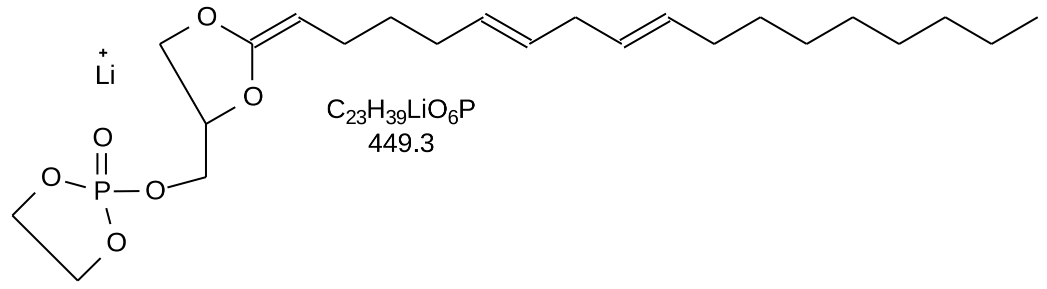

DGTS 18:1/19:1, precursor ion $[M+H]^+$, m/z 778.7

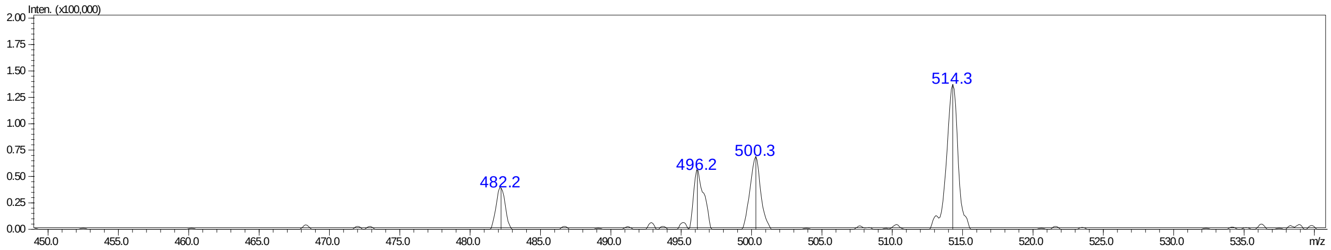

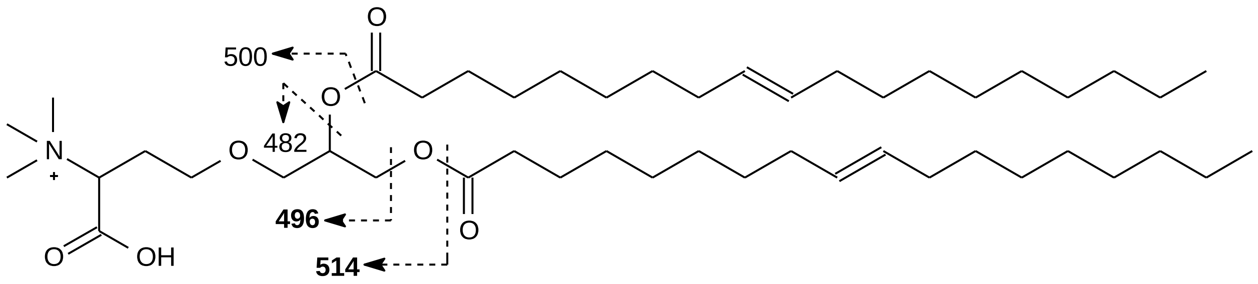

Supplement: Supplementary file 1 [file marinedrugs-20-00428-s001.zip › marinedrugs-1773755-supplementary.pdf]
